# Modulation of the Serotonergic Receptosome in the Treatment of Anxiety and Depression: A Narrative Review of the Experimental Evidence

**DOI:** 10.3390/ph14020148

**Published:** 2021-02-12

**Authors:** Gustavo R. Villas-Boas, Stefânia N. Lavorato, Marina M. Paes, Pablinny M. G. de Carvalho, Vanessa C. Rescia, Mila S. Cunha, Manoel F. de Magalhães-Filho, Luis F. Ponsoni, Adryano Augustto Valladao de Carvalho, Roseli B. de Lacerda, Lais da S. Leite, Matheus da S. Tavares-Henriques, Luiz A. F. Lopes, Luiz G. R. Oliveira, Saulo E. Silva-Filho, Ana P. S. da Silveira, Roberto K. N. Cuman, Francielli M. de S. Silva-Comar, Jurandir F. Comar, Luana do A. Brasileiro, Jussileide N. dos Santos, William R. de Freitas, Katyuscya V. Leão, Jonatas G. da Silva, Raphael C. Klein, Mary H. F. Klein, Bruno H. da S. Ramos, Cristiane K. C. Fernandes, Dayane G. de L. Ribas, Silvia A. Oesterreich

**Affiliations:** 1 Research Group on Development of Pharmaceutical Products (P & DProFar), Center for Biological and Health Sciences, Federal University of Western Bahia, Rua Bertioga, 892, Morada Nobre II, Barreiras CEP 47810-059, BA, Brazil; stefania.lavorato@ufob.edu.br (S.N.L.); marinameirelles@ymail.com (M.M.P.); pablinny.galdino@ufob.edu.br (P.M.G.d.C.); vanessa.rescia@ufob.edu.br (V.C.R.); milacunha035@gmail.com (M.S.C.); manoel.magalhaes17@gmail.com (M.F.d.M.-F.); luis.p7776@ufob.edu.br (L.F.P.); adryano.carvalho@ufob.edu.br (A.A.V.d.C.); 2 Department of Pharmacology, Center for Biological Sciences, Federal University of Paraná, Jardim das Américas, Caixa. postal 19031, Curitiba CEP 81531-990, PR, Brazil; boerngen@hotmail.com; 3 Collegiate Biomedicine, SulAmérica College, Rua Gláuber Rocha, 66, Jardim Paraíso, Luís Eduardo Magalhães CEP 47850-000, BA, Brazil; laisleite@sulamericafaculdade.edu.br; 4 Laboratory of Pharmacology of Toxins (LabTox), Graduate Program in Pharmacology and Medicinal Chemistry (PPGFQM), Institute of Biomedical Sciences (ICB) Federal Universityof Rio de Janeiro (UFRJ), Avenida Carlos Chagas Filho, 373, Cidade Universitária, Rio de Janeiro CEP 21941-590, RJ, Brazil; matheus-sth@hotmail.com; 5 Teaching and Research Manager at the University Hospital—Federal University of Grande Dourados (HU/EBSERH-UFGD), Federal University of Grande Dourados, Rua Ivo Alves da Rocha, 558, Altos do Indaiá, Dourados CEP 79823-501, MS, Brazil; luiz.lopes@ebserh.gov.br; 6 Nucleus of Studies on Infectious Agents and Vectors (Naive), Federal University of Western Bahia, Rua Bertioga, 892, Morada Nobre II, Barreiras CEP 47810-059, BA, Brazil; luiz.oliveira@ufob.edu.br; 7 Pharmaceutical Sciences, Food and Nutrition College, Federal University of Mato Grosso do Sul, Avenida Costa e Silva, s/n°, Bairro Universitário, Campo Grande CEP 79070-900, MS, Brazil; saulo.e@ufms.br; 8 Faculty of Biological and Health Sciences, Unigran Capital University Center, RuaBalbina de Matos, 2121, Jarddim Universitário, Dourados CEP 79.824-900, MS, Brazil; anapaulastefanello@hotmail.com; 9 Department of Pharmacology and Therapeutics, State University of Maringá, Avenida Colombo, n° 5790, Jardim Universitário, Maringá CEP 87020-900, PR, Brazil; rkncuman@uem.br (R.K.N.C.); franciellimss@gmail.com (F.M.d.S.S.-C.); 10 Department of Biochemistry, State Universityof Maringá, Avenida Colombo, n° 5790, Jardim Universitário, Maringá CEP 87020-900, PR, Brazil; jfcomar@uem.br; 11 Nacional Cancer Institute (INCA), Rua Visconde de Santa Isabel, 274, Rio de Janeiro CEP 20560-121, RJ, Brazil; luana.brasileiro@inca.gov.br; 12 Natu Flora, Rua José Rocha, n° 239, Barreiras CEP 47.800-184, BA, Brazil; leide-neves@hotmail.com; 13 Research Group on Biodiversity and Health (BIOSA), Center for Training in Health Sciences, Federal University of Southern Bahia, Praça Joana Angélica, 58, São José, Teixeira de Freitas CEP 45988-058, BA, Brazil; william.freitas@ufsb.edu.br; 14 Pharmacy Department, Federal University of Western Bahia, Rua Bertioga, 892, Morada Nobre II, Barreiras CEP 47810-059, BA, Brazil; kleao@ufob.edu.br (K.V.L.); gomes.jonatas@gmail.com (J.G.d.S.); raphael.klein@ufob.edu.br (R.C.K.); mary.klein@ufob.edu.br (M.H.F.K.); 15 Institute of the Spine and Pain Clinic, Rua Dr. Renato Gonçalves, 108, Renato Gonçalves, Barreiras CEP 47806-021, BA, Brazil; Drbrunoramos@hotmail.com; 16 University Center of Montes Belos, Av. Hermógenes Coelho s/n, Setor Universitário, São Luís de Montes Belos CEP 76100-000, GO, Brazil; cristianekarla01@hotmail.com; 17 Gaus College and Course, Rua Severino Vieira, 60, Centro, Barreiras CEP 47800-160, BA, Brazil; dayane-gaby@hotmail.com; 18 Faculty of Health Sciences, Federal University of Grande Dourados, Dourados Rodovia Dourados, Itahum Km 12, Cidade Universitaria, Caixa postal 364, Dourados CEP 79804-970, MS, Brazil; silviaoesterreich@gmail.com

**Keywords:** 5-HT receptors, anxiety, depression, G protein-coupled receptors, antidepressants, anxiolytics, phytochemicals

## Abstract

Serotonin (5-HT) receptors are found throughout central and peripheral nervous systems, mainly in brain regions involved in the neurobiology of anxiety and depression. 5-HT receptors are currently promising targets for discovering new drugs for treating disorders ranging from migraine to neuropsychiatric upsets, such as anxiety and depression. It is well described in the current literature that the brain expresses seven types of 5-HT receptors comprising eighteen distinct subtypes. In this article, we comprehensively reviewed 5-HT1-7 receptors. Of the eighteen 5-HT receptors known today, thirteen are G protein-coupled receptors (GPCRs) and represent targets for approximately 40% of drugs used in humans. Signaling pathways related to these receptors play a crucial role in neurodevelopment and can be modulated to develop effective therapies to treat anxiety and depression. This review presents the experimental evidence of the modulation of the “serotonergic receptosome” in the treatment of anxiety and depression, as well as demonstrating state-of-the-art research related to phytochemicals and these disorders. In addition, detailed aspects of the pharmacological mechanism of action of all currently known 5-HT receptor families were reviewed. From this review, it will be possible to direct the rational design of drugs towards new therapies that involve signaling via 5-HT receptors.

## 1. Introduction

The first two subtypes of serotonergic receptors, serotonin receptors (5-HT) D and M, were discovered in 1957 [1]. Since then, a combination of pharmacological and neurochemical investigations has culminated in the discovery of a diversity of other types of 5-HT receptors. Currently, it has been well established in literature that the brain expresses seven types of 5-HT (5-HT_1-7_) receptors, comprising a total of eighteen distinct subtypes (Table S1 (Supplementary Material)), although two of these receptors, 5-ht_1e_ and 5-ht_5b_, keep the denomination in lower case and are classified as gene products, since they have not been associated with a specific function in native cells and/or tissues. Regarding the location of these receptors, most subtypes of 5-HT receptors are found both in the central and peripheral nervous system and in other cell types, such as smooth muscle, gastrointestinal tract, and platelets. The only exceptions are 5-ht_1e_, 5-HT_2C_, and 5-HT_6_ receptors, for which there is limited expression outside the central nervous system (CNS). This diversity of receptors is comparable to that of more complex neurotransmitter systems, including glutamate and purines. Despite this, a wide range of agonists, antagonists, and selective radioligands are available for each subtype of 5-HT receptors, which has allowed further investigation of their location, signaling properties and functions in the CNS and in the rest of the organism [2].

Given this scenario, this review presents, in a narrative context, the experimental evidence for the modulation of the “serotonergic receptosome” in the treatment of anxiety and depression, as well as demonstrating state-of-the-art research related to phytochemicals and these disorders. In addition, a detailed review of aspects related to the mechanism of action of all currently known families of 5-HT receptors was carried out. The present study represented a crucial milestone to direct the rational development of drugs and, consequently, new therapies that involve signaling through serotonergic receptors.

## 2. 5-HT_1_ Receptors

### 2.1. Mechanism of Pharmacological Action

The five subtypes of 5-HT_1_ serotonergic receptors are metabotropic coupled to the inhibitory G protein (Gα_i/o_); that is, the receptor activation inhibits the adenylate cyclase enzyme (AC) and the formation of adenosine 3’,5’-cyclic monophosphate (cAMP) [3].

**Figure 1:**
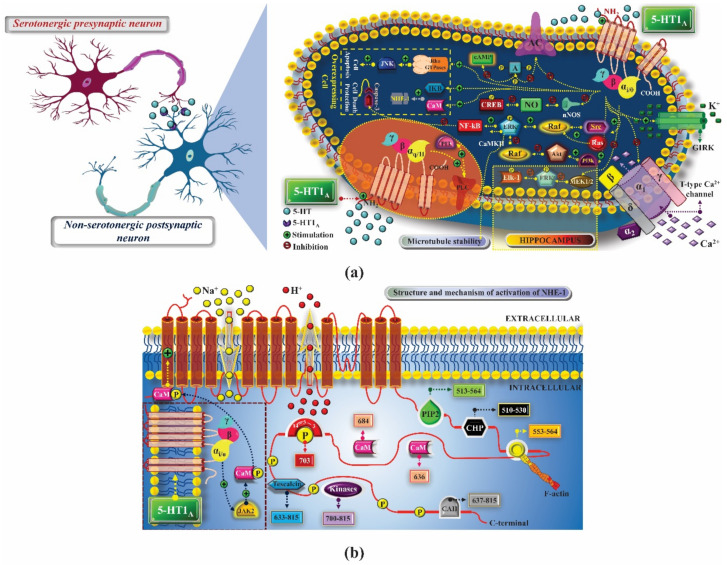
Serotonin (5-HT_1A_) receptor signaling pathways: (**a**) The 5-HT_1A_ receptor is coupled to the Gα_i/0_ protein. Its activation blocks the adenylate cyclase activity (AC), reducing the conversion of adenosine triphosphate (ATP) into cyclic adenosine monophosphate (cAMP), which is responsible for the activation of protein kinase A (PKA). The activation of these receptors decreases the release of neurotransmitters in neurons through opposite changes in K^+^ (increase) and Ca^2+^ (decrease) conductances. In addition, stimulation of the 5-HT_1A_ receptor regulates the phosphorylation of the extracellular signal-regulated kinase (ERK). The “atypical” coupling is represented by the orange circle and demonstrates the overexpressing cell Jurkat T-like cell line. The yellow rectangle represents a specific effect on the rat hippocampus, where stimulation of the 5-HT1A receptor reduced MEK1/2 and ERK and Elk-1 phosphorylation. This change in pERK levels was not seen in the cortex [12]; (**b**) the activation of the 5-HT_1A_ receptor also induces stimulation of the Janus kinase 2 protein (JAK2), which phosphorylates the calmodulin protein, which in turn is associated with the sodium and hydrogen 1 (NHE-1) transporter, activating it. The massive outlet of protons caused by the 5-HT_1A_ activation culminates in an increase in intracellular pH. The numbers represent the amino acids (approximate region) of the cytoplasmic domain of the NHE1 transporter in which the other proteins interact.

5-HT_1A_ receptors are distributed in the limbic, cortical, and dorsal and median raphe nucleus (DRN and MRN), and are expressed in the pre- and post-synaptic membrane of neurons, where they act by regulating the extracellular serotonin concentration (5-HT) and transmission of the action potential. As an autoreceptor, it promotes the activation of internal rectifying potassium channels (GIRK), which allow an increase in potassium conductance, leading to neuron hyperpolarization, which in turn inhibits the opening of voltage-dependent calcium channels and the release of 5-HT [2,4]. In the post-synaptic membrane of non-serotonergic neurons, 5-HT_1A_ heteroreceptors, when stimulated by an agonist, activate the signal transduction pathway mediated by the Gα_i/0_ protein, which decreases cAMP synthesis and the activation of protein kinase A (PKA), which is responsible for the phosphorylation of proteins and enzymes downstream, such as the cAMP-responsive binding protein (CREB), an intranuclear transcription factor (Figure 1a). The activation of these post-synaptic receptors also promotes hyperpolarization, similar to what occurs in pre-synaptic 5-HT_1A_ receptors, reducing neuronal hyperactivity, important for the treatment of some diseases such as anxiety [5] and schizophrenia [6]. By a mechanism not yet known, the activation of this receptor is also related to an increase in the number of reactive oxygen and nitrogen species, and stimulation of an enzyme similar to nicotinamide adenine dinucleotide phosphate oxidase (NADPH-oxidase) [7,8,9,10].

Another mechanism associated with the 5-HT_1A_ receptor activity involves the activation of extracellular signal-regulated kinases 1 and 2 (ERK1/2) and protein kinase B (AKT), with increased dendritic growth in hippocampal neurons after stimulation of serotonergic receptors 5-HT_1A_ and 5-HT_7_ [11]. The activation of the receptor leads to the same transduction pathway that culminates in the activation of ERK1/2 and AKT, which are important for the reorganization of the cytoskeleton. The activation of ERK increases the activity of nuclear factor kappa B (NF-κB), which inhibits caspase 3, preventing cell death. Calmodulin-dependent protein kinase II (CaMKII), which is stimulated by active ERK, is also involved in the signal transduction pathway of the 5-HT_1A_ receptor and can promote the destabilization of microtubules. In turn, after stimulating the 5-HT_1A_ receptor, AKT activation is mediated by phosphatidylinositol-3-kinase (PI3K) and stimulated by negative feedback when there are high levels of active ERK. This increase in active AKT promotes the inhibition of Raf, the kinase responsible for phosphorylating and activating ERK, thus reducing the ERK concentration. In contrast to the anti-apoptotic activity, the activation of 5-HT_1A_ is also related to the increase in the activity of the apoptosis-inducing c-Jun N-terminal kinase protein (JNK). The stimulation of 5-HT_1A_ induces the activation of the Janus kinase 2 protein (JAK2), which phosphorylates calmodulin (CaM), which is associated with the sodium and hydrogen transporter 1 (NHE-1), causing its activation and an increase in intracellular pH with proton output (Figure 1b) [12,13,14]. It is important to highlight that the balance between these activities and the final effect is dependent on the tissue analyzed.

**Figure 2:**
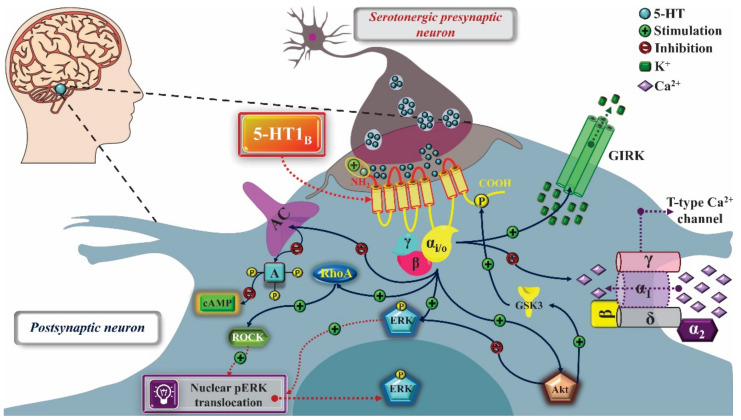
5-HT_1B_ receptor signaling pathways. The 5-HT_1B_ receptor is coupled to the Gα_i/0_ protein. Its activation blocks the activity of adenylate cyclase (AC), reducing the conversion of adenosine triphosphate (ATP) into cyclic adenosine monophosphate (cAMP), which is responsible for the activation of protein kinase A (PKA). The activation of these receptors decreases the release of neurotransmitters in neurons through opposite changes in K^+^ (increase) and Ca^2+^ (decrease) conductances. After 5-HT_1B_ activation, a cascade of kinases regulates the translocation of extracellular signal regulated kinase (ERK) (transcription activation) [12]. In addition, kinase B protein (AKT) is stimulated with consequent activation of glycogen synthase kinase 3 (GSK3), which is involved with phosphorylation and regulation of the 5-HT_1B_ receptor activity [16].

The activation of the 5-HT_1B_ serotonergic receptor also promotes the modulation of calcium and potassium channels, increasing the potassium conductance, hyperpolarizing the cell and, indirectly, reducing the calcium influx, which inhibits the release of 5-HT, when the 5-HT_1B_ receptor is expressed in the pre-synaptic membrane. As a post-synaptic receptor, 5-HT_1B_ inhibits AC through the signaling pathway triggered by the Gα_i/0_ protein. The activation of 5-HT_1B_ induces the activation of ERK, AKT, and the Rho-kinase (ROCK) pathway, which stimulates the translocation of active ERK to the nucleus. As a way to regulate this signaling pathway, the glycogen synthase kinase 3 protein (GSK3) was associated with a possible phosphorylation of the receptor, increasing its inhibitory potential on AC (Figure 2) [12,15].

The 5-HT_1D_ receptor is expressed on the pre- and post-synaptic membrane of neurons in several regions of the nervous system, such as the nigrostriatal pathway, caudate putamen, globus pallidus, and substantia nigra. As autoreceptors, when activated, they inhibit the release of 5-HT from the pre-synaptic terminal through the same mechanism observed for the other 5-HT_1A_ and 5-HT_1B_ serotonergic autoreceptors, changing potassium conductance and calcium influx. Although the mechanisms resulting from the activation of post-synaptic 5-HT_1D_ receptors have not yet been elucidated, it is known that they act through the inhibition of AC, common among 5-HT_1_ receptors and that it is structurally similar to the 5-HT_1B_ receptor [2,17,18]. Likewise, the pharmacological mechanism of action of 5-ht_1e_ and 5-HT_1F_ serotonergic receptors is still unknown. It is believed that 5-ht_1e_ has high affinity for 5-HT and low affinity for 5-HT_2_ agonists, and for this reason, it was classified as 5-HT_1_. As for the 5-HT_1F_ receptor, its expression in terminals and cell bodies of neurons in the human trigeminal ganglion is known. Its activation is associated with AC inhibition, preventing the release of neuropeptides and neurotransmitters, including the calcitonin gene-related peptide (CGRP) and glutamate. This role has been investigated in the planning of migraine treatments, as it has reduced neuronal hypersensitivity and hyperstimulation [19,20].

#### 2.1.1. Treatment of Anxiety 

**Figure 3:**
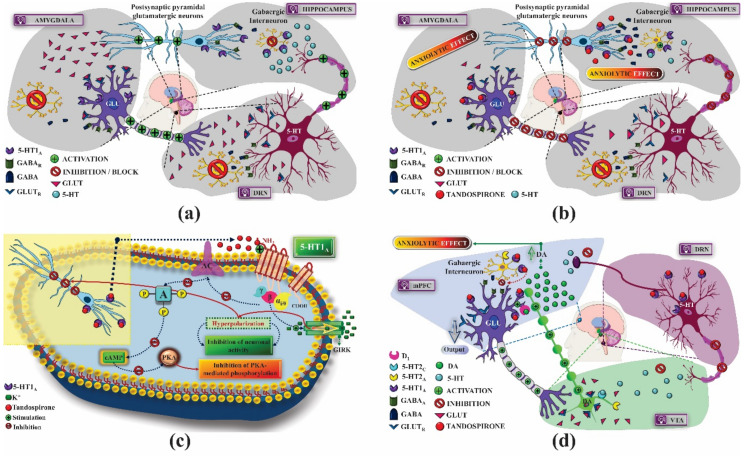
Anxiolytic mechanism of tandospirone. 5-HT_1A_: (**a**) Biological factors have been implicated in the pathogenesis of anxiety. Neurochemical evidence suggests that the dysregulation of serotonin, norepinephrine, gamma-aminobutyric acid (GABA), glutamate, and peptides (corticotropin releasing factor, cholecystokinin, neuropeptide Y) transmission systems are strongly correlated with the pathophysiology of anxiety. In the classic concept of stress, homeostasis is threatened by physical and psychological events, known as stressors. Behavior is aimed at assessing the destabilizing potential of the stressor. If the stressor event does not correspond to any cognitive representation based on previous subjective experiences, there is an increase in alertness, hypervigilance, focused attention, and cognitive processing. The interface between sensory information received and the evaluation process is formed in the limbic region of the brain, which comprises structures such as the hippocampus, the amygdala, and the medial prefrontal cortex (mPFC). The components of the human fear circuit, which can be of particular relevance for destabilization produced by the stressor and, consequently, induction of anxiety, include mPFC (subservient fear extinction processes), the amygdala (risk assessment processing), and the hippocampus. The ascending serotonergic pathway that projects from the DRN into the hippocampus may be implicated in the genesis of behavioral inhibition observed in dangerous situations. During episodes of anxiety crisis, it is postulated that these serotonergic inputs in the hippocampus activate 5-HT_1A_ receptors in GABAergic interneurons. In this case, 5-HT_1A_ receptors coupled to the Gαi/0 protein block adenylate cyclase (AC), prevent the formation of cyclic adenosine monophosphate (cAMP) and reduce the activation of protein kinase A (PKA) which, once reduced, decrease the permeability of calcium (Ca^2+^) to specific channels located in the cell membrane. This reduction in the calcium influx into the GABAergic interneuron culminates in the blocking the exocytosis of the GABA neurotransmitter. The reduction of the hippocampal GABAergic transmission causes an increase in the number of post-synaptic pyramidal glutamatergic neurons fires, stimulating anxiety-related regions such as the amygdala and the DRN, which feeds back and over-stimulates the anxiety-related circuit; (**b**,**c**); tandospirone has a promising anxiolytic effect, which has been shown in animal models, especially of the generalized anxiety disorder (GAD). Tandospirone acts as an anxiolytic by activating the post-synaptic 5-HT_1A_ receptor coupled to the Gαi/0protein, resulting in reduced cAMP formation and PKA inhibition. On the other hand, it activates the G protein-controlled internal rectifying potassium channels (GIRK) by releasing Gβγ subunits, leading to intracellular potassium (K^+^) efflux, hyperpolarization of target neurons and, finally, inhibition of the local neuronal activity [24]; (**d**) another mechanism by which tandospirone exerts its anxiolytic effect is by increasing the release of dopamine (DA) in the VTA. In this case, tandospirone activates the 5-HT_1A_ receptor in DRN or mPFC, directly or indirectly, stimulating dopaminergic transmission in VTA [24].

The stimulation of the pre-synaptic 5-HT_1A_ receptor activity results in an anxiolytic effect by suppressing the release of 5-HT, whose high concentration is associated with the development of anxiety disorders. Based on this mechanism, the 5-HT_1A_ autoreceptor became a target to be explored for the planning of anxiolytic drugs. Examples of these drugs are buspirone and fluoxetine, direct and indirect agonists of this receptor, respectively [21]. Buspirone is a partial agonist of the 5-HT_1A_ autoreceptor, and is used, at low doses, to treat anxiety disorders. High doses of this drug are associated with the opposite clinical effect, the reason has not yet been completely clarified, but it may be related to the affinity of this substance with the 5-HT_1A_ heteroreceptor and the antagonistic activity on pre-synaptic dopamine (DA) D_2_ receptors and adrenergic α_1_ and α_2_ receptors [22]. Another substance, also of the azapirone class and 5-HT_1A_ agonist, is gepirone, which has anxiolytic and antidepressant activity. Gepirone is still in the development phase and has been an alternative to selective serotonin reuptake inhibitors (SSRIs), constituting a possible treatment for anxiety and depression, without causing sexual dysfunction, a common adverse effect among SSRIs [23]. Tandospirone is a post-synaptic 5-HT_1A_ receptor agonist and exerts its anxiolytic activity by inhibiting adenylate cyclase and activating GIRK, hyperpolarizing neurons from regions associated with anxiety disorders, such as the hippocampus and amygdala, where 5-HT_1A_ receptors are expressed, consequently inhibiting local neuronal activity (Figure 3a–c) [24]. In addition, tandospirone appears to facilitate the elimination of fear and reduce anxiety. This effect is mediated by the indirect increase in DA neurotransmission through the dopaminergic loop in the ventral tegmental area (VTA) hippocampus, which improves the synaptic efficacy in the extinction processes in the animal model of post-traumatic stress disorder (PTSD) [25,26] (Figure 3d).

Reference [27] injected the following substances into the dorsal-medial raphe sub-nucleus (dmDR) and neurons in the lateral wings of the raphe dorsal nucleus (lwDR): (1) Kainic acid, an excitatory amino acid; (2) WAY-100635, a 5-HT_1A_ receptor antagonist; and (3) 8-hydroxy-2-(di-n-propylamino) tetraline (8-OH-DPAT), a 5-HT_1A_ receptor agonist. Administration of kainic acid and 8-OH-DPAT reduced anxiety behavior, while administration of the 5-HT_1A_ antagonist produced an anxiogenic effect and accentuated panic symptoms, indicating that the increased anxiety is associated with 5-HT_1A_ inhibitors. To demonstrate the implication of the 5-HT_1A_ receptor as a target for the treatment of anxiety disorders, a study by [28] demonstrated that the activation of serotonergic terminals in the dorsal part of the bed nucleus of the stria terminalis (dBNST) reduces anxiety, while inhibition of these terminals produces anxiogenic effect. In addition, it has been observed that 5-HT induces neuronal hyperpolarization through the 5-HT_1A_ receptor in dBNST, since the administration of an antagonist of these receptors intensifies anxiety-like behavior. Thus, researchers observed that the activation of 5-HT_1A_ is crucial for the anxiolytic effect observed through the activation of the serotonergic terminals in dBNST. An observation about 5-HT_1A_ autoreceptors is their participation in cases of anxiety due to chronic use of SSRIs. Chronic depression treatment with these drugs promotes desensitization of 5-HT_1A_ autoreceptors, preventing them from playing their regulatory role in the serotonergic activity and resulting in anxious behavior induced by neuronal hyperactivity, since 5-HT_1A_ would not be activated to promote hyperpolarization [29].

Although it is not a determining mechanism for the occurrence of anxious symptoms, the activation of serotonergic neurons in the bed nucleus of the stria terminalis (BNST) can be considered a way to modulate the neuronal hyperactivity observed in anxious patients. In this context, [30] investigated the anxiety modulation by means of 5-HT_1A_ heteroreceptors, expressed in the BNST, in male and female rats. The loss of the 5-HT_1A_ receptor in the region did not increase the anxiety behavior, confirming that this is not a determining mechanism for anxiety; however, this loss promoted an increase in fear conditioning in male rats, but not in female rats, which may be associated with the lower neuronal excitability observed in males, increased by the receptor depletion. It has been recently found that the depletion of 5-HT_1A_ heteroreceptor in the dentate gyrus (DG) of the hippocampus by means of the local elevation of glucocorticoid levels; that is, a chronic stress mechanism, is associated with the development of stress-induced anxiety, demonstrating again the importance of the 5-HT_1A_ receptor in the modulation of the neuronal activity [31].

The 5-HT_1B_ receptor is also an important target when it comes to the treatment of mental disorders. To investigate the role of this receptor on anxiety disorders, [32] used a genetic model to inhibit the expression of the 5-HT_1B_ autoreceptor in rats, which resulted in a reduction in behaviors suggestive of anxiety, demonstrated in the open field (OF) test, and decreased depression, observed in forced swimming and sucrose preference tests. These data indicate that blocking 5-HT_1B_ autoreceptors may be an alternative for the development of new treatments for anxiety and depression. Another study investigated the role of agonists in behavioral modulation after cocaine use. In this case, CP 94.253, a 5-HT_1B_ agonist, was administered via intracranial injection in the lateral habenula region, recently related to the neurobiology of anxiety disorders. The agonist attenuated the emergence of anxiogenic behaviors produced by cocaine. The anxiolytic activity was stopped after the administration of the selective 5-HT_1B_ antagonist, NAS-181. These results confirm the contribution of this receptor to the effects resulting from cocaine administration and also reaffirm the possibility of making it a target for treatments against anxiety [33].

Studies on the involvement of serotonergic 5-HT_1D_, 5-ht_1e_, and 5-HT_1F_ receptors as possible targets for treatments against anxiety are recent and scarce. The ACH-000029 compound is undergoing pre-clinical studies, and showed anxiolytic activity, acting through partial activation of 5-HT_1A_ and 5-HT_1D_ receptors, antagonism on 5-HT_2A_, and α_1A_, α_1B_, and α_1D_ adrenergics. The anxiolytic effect was observed in animal models with predictive validity for anxiolytic drugs, such as the marble burying test and the light–dark box. The compound demonstrated regular regions such as amygdala, paraventricular nucleus of the thalamus, retrosplenial cortex, BNST bed, and locus ceruleus, were implicated in the development of anxious symptoms [34]. Vortioxetine is another example of a therapeutic compound that reduces anxiety-like behavior, with the 5-HT_1D_ receptor as a non-specific target. It is an SSRI, but it also acts as an antagonist of the 5-HT_3_, 5-HT_7_, and 5-HT_1D_ receptor, and a partial 5-HT_1B_ and 5-HT_1A_ agonist, reducing the exacerbated expression of the fear memory associated with anxiety and depressive disorders [35].

#### 2.1.2. Treatment of Depression 

**Figure 4:**
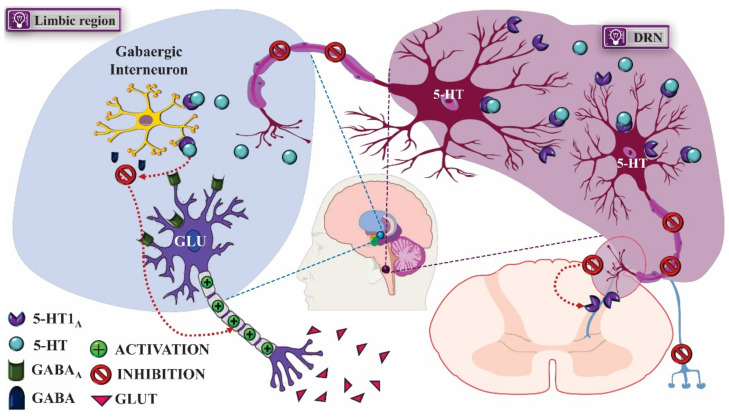
Hypothetical scheme of serotonergic projections resulting from the raphe and innervation of the dorsal horn spinal neurons (DHS) and limbic regions. In the dorsal raphe nucleus (DRN), 5-HT_1A_ receptors activate G protein-controlled internal rectifying potassium channels (GIRK) by releasing Gβγ subunits of the Gα_i/0_ protein, leading to intracellular potassium (K^+^) efflux, hyperpolarization of target neurons, and, finally, inhibition of the local neuronal activity. Hippocampus and cortex heteroreceptors are also coupled to GIRK channels. Thus, activation of both 5-HT_1A_ autoreceptors and the hippocampus and cortex heteroreceptors increases the GIRK current, leading to neuronal hyperpolarization. As shown in the figure, the activation of 5-HT_1A_ autoreceptors reduces the release of serotonin (5-HT) in limbic regions or in DHS. However, the activation of 5-HT_1A_ heteroreceptors located in the DHS reduces the release of local 5-HT, consequently decreasing the release of nociceptive neurotransmitters and reducing pain signals (antinociceptive effect). In limbic regions, the stimulation of these receptors activates GIRK in GABAergic interneurons, hyperpolarizing them and consequently decreasing the influence of GABA on local glutamatergic neurons. As a consequence, there is an increase in the excitatory glutamatergic influence on underlying dopaminergic neurons [37].

The activation of 5-HT_1A_ serotonergic heteroreceptors with 8-OH-DPAT produces antidepressant effect [36]. This activity was associated with the inhibition of GABAergic neurons of the limbic pathway that express 5-HT_1A_, which, once activated, induces neuronal hyperpolarization, inhibiting the release of the γ-aminobutyric acid (GABA), an inhibitory neurotransmitter involved in the development of depression and mood disorders, increasing the glutamatergic influence. This factor is associated with the role of 5-HT_1A_ receptors in the pathogenesis of depression since the activation of 5-HT_1A_ autoreceptors inhibits the release of 5-HT and induces depressive symptoms. These receptors are also expressed in afferent nociceptive fibers in the dorsal horn of the spinal cord (DHS); thus, therapies with 5-HT_1A_ autoreceptor antagonists and heteroreceptor agonists increase the available 5-HT concentration and decrease the release of nociceptive neurotransmitters, enabling not only the treatment of depression, but also chronic pain, often associated with mental disorders (Figure 4) [37,38].

The important role of 5-HT in mood regulation has made the serotonergic system become a determining target for the treatment of depression. In this sense, SSRIs were among the first treatments aimed at increasing the 5-HT concentration; that is, promoting the opposite reaction to that observed in depressive disorders, in which this concentration is reduced. SSRIs act by inhibiting the 5-HT (SERT) transporter, expressed in the pre-synaptic membrane of serotonergic neurons. The ability of these drugs to block this transporter without affecting other neuroreceptors made them stand out from other antidepressant therapies, with fewer adverse and side effects [39]. Vilazodone is an SSRI and partial 5-HT_1A_ receptor agonist and an alternative for the treatment of major depressive disorder (MDD) and generalized anxiety disorder (GAD). Its agonist action on 5-HT_1A_ is possibly responsible for the rapid effect of this substance, different from other SSRIs [40]. Similar to vilazodone, tandospirone is a selective agonist of the 5-HT_1A_ autoreceptor, whose administration, in the short term, promotes a decrease in the release of 5-HT. Chronic tandospirone administration, responsible for its pharmacological outcome, induces the desensitization of these receptors and, consequently, an increase in 5-HT concentration, exhibiting antidepressant activity, with late onset. However, this is not the only way the substance can be used to treat depression. The concomitant administration of tandospirone with SSRI or tricyclic antidepressants (TCAs), is even more effective, enhancing the effect of these drugs and even reducing their adverse effects [24].

Brexpiprazole is a drug used to treat MDD and schizophrenia. This substance acts as an agonist for the following receptors: 5-HT_1A_; dopaminergic D_2_ and D_3_, and as an antagonist of the following receptors: 5-HT_2A_, 5-HT_2B_, 5-HT_7_, and adrenergic α_1A_, α_1B_, α_1D_, and α_2C_, with greater affinity than aripiprazole, which belongs to the same class and with the absence of akathisia, a characteristic adverse effect. The use of brexpiprazole is recommended as an adjunct to antidepressant therapies, where it acts by enhancing the effect of antidepressants such as fluoxetine. This action can be observed in animal models for the assessment of depression and anxiety, such as the forced swimming test and the Vogel conflict test [41,42,43].

5-HT_1B_ antagonists have also been considered adjuvants to antidepressant therapy. Monotherapy with these substances does not induce effective results, which can be explained by the fact that 5-HT_1A_ and 5-HT_1B_ autoreceptors have less distribution and less pronounced activity than SERT. Thus, its application as a therapeutic enhancer is more efficient, intensified through the inhibition of the feedback mechanism that prevents the release of 5-HT [44,45]. Although structurally different, 5-HT_1B_ and 5-HT_1D_ may play similar roles in depressive disorders. Both are hypersensitive in patients diagnosed with these disorders; thus, they are easily activated and inhibit the release of 5-HT, a characteristic factor of the condition. Antidepressant vortioxetine acts by antagonizing 5-HT_1D_, increasing the release of 5-HT [46,47]. As for 5-ht_1e_ and 5-HT_1F_ receptors, there is a lack of studies with antidepressant treatments that target these receptors or that correlate them with the pathogenesis of depression.

## 3. 5-HT_2_ Receptors

### 3.1. Mechanism of Pharmacological Action

**Figure 5:**
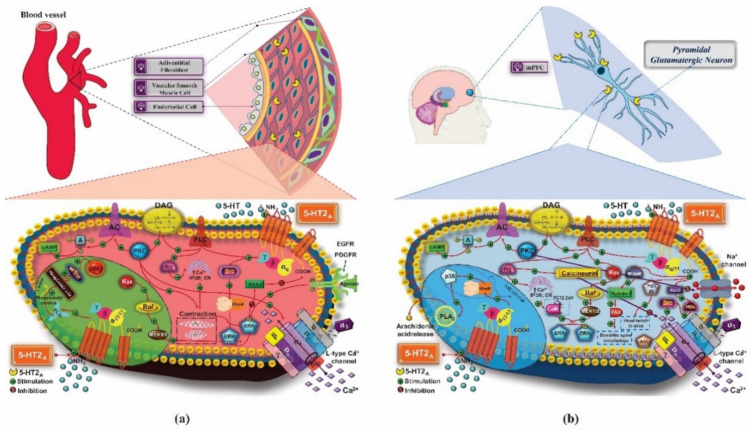
5-HT_2A_ receptor signaling pathways: (**a**) In blood vessels (and other smooth muscles), stimulation of the 5-HT_2A_ by 5-HT receptor activates several signal transduction pathways through the Gα_q/11_ protein (phospholipase C (PLC)/Diacylglycerol (DAG)/protein kinase C (PKC)/calcium (Ca^2+^) and kinase phosphorylation regulated by extracellular signal (ERK), leading to vascular smooth muscle contraction. The “atypical” coupling represented by the green circle demonstrates the specific signaling of the tracheal tissue mediated by the 5-HT_2A_ receptor, corroborating the hypothesis that the activity of these receptors, as well as the integration of their multiple pathways, varies from one tissue to another. In the trachea, activation of the 5-HT_2A_ receptor produces its downstream effects primarily through the mammalian target of rapamycin (mTOR)/p70 ribosomal protein S6 kinase (S6K1) pathway [12]. (**b**) In glutamatergic neurons of the medial prefrontal cortex (mPFC), the coupling of 5-HT to the 5-HT_2A_ receptor stimulates the Gα_q/11_ protein that activates several signal transduction pathways through PLC/DAG/AC/PKC. These, in turn, produce inhibition of Ca^2+^ and Na^+^ conductances. The “atypical” coupling represented by the blue circle demonstrates the activation of phospholipase A2 (PLA_2_) mediated by the 5-HT_2A_ receptor and the subsequent release of arachidonic acid, described as a result of Gα_12/13_-coupled, Rho-mediated, p38 activation in NIH 3T3 cells [48]. Stimulation of PLA_2_ causes the release of arachidonic acid. The pathways of several kinases are involved in the modulation of neuron morphology and plasticity through direct interaction between the C terminal portion of the receptor and specific modulating proteins [12].

5-HT_2_ receptors are coupled to the Gα_q/11_ protein and activate phospholipase C (PLC or PLC-β) and phospholipase A_2_ (PLA_2_). They have three subtypes: 5-HT_2A_, 5-HT_2B_, and 5-HT_2C_. The 5-HT_2A_ receptor is expressed in the cerebral cortex, insula, brain stem, and limbic system. In addition, it mediates contractile responses and regulates the expression of growth factors in these regions. The stimulation of the 5-HT_2A_ receptor promotes the activation of PLC-β, which catalyzes the hydrolysis of phosphatidylinositol-4,5-bisphosphate into inositol 1,4,5-triphosphate (IP3) and diacylglycerol (DAG), increasing the concentration of these messengers in the intracellular medium. IP3 acts by increasing the release of calcium from the endoplasmic reticulum (ER). The high calcium concentration can also promote the activation of voltage-dependent L-type calcium channels, which cause calcium influx, being responsible for the increase of intracellular calcium levels, causing contraction in muscle cells that express this receptor. In addition, DAG induces the activation of protein kinase C (PKC), which favors the activation of AC, formation of cAMP, stimulation of PKA, and activation of calcium channels, increasing the influx of this ion in the cell and, consequently, muscle contraction (Figure 5a).

In pyramidal neurons of mPFC, the 5-HT_2A_ receptor can activate p38, which, coupled to Gα_12/13_, promotes the activation of PLA_2_, inducing the formation of arachidonic acid and stimulating ERK_1_ and ERK_2_ through the activation of Rho kinase. In these neurons, stimulation of 5-HT_2A_ promotes the inhibition of calcium influx in Ca v1.2-type channels. This effect is mediated by the increase in PKC, IP3, and stimulation of calcineurin, which not only reduce the calcium concentration but also inhibit the sodium efflux, a mechanism associated with synaptic plasticity. The activation of the 5-HT_2A_ receptor can also induce the activation of protein kinase MEK (mitogen-activated protein kinase), promoting the activation of ERK. In CNS cells, the 5-HT_2A_ receptor interacts with the postsynaptic density protein 95 (PSD-95), which can regulate the receptor expression. The activation of 5-HT_2A_ also increases the activity of PI3K and Akt (Figure 5b) [12].

5-HT_2A_ receptors can be pre- or post-synaptic. Post-synaptic receptors are part of the mechanism of action of some psychotropic and psychedelic drugs. However, pre-synaptic receptors are a recent discovery, and appear to stimulate glutamatergic transmission and memory consolidation. [49] described three main functions associated with the 5-HT_2A_ pre-synaptic receptor: (a) The potentiation of NMDA glutamatergic receptor responses; (b) involvement in the development of depression; and (c) important participation in associative learning. 5-HT_2A_ receptors have a specific characteristic, the interaction with β-arrestin 1 and 2, known to mediate the desensitization of metabotropic receptors and important for cell signaling performed by 5-HT_2A_ receptors. In addition, these receptors have other mechanisms that regulate their signaling, such as the formation of homo and heteromeric complexes with other metabotropic receptors such as mGlu_2_ and D_2_ [50,51]. The interaction between 5-HT_2A_ and mGlu_2_ can be attested by the synergistic effect between 5-HT_2A_ antagonist antipsychotic drugs and mGlu_2_ agonists, and also by the documented co-localization of these receptors [52,53].

As for 5-HT_2A_, signaling induced by the activation of 5-HT_2B_ receptors occurs differently depending on the tissue and ligand. Stimulation of the 5-HT_2B_ receptor induces cell cycle regulation, with the activation of the platelet-derived growth factor receptor (PDGFR) and the Src family kinase. When stimulated, PDGRF promotes the activation of ERK1 and 2, which has an inducing effect on cyclin D_1_, while the Src family kinase stimulates cyclin E; both cyclins act in the progression from phase G1 of the cell cycle to phase S. This mechanism is associated with the rapid proliferation of cells of some tissues that express the 5-HT_2B_ receptor. In cells in which MAPK was previously depleted, the expression of cyclin D_1_ was reduced, but not of cyclin E, indicating that the MAPK pathway may be involved in this mechanism [54].

**Figure 6:**
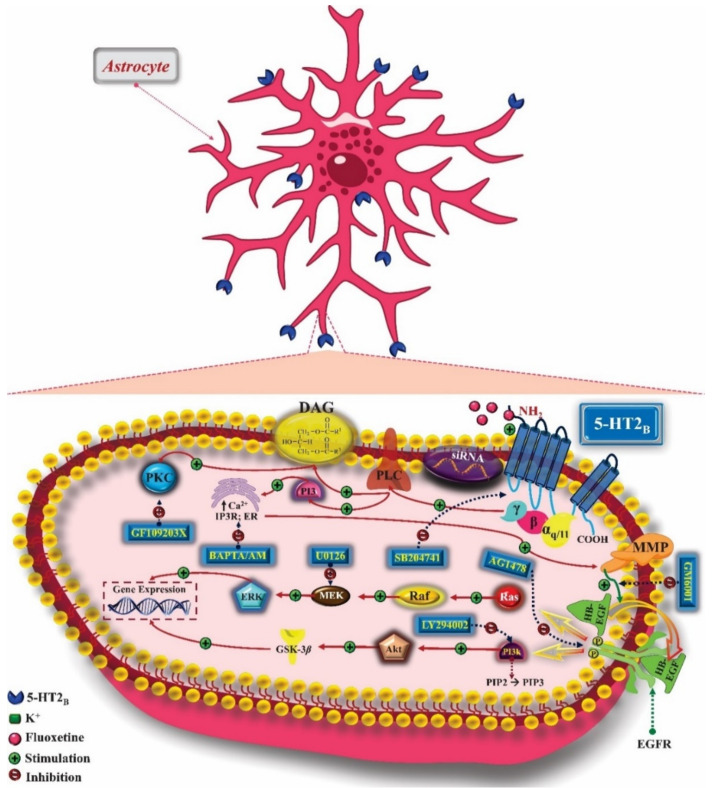
Scheme of pathways that stimulate extracellular signal-regulated kinase (ERK) and phosphorylate protein kinase B (AKT) after stimulation of the 5-HT_2B_ receptor by fluoxetine in astrocytes. Fluoxetine binds and activates 5-HT_2B_ receptors, culminating in the stimulation of Gα_q/11_ protein, which stimulates enzyme phospholipase C (PLC), which catalyzes the hydrolysis of phosphatidylinositol-4,5-bisphosphate in inositol 1,4,5-triphosphate (IP3) and diacylglycerol (DAG). DAG induces protein kinase C (PKC) activity, while IP3 increases the intracellular calcium (Ca^2+^) concentration due to the release of Ca^2+^ from endoplasmic reticulum stocks. Ca^2+^ stimulates zinc-dependent metalloproteinases (MMPs) and leads to the release of growth factors. The released epithelial growth factor receptor (EGFR) ligand stimulates EGFR phosphorylation. ERK, the downstream target of EGFR, is phosphorylated via the Ras/Raf/MEK pathway, and AKT is phosphorylated via the phosphoinositide 3-kinase pathway (PI3K). In addition, PIK3 catalyzes the formation of PIP3 from PIP2. After fluoxetine administration, ERK and AKT phosphorylation was blocked when iRNA against the 5-HT_2B_ receptor was administered or after administration of inhibitors of this receptor (SB204741) (shown in blue), PKC (GF109293X), intracellular Ca^2+^ homeostasis (BAPTA/AM, an intracellular Ca^2+^ chelator), zinc-dependent MMPs (GM6001), EGFR (AG1478), ERK phosphorylation (U0126, a mitogen-activated protein Kinase (MEK) inhibitor), or AKT pathway (LY294002, a PI3K inhibitor) [55].

Previous studies have shown the mechanism resulting from the activation of the 5-HT_2B_ receptor, expressed in astrocytes, by fluoxetine. The activation of 5-HT_2B_ promotes the activation of PKC and the increase of intracellular calcium concentration. This increase in calcium levels induces the activation of metalloproteinases (MMPs), which act by causing the release of growth factors such as the epidermal growth factor receptor (EGFR) ligand that stimulates the phosphorylation of this receptor, which, once phosphorylated, promotes the activation of ERK via the Ras/Raf/MEK signaling pathway and Akt activation via the PI3K pathway. Activation of ERK induces gene expression of cfos and fosB in astrocytes, while AKT inhibits glycogen synthase kinase-3β (GSK-3β), involved not only in metabolic issues, but also in the pathophysiology of mood disorders (Figure 6) [55].

The activation of the 5-HT_2B_ receptor in cardiomyocytes induces the activation of PI3K/AKT and ERK pathways, which can control the expression of nuclear genes that directly influence the permeability of the mitochondrial membrane. The PI3K/Akt pathway inhibits the expression of ANT-1, a cell death regulator, and the ERK pathway inhibits Bax, an apoptosis inducer. A study with mice, whose cardiomyocytes did not have the 5-HT_2B_ receptor, revealed that in these animals, caspase activation occurred, which induced apoptosis and destruction of myofibrils, leading to a dilated cardiopathy condition. On the other hand, mice with excessive expression of these receptors showed intense ANT-1 downregulation, which culminated in increased proliferation and hypertrophic cardiomyopathy. This information suggests that adequate expression of the 5-HT_2B_ receptor promotes a protective effect against heart disease, without inducing uncontrolled proliferation or apoptosis [56]. The activation of the 5-HT_2B_ receptor expressed in transfected cells promotes activation of GTPase and production of IP3. This stimulus also activates the MAPK, PDGRF, PLA_2_, PLC pathway, inducible nitric oxide synthase (iNOS), PI3K and constitutive nitric oxide synthase (cNOS). As a result, activation of ERK 1 and 2, induction of the activity of cyclins D and E, increase in the release of arachidonic acid and production of intracellular cGMP due to the iNOS and cNOS stimulus are observed. PI3K promotes activation of the NF-κB transcription factor [57].

**Figure 7:**
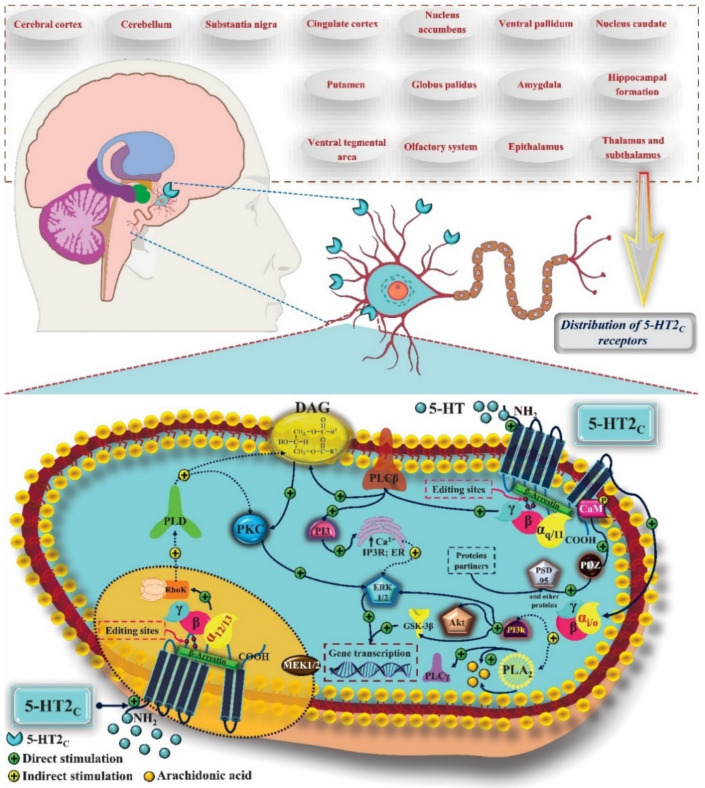
5-HT_2C_ receptor signaling. After activation of the 5-HT_2C_ receptor by 5-HT, the Gα_q/11_ protein is activated and subsequently stimulates phospholipase C (PLC) activity. PLC catalyzes the hydrolysis of phosphatidylinositol-4,5-bisphosphate into inositol 1,4,5-triphosphate (IP3) and diacylglycerol (DAG). IP3 diffuses through the cytoplasm and stimulates the release of calcium (Ca^2+^) from the endoplasmic reticulum and DAG activates protein kinase C (PKC), leading to the phosphorylation of various cellular substrates, which in turn activate several intracellular biochemical cascades. The activation of the 5-HT_2C_ receptor can also stimulate the activity of enzyme phospholipase A_2_ (PLA_2_), which is responsible for the synthesis of arachidonic acid (causing its release in the intracellular space). The “atypical” coupling represented by the orange circle demonstrates that through the coupling of the Gα_12/13_ protein, the activation of the 5-HT_2C_ receptor stimulates phospholipase D (PLD), whose activity converges to PKC activation, which subsequently activates extracellular signal-regulated kinases 1 and 2 (ERK1/2). Another ERK activation pathway is provided through Gα_i/0_, which induces the phosphatidylinositol-3-kinase (PI3K)/protein kinase B (AKT)/glycogen synthase kinase-3β (GSK-3β) cascade. Finally, this signal transduction system controls genetic transcription [58]. The β-Arrestin protein interacts with 5-HT_2C_ receptors and creates a steric impediment that blocks the coupling of heterotrimeric G proteins to the receptor, preventing the activation of Gα proteins.

5-HT_2C_ serotonin receptors are widely distributed throughout the CNS. They have different levels of expression in the cerebral cortex, cerebellum, substantia nigra, cingulate cortex, nucleus accumbens (NAc), ventral pallidum, putamen, caudate nucleus, globus pallidus, amygdala, hippocampal formation, VTA, olfactory system, epithalamus, thalamus, and subthalamus, with greater expression in GABAergic neurons than in glutamatergic neurons. Like the other subtypes, 5-HT_2C_ is coupled to the Gα_q/11_ protein and signals the activation of PLC-β, promoting the mobilization of calcium and increasing its levels in the intracellular environment, in addition to inducing the expression of immediate early genes (IEGs). PLC catalyzes the formation of IP3 and DAG. IP3 causes the release of calcium reserves in the ER, which, associated with the role of CaM, may be related to the activity of some drugs that act on this receptor, such as antidepressants. DAG induces the activation of PKC, which promotes phosphorylation and activation of ERK 1 and 2. Activation of the 5-HT_2C_ receptor can promote the activation of PLA_2_, which leads to the release of arachidonic acid and activation of PLCγ. The Gα_12/13_ protein also participates in the coupling and stimulates the PLD activity, which is involved in the activation of ERK 1 and 2 and the Gβ-γ subunit. Gα_i/0_ can participate in 5-HT_2C_ signaling and induce PLA_2_ and PI3K, whose signaling cascade involves GSK-3β and ERK 1 and 2, culminating in the transcription of the β-Arrestin gene, capable of interacting with the 5-HT_2C_ receptor. As for the other subtypes of 5-HT_2_ receptors, the mechanisms resulting from their activation are different according to tissue and ligand, making it possible to activate only some of these signaling pathways (Figure 7) [58].

#### 3.1.1. Treatment of Anxiety 

The 5-HT_2A_ receptor is involved in behavioral responses and plays an important role in the development of behaviors similar to anxiety and depression. 5-HT_2A_ antagonists can control anxious symptoms, and although the mechanism of their activity is unclear, the role of ERK signaling for this purpose has been suggested [59]. Based on the influence of the intestinal microbiota on immunity and behavior, a study that tested the use of the neonatal prebiotic (BGOS) showed that the administration of lipopolysaccharides (LPS) and induction of acute inflammation in mice promote increased expression of the 5-HT_2A_ receptor. This increase was associated with post-inflammatory anxiety behavior, showed by the submission of these animals to anxiety assessment models [60]. The antagonism of 5-HT_2A_ by ritanserin, ketanserin, R59022, and R59949 inhibits the formation of IP3 and DAG. However, ritanserin and R59022 also exhibit another activity, the specific inhibition of the diacylglycerol kinase alfa enzyme (DGKa), which catalyzes the conversion of DAG into phosphatidic acid (PA) [61]. The analysis of the injection of 5-HT_2A_/5-HT_2C_, DOI agonist in PAG, in hippocampus CA1, CA2, and CA3 subregions, in basolateral amygdala (BLA), and in the lateral LA, demonstrates that the compound produces an anxiolytic effect only with administration in the CA2 subregion. This compound had an opposite effect on the amygdala and PAG compared to that observed in the hippocampus. In the latter, intense anxiolytic activity was observed, while in the others, the effect was anxiogenic [62,63].

In a study using an animal model with zebrafish, 2,5-dimethoxy-4-bromo-amphetamine hydrobromide (DOB), and para-methoxyamphetamine (PMA) showed anxiolytic activity, which was blocked by the administration of the 5-HT_2A_/5-HT_2C_ antagonist, ritanserin, demonstrating that the mechanism of action of DOB and PMA involves these receptors [64]. Analysis conducted by [65] verified the activity of the 5-HT_2A_ agonist, TCB-2, and 5-HT_2A_ antagonist, MDL 11,939, on anxiety and acquisition of conditioned defeat memories in Syrian hamsters. These substances were administered by injection into the nucleus of the BLA. When the same substances were administered to other regions of the brain, they did not produce the same results, indicating a determinant function of the 5-HT_2A_ receptor over local control. These receptors are expressed in pyramidal neurons and GABAergic interneurons in BLA, and their role is to directly or indirectly inhibit the activity of neurons in that region. Thus, the stress-induced downregulation of 5-HT_2A_ receptors increases neuronal excitability and, consequently, fear and anxiety. The administration of the agonist can lead to desensitization of the receptor, which, like downregulation, causes anxiety, while the antagonist avoids desensitization and downregulation, decreasing the anxiety triggered by stress. Although most animal models use rodents, dogs with pathological anxiety were submitted to analysis that verified the 5-HT_2A_ receptor binding index in these animals. A lower binding index and reduced expression of 5-HT_2A_ receptors was observed in these animals, indicating that there is a role for these receptors in the pathophysiology of anxiety disorders, which still needs to be investigated, so that new anxiolytic therapies can be proposed [66].

The administration of the 5-HT_2B_/5-HT_2C_ receptor agonist, mCPP, in PAG, reduced anxiety-like behavior in mice. However, after treatment with ketanserin, a 5-HT_2A_/5-HT_2C_ antagonist, mCPP administration did not produce the same effect, indicating that the 5-HT_2_C receptor plays an important role in modulating anxiety in PAG, since its activation induced an anxiolytic effect [67]. There are few studies that describe the 5-HT_2B_ receptor as a possible target for the treatment of anxiety, and some previous analyses indicate the anxiolytic activity of 5-HT_2B_ agonists, such as BW723C86, which demonstrated this effect in the Vogel conflict and Geller–Seifter test [68] (tests based on associative learning), but was ineffective in the elevated plus-maze (EPM) test [69,70] (ethologically-based model).

There are disagreements about the role of the 5-HT_2C_ receptor in anxiety disorders, since the results of its activation or blockage are different according to the CNS region submitted to analysis. Recent studies have shown that the administration of the 5-HT_2B_/5-HT_2C_ agonist in the dorsal hippocampus (DH) of rats induced anxiety, but the administration of selective 5-HT_2C_ agonists, MK-212 and RO-600175, promoted an anxiolytic effect, indicating that local activation of 5-HT_2C_ causes the opposite effect of local activation of 5-HT_2B_. As expected, the 5-HT_2C_ antagonist, SB-242084, demonstrated anxiogenic activity. These results were observed in the Vogel conflict, elevated T maze, and in the light–dark box tests. This study also verified the influence of the 5-HT_2C_ receptor on the activity of the tricyclic imipramine antidepressant, and it was then observed that the blockade of 5-HT_2C_ by SB-242084 in DH did not alter its anxiolytic effect [71].

This 5-HT_2C_ receptor is also involved in the development of alcohol withdrawal anxiety. In this context, increase in neuronal excitability and negative regulation of type M potassium channels are observed in LHb, a region known for its role in the pathophysiology of anxiety disorders. Stimulation of the 5-HT_2C_ receptor in LHb, with selective WAY161503 agonist, contributed to the negative regulation of M channels and induced anxious symptoms, while blocking the receptor with SB242084 attenuated these symptoms [72].

Agomelatine, a selective agonist of melatonergic MT_1_ and MT_2_ receptors, 5-HT_2C_ antagonist, acts by modulating the GABAergic pathway and modulating monoamine levels in depressed patients. Its application for the treatment of GAD is recommended by some studies, whose clinical tests indicate improvement of the condition and safety in use. The administration of agomelatine promotes the regulation of plasma levels of vasopressin in men and women and oxytocin in women, which are altered in animals with behavior similar to anxiety [73,74]. Desensitization of the 5-HT_2C_ receptor in the cerebral cortex is capable of attenuating anxious behavior; thus, its activation can promote this behavior. To block this effect, 5-HT_2C_ antagonists such as SB-242084 are proposed, which had an acute effect similar to that of SSRI on the electroencephalogram (EEG), providing further evidence of the therapeutic potential of this compound [75].

Although there are studies describing that the use of 5-HT_2C_ antagonists produces an anxiolytic effect, male 5-HT_2C_ knockout mice exhibit behavior similar to anxiety. In an attempt to explain these observations, a study analyzed the levels of c-Fos, a neuronal activation marker, in 5-HT_2C_ knockout mice. The study reported high levels of this marker in the BNST and in the central nucleus of the amygdala (CeA), regions with high density of neurons secreting the corticotrophin-releasing hormone (CRH), which means that the high secretion of this hormone is also associated with intensification of the anxious behavior. These data may support the development of new studies and therapies based on the important role of the 5-HT_2C_ receptor in modulating anxiety [76].

#### 3.1.2. Treatment of Depression

5-HT_2_ serotonergic receptors have been associated with the pathophysiology of a variety of mental disorders, including depression. Investigation of the binding rate and activation of 5-HT_2A_, 5-HT_1A_, and SERT receptors in the auditory cortex of patients with MDD, compared to a control group, demonstrates that patients with MDD do not present changes in the binding rate of 5-HT_1A_ and SERT receptors, but there is a significant reduction in the binding rate to the 5-HT_2A_ receptor [77]. Nefazodone is an antidepressant that acts as a postsynaptic 5-HT_2A_ receptor antagonist. The use of nefazodone has significant efficacy for clinical conditions consistent with the depressive state, including acute non-psychotic bipolar depression from moderate to severe intensity [78]. Despite not having adverse effects common to other antidepressants, such as sexual dysfunction and intestinal constipation, nefazodone induces hepatotoxicity by compromising the mitochondrial function of hepatocytes [79]. Currently, this medication is indicated only for cases in which the patient is refractory to other antidepressants. In addition to blocking 5-HT_2A_, nefazodone also produces a moderate inhibition of 5-HT and norepinephrine (NE) reuptake and can interact with other drugs, as it inhibits the CYP3A4 isoenzyme of the P450 cytochrome microsomal system located in hepatocytes [80,81].

Mirtazapine is also an antidepressant that acts on 5-HT_2A_ receptors. Its mechanism of action includes antagonism of pre- and post-synaptic α_2_ adrenergic receptors and antagonism of 5-HT_2C_ and 5-HT_3_ receptors. Blocking the pre-synaptic α_2_ autoreceptors promotes an increase in the available NE concentration, as well as antagonism of 5-HT_2C_ and 5-HT_3_ serotonergic receptors, and induces an increase in 5-HT (Figure S1a,b (Supplementary Material)) [82]. In cases of individuals who do not respond to other antidepressants, or when there is depression in comorbidity with other mental diseases, it has been proposed that mirtazapine would be associated with other drugs, such as SSRIs or selective 5-HT and NE reuptake inhibitors (SNRIs). A phase III clinical study tested this hypothesis with SSRI and SNRI for MDD; however, the results do not indicate a benefit for the association, but considerable adverse effects were observed [83]. In addition to the possibility of antidepressant treatments with substances that act on 5-HT_2C_ receptors, and the potentiation mechanism of other antidepressants, there is evidence that ritanserin, a 5-HT_2A_/5-HT_2C_ antagonist, significantly reduces extrapyramidal side effects of other antidepressants [84].

The administration of DOI, a 5-HT_2A_/5-HT_2C_ agonist, in the orbitofrontal cortex (OFC), induces the following responses: (1) Depression-like behaviors; (2) reduced expression of Kalirin-7 (Kal7), an essential component of excitatory synapses; and (3) reduced expression of PSD-95, a protein capable of modulating 5-HT_2A_ receptor expression. In addition, reduction in the density of dendritic spines was observed. This information corroborates the statement that the 5-HT_2A_ receptor plays an important role in the synaptic plasticity and neurobiology of depression, as well as its interaction with PSD-95. The administration of ketanserin, a 5-HT_2A_ antagonist, reverses these effects, reinforcing information about its performance [85,86].

The 5-HT_2B_ receptor expressed in astrocytes is involved in the development of depression in mice with Parkinson’s disease induced by the administration of 1-methyl-4-phenyl-1,2,3,6-tetrahydropyridine (MPTP). MPTP treatment causes downregulation of 5-HT_2B_ receptors, while fluoxetine promotes the upregulation of these receptors and improvement of the depressive state [87]. An analysis of the interaction between SSRIs and the 5-HT_2B_ receptor in astrocytes shows the possible mechanism of SSRI action on glial cells, which do not express SERT, the main target of these substances. SSRIs have dose-dependent affinity and promote the activation of ERK 1 and 2 and PLA_2_, mediated by 5-HT_2B_. Paroxetine is an SSRI antidepressant, the administration of which caused the activation of the astrocytic 5-HT_2B_ receptor, responsible for the signaling that activates MMP, PLA_2_, EGFR, and ERK. Like paroxetine, sertraline, fluvoxamine, fluoxetine, and citalopram are also SSRIs used in the treatment of depression. These drugs acted similarly on the astrocytic 5-HT_2B_ receptor, inducing the same signaling pathway. However, the therapeutic effect occurred about 3 weeks after starting treatment, indicating that acute administration has no significant efficacy [88,89].

Reference [90] conducted a study that investigated the role of 5-HT_2B_ receptors in depression and the SSRI activity. 5-HT_2B_ knockout mice showed depressive symptoms after 4 weeks of isolation. SSRI administration did not alleviate the symptoms, demonstrating that 5-HT_2B_ is a considerable target for depression therapy. In addition to these data, stimulating 5-HT_2B_ expression has been shown to have antidepressant activity. Treatment with fluoxetine in association with leptin causes an increase in the expression of these receptors in astrocytes, and this increase is able to mitigate the symptoms of MDD associated with sleep deprivation. The effects of this treatment demonstrate one more possibility of modulating these receptors to treat depression through monotherapy with 5-HT_2B_ agonists or combined therapy [91].

As for the 5-HT_2C_ receptor, studies indicate the possibility for the treatment of obesity and disorders associated with the consumption of psychostimulant drugs through the activation of this receptor, and for the treatment of anxiety, depression, and schizophrenia using 5-HT_2C_ antagonists. The development of positive allosteric modulators (PAM) allows the modulation of the affinity and efficacy of the ligand of this receptor. PNU-69176E is a selective PAM for 5-HT_2C_ and its application increases the release of calcium in the intracellular medium, which is stimulated by the activation of the receptor [92]. Agomelatine is a 5-HT_2C_ antidepressant antagonist and agonist of melatonin receptors M_1_ and M_2_. The existence of a cluster between M2 and 5-HT_2C_ receptors has been proposed, which would explain the higher Gα_i/0_/Gα_q/11_ activation ratio promoted by agomelatine, compared to melatonin and 5-HT. In addition, the regulation of the circadian rhythm associated with the role of melatonin is involved in controlling mood and intensity of depressive symptoms [86,93,94]. Selective antagonists of the 5-HT_2C_ receptor, SB242084 and RS 102221, are capable of enhancing the action of SSRIs, as well as ketanserin, a non-selective antagonist of this receptor. This action is observed by the increase in the levels of available serotonin, the increase of which is even more intense than that observed only with the administration of SSRI alone [95]. The intensification of the effect of citalopram on the levels of 5-HT and DA in ATV and NAc by SB242084 is another evidence of the enhancement of SSRIs by 5-HT_2C_ receptor antagonists. This association not only promoted an increase in 5-HT and DA levels, but also optimized the onset of action of citalopram. This effect was associated with the inhibition of tonic DA release induced by signaling the 5-HT_2C_ receptor blocked by SB242084 [96].

Mirtazapine is an atypical antidepressant that acts as an antagonist of 5-HT_2C_, 5-HT_2A_, and 5-HT_3_ receptors; post-synaptic α_2_ adrenergic; and histaminergic H_1_. This action increases the levels of 5-HT and NE. This high concentration of available 5-HT interacts with the 5-HT_1A_ receptor, whose activation promotes a reduction in depressive symptoms. Thus, the antidepressant effect of mirtazapine occurs indirectly. In addition, by blocking the 5-HT_2C_ receptor, mirtazapine differs from other antidepressants that have adverse effects such as akathisia and sexual dysfunction [97,98]. Substance S32006 is a potent 5-HT_2C_/5-HT_2B_ antagonist that exhibited antidepressant activity in the forced swimming and marble burying tests and showed anxiolytic activity, observed in the Vogel conflict test [99].

Compound S32212 is an inverse agonist of the 5-HT_2C_ receptor, which makes it different from other substances that act on this receptor and have antidepressant activity. S32212 is also a α_2_ adrenergic receptor antagonist and although the mechanisms related to its activity have not yet been clarified, its administration induced an antidepressant effect, demonstrated in the forced swim and preference for sucrose tests. In addition, an increase in DA and acetylcholine (Ach) levels and anxiolytic activity was observed in the Vogel conflict test [100]. The 5-HT_2C_ receptor agonist, WAY-163909, exhibited rapid onset antidepressant activity, with similar behavior to that promoted by SSRI in the forced swimming test and also in the resident-intruder and olfactory bulbectomy models. This activity was inhibited by the administration of the selective 5-HT_2C_ receptor antagonist SB242084 [101]. These studies serve as a basis for the development of new research that explores the potential of 5-HT_2C_ receptors as a target for the treatment of depression and as an alternative to joint therapy with other antidepressants.

## 4. 5-HT_3_ Receptors

### 4.1. Mechanism of Pharmacological Action

5-HT_3_ receptors are coupled to ion channels composed of five subunits: 5-HT_3A_, 5-HT_3B_, 5-HT_3C_, 5-HT_3D_, and 5-HT_3E_. These receptors are expressed in the CNS and peripheral nervous system (PNS), including the enteric system. All receptors have the 5-HT_3A_ subunit, but the other subunits are not always expressed [102]. The receptor was characterized in the homomeric form, presenting only the 5-HT_3A_ subunit and in the heteromeric form with 5-HT_3A_ and 5-HT_3B_. 5-HT_3B_ and 5-HT_3C_ subunits are not capable of forming a homomeric receptor, and 5-HT_3D_ and 5-HT_3E_ subunits have not yet been characterized in terms of their expression and function. Homomeric 5-HT_3A_ and heteromeric 5-HT_3A_ and 5-HT_3B_ forms are found in peripheral and central neurons, mononuclear cells, lymphocytes, and enterochromaffin cells. In the CNS, the 5-HT_3_ receptor is involved in integration processes of the vomiting reflex, pain processing, reward, and anxiety systems. This receptor can be pre- or post-synaptic. The activation of the pre-synaptic 5-HT_3_ receptor, accompanied by neuronal depolarization, promotes calcium influx and the mobilization of intracellular calcium reserves, causing the exocytosis of the neurotransmitter. Activation of the post-synaptic receptor promotes sodium influx, inducing depolarization (Figure S2 (Supplementary Material)). 5-HT_3A_ and 5-HT_3B_ heteromeric receptors have low permeability to calcium ions and less sensitivity to 5-HT, unlike homomeric 5-HT_3A_ receptors, allowing the distinction between them [103].

The location of the 5-HT_3_ receptor defines its effect and the activation of this receptor in raphe neurons causes the release of 5-HT, which acts on 5-HT_1A_ autoreceptors in the DRN, exerting inhibitory activity and reducing the neuronal firing rate. The 5-HT_3_ receptor is expressed in GABAergic interneurons of the hippocampus and PFC. The activation of these receptors in the hippocampus causes the depolarization of local GABAergic neurons and release of GABA, an inhibitory effect. In PFC, the 5-HT_3_ receptor is mainly expressed in GABAergic neurons. Increasing the serotonin concentration activates 5-HT_3_ receptors, but in the long run, these receptors are desensitized, and 5-HT activates the 5-HT_2A_ receptor, with excitatory activity. In the striatum and NAc, 5-HT_3_ activation promotes an increase in the DA release, an effect blocked by antagonists, which reduces nerve activity, a mechanism for controlling mood disorders, such as anxiety [104].

#### 4.1.1. Treatment of Anxiety 

Activation of the 5-HT_3_ receptor induces anxious symptoms, but blocking this post-synaptic receptor in the hippocampus and NAc produces anxiolytic effect [103]. Compound (4-benzylpiperazine-1-yl)-(3-methoxyquinoxaline-2-yl) methanone is a 5-HT_3_ antagonist, whose anxiolytic activity was tested in the EPM, light–dark box, and OF test, using mice. The administration of the compound together with fluoxetine reduced anxiety symptoms induced by LPS in all tested models. Increase in serotonin levels was also observed, which could intensify such symptoms; however, it was possibly prevented by the action of the 5-HT_3_ antagonist [105]. 5-HT_3_ receptor antagonists have an advantage over benzodiazepines for the treatment of anxiety, as they do not present sedative or hypnotic activity and there is no evidence of dependence. These data suggest that the 5-HT_3_ receptor blockade does not stimulate GABAergic activity; on the contrary, this blockage reduces the release of GABA, indicating that its anxiolytic action does not occur by mechanisms that activate GABA signaling [106]. The 5-HT_3_ antagonist, N-n-propyl-3-ethoxyquinoxaline-2-carboxamide (6n), also decreased anxiety in rats, whose behavior was observed in the marble burying test, EPM, sucrose preference test, and OF test [107].

Blocking the 5-HT_3_ receptor in the amygdala and DRN reduces anxiety, as does the downregulation of that receptor in the hypothalamus, LA, CeA, and BNST. Tropisetron is a 5-HT_3_ receptor antagonist, which has an anxiolytic effect and reduces NO and iNOS levels in pathological conditions. These two effects can be correlated since mitochondrial dysfunction and high concentrations of reactive oxygen species can be stress products and are associated with the pathophysiology of anxiety and mood disorders. Thus, the anxiolytic activity of tropisetron may be associated with improved mitochondrial function [108]. In addition, neuroinflammation is an immune mechanism that participates in the pathogenesis of anxiety disorders. Tropisetron has been shown to inhibit the release of pro-inflammatory cytokines and chemokines and LPS-induced microglia proliferation. Tropisetron, like most 5-HT_3_ antagonists, reduces the release of substance P, whose interaction with NK1 receptors favors the inflammatory response in the CNS, nuclear NF-κB translocation and stimulates the production of cytokines by the microglia. All of these events are inhibited after tropisetron administration (Figure S3 (Supplementary Material)) [109].

Through mechanisms not yet elucidated, 5-HT_3_ antagonists alosetron [110], ondansetron [111], and zacopride [112] demonstrated anxiolytic activity in animal models of anxiety assessment. N-cyclohexyl-3-methoxyquinoxaline-2-carboxamide (QCM-13) is a 5-HT_3_ antagonist that showed potent anxiolytic activity in the light–dark box, EPM and OF tests. This activity was related to the increased availability of serotonin after blocking 5-HT_3_ receptors; they are possibly receptors located in GABAergic interneurons, since this blockade would cause disinhibition of underlying serotonergic neurons. In addition, the 5-HT_3_ post-synaptic receptor can be expressed in adrenergic, GABAergic, and dopaminergic neurons, whose neurotransmission modulation can promote anxiolytic effects. Similarly, other compounds such as N-(3-chloro-2-methylphenyl)-quinoxaline-2-carboxamide (4i) [113] act by antagonizing 5-HT_3_ receptors and as a result, attenuate anxiety-like behavior. The location of the 5-HT_3_ receptor is crucial for its function, and the antagonism of this receptor in the amygdala and DRN causes a decrease in local neuronal activity and reduces anxious symptoms [106]. Blocking this receptor is a significant pharmacological strategy with few reported adverse effects. Studies that investigate the efficacy and safety of therapeutic agents, 5-HT_3_ antagonists, for the treatment of anxiety, are innovative and should be expanded.

#### 4.1.2. Treatment of Depression 

Some antidepressants widely used in clinical practice have an affinity for the 5-HT_3_ receptor, such as fluoxetine, which can interact with 5-HT_3_ antagonists and have their antidepressant activity enhanced. Granisetron is an antagonist of this receptor and its activity can alleviate gastrointestinal disorders associated with the use of SSRIs. At low doses, an increase in the antidepressant action of fluoxetine is also observed when administered in conjunction with granisetron [114]. A previous study has shown that other antidepressants such as imipramine, phenelzine, and iproniazid are able to inhibit the serotonergic current mediated by the 5-HT_3_ receptor, blocking its action. This effect has not been elucidated so far, but 5-HT_3_ receptor antagonism is constantly associated not only with gastroprotective activity, but also with antidepressant action [115].

Ondansetron is a 5-HT_3_ antagonist, which, administered in combination with the SSRI paroxetine, enhanced its antidepressant activity. The authors of this study propose that this effect occurred through the inhibition of 5-HT_3_ receptors in hippocampal GABAergic interneurons, which, when activated, act to inhibit the release of 5-HT by serotonergic neurons. In this case, the blockade caused by ondansetron increases the 5-HT concentration, enhancing the SSRI action [116]. Another study revealed that ondansetron inhibits the depressive and anxious phenotype in diabetic mice by blocking the 5-HT_3_ receptor. Mice showed high levels of 5-HT and intense oxidative stress, which was attenuated by treatment with ondansetron, which increased the expression of antioxidant factors, such as glutathione (GSH), and increased levels of 5-HT, producing results comparable to fluoxetine, a widely used antidepressant [111,117].

Compound N-(benzo [d] thiazol-2-yl)-3-methoxyquinoxaline-2-carboxamide is a potent 5-HT_3_ receptor antagonist. This substance reduced the resignation that is considered a depressive-like behavior in mice. The chronic administration of this compound reduced the depressive behavior and oxidative stress induced by the mild and unpredictable chronic stress protocol, with an increase in the activity of antioxidant enzymes and reduction in the levels of corticosterone. Thus, the antidepressant action may occur due to its antioxidant and regulatory activity of the hypothalamic–pituitary–adrenal (HPA) axis [118].

The val66met genetic polymorphism in the pro-domain of the BDNF gene causes reduction in the secretion of the brain-derived neurotrophic factor (BDNF); the subsequent mechanisms are still unknown. However, in mice with this profile, downregulation of 5-HT_3A_ homomeric receptors and upregulation of 5-HT_2C_ receptors were observed, which inhibited long-term depression in the hippocampus, increasing synaptic activity. This effect was blocked by the administration of a 5-HT_3A_ agonist [119].

HBK-14 and HBK-15 are 5-HT_3_, 5-HT_1A_ and 5-HT_7_ receptor antagonists. The administration of HBK-15 in combination with fluoxetine or ketamine resulted in antidepressant and anxiolytic activity in mice with corticosterone-induced depression. This combination also prevented a decrease in the levels of neural growth factor (NGF) and BDNF, whose levels tend to be reduced in individuals with mood disorders. In turn, HBK-14 showed anxiolytic activity in this model. Mice that participated in the analysis were submitted to the sucrose preference test, forced swimming test, and EPM. These results have advantages for the association of antidepressants with 5-HT_3_ antagonists, which have been shown to be able to intensify their antidepressant action [120].

A study described some pathological pathways common to obesity and depression, such as hyperactivity of the HPA axis, oxidative imbalance, increased levels of inflammatory mediators, leptin, and insulin resistance, reduced BDNF levels and serotonergic signaling dysregulation. These common factors are influenced by serotonergic signaling and explain the fact that 5-HT_3_ receptor antagonists reduce appetite and depressive behavior through mechanisms that involve modulating the HPA axis, reducing inflammatory cytokines and increasing BDNF levels [121].

Vortioxetine is an antidepressant that acts as a 5-HT_3_, 5-HT_7_, and 5-HT_1D_ receptor antagonist, partial 5-HT_1B_ agonist, 5-HT_1A_ agonist, and SERT inhibitor. In [122], the authors presented a hypothesis for the involvement of 5-HT_3_ receptors in the antidepressant activity of vortioxetine. According to the study, this antidepressant promotes an increase in monoamine levels, greater than that promoted by SSRIs. The antagonism of 5-HT_3_ receptors in GABAergic interneurons blocks the inhibitory effect of these neurons on glutamatergic and monoaminergic neurons, increasing neurotransmission in the forebrain, which results in a decrease in depressive symptoms. A double-blind study of patients with MDD compared the results of administering vortioxetine with placebo. Treatment with vortioxetine for two weeks caused significant improvement in the clinical condition, with an onset of action observed in the first 24 h [123].

Tropisetron has been shown to be able to attenuate depressive behavior in mice submitted to juvenile social isolation stress. This effect is mediated by inhibiting iNOS activity and reducing oxidative stress, which is associated with the depressive phenotype. This activity was confirmed after aminoguanidine administration, a specific iNOS inhibitor that enhances the action of tropisetron [124]. This suggests that the mechanism of action of tropisetron may be correlated with the nitrergic system and the pathophysiology of mood disorders.

Some piperazine analogs of naphthyridine-3-carboxamides and indole-2-carboxamides are known to act as 5-HT_3_ receptor antagonists and have demonstrated antidepressant activity in the forced swim test [125]. Other compounds that showed similar antidepressant activity in animal models for assessing depressive disorders are: N-(3-chloro-2-methylphenyl)-quinoxaline-2-carboxamide (4i) [113], 3-methoxy-Np-tolylquinoxaline-2-carboxamide (QCM-4) [126], Nn-propyl-3-ethoxyquinoxaline-2-carboxamide (6n) [107], and (4-phenylpiperazin-1-yl) (quinoxalin-2-yl) methanone (4a ) [127].

## 5. 5-HT_4_ Receptors

### 5.1. Mechanism of Pharmacological Action

The 5-HT_4_ receptor can be found in central areas such as the limbic system, basal ganglia, olfactory tubercle, hippocampus, pre-Bötzinger complex, and in the periphery, in the gastrointestinal tract, urinary bladder, myocardium, and adrenal glands. It has eight isoforms identified so far, which differ from each other by their carbonic terminals: 5-HT_4A_, 5-HT_4B_, 5-HT_4C_, 5-HT_4D_, 5-HT_4E_, 5-HT_4F_, 5-HT_4G_, and 5-HT_4H_. The 5-HT_4_ receptor is coupled to the G protein through which it stimulates the activity of AC and PKA, increasing the cAMP concentration. The 5-HT_4_ (b) isoform is capable of inhibiting the AC activity through its interaction with the Gα_i/0_ protein and the 5-HT_4A_ isoform interacts with the Gα_13_ protein, activating the Ras homolog family member A (RhoA), which stimulates the actin expression and activates the serum response factor (SRF) complex, which regulates the gene expression of factors that participate in processes of cell proliferation, differentiation and development. The interaction with Gα_13_ also activates PKA, independently of cAMP, through the A-kinase anchoring protein (AKAP110) (Figure S4 (Supplementary Material)) [128,129].

The activation of this receptor can trigger different mechanisms, according to the cell type in which it is expressed. The activation of 5-HT_4_ in cholinergic neurons, followed by increase in cAMP, is a mechanism associated with neurodegenerative diseases, such as Alzheimer’s disease. In this case, the elevation of cAMP levels causes activation of the CREB and BDNF expression, which acts in the formation of memory. The activation of 5-HT_4_ in neurons also induces the blockage of potassium channels and mobilization of intracellular calcium, causing the release of the neurotransmitter in the synaptic slit [130]. When expressed in enterocytes and enteroendocrine cells, activation of 5-HT_4_ and increase of cAMP favor intestinal motility and secretion of fluid and mucus [131].

#### 5.1.1. Treatment of Anxiety 

The activation of the 5-HT_4_ receptor can promote a response similar to that produced by treatment with fluoxetine. RS67333 is a partial 5-HT_4_ agonist, which exhibited anxiolytic activity with the onset of action faster than fluoxetine. The activation of the 5-HT_4_ receptor by RS67333 caused the release of 5-HT in the DRN, which activates 5-HT_1A_ receptors, reducing neuronal hyperactivation and anxious symptoms. RS67333 also stimulated neurogenesis; that is, neuronal proliferation and maturation. The anxiolytic activity of this substance was observed through EPM and OF tests [132]. Prevention of psychiatric diseases, such as mood disorders, can occur by increasing the ability to tolerate stressful events. Stressful stimuli can promote anxiety disorders. In [133], the authors tested the effectiveness of prophylaxis with 5-HT_4_ agonists against anxiety and depression. RS67333 was effective in prophylaxis against anxiety, but not against depression. Prucalopride, a selective high-affinity 5-HT_4_ agonist, and PF-04995274, a partial agonist of this receptor, prevented depressive behavior, but not anxiety. The neurobiological mechanisms that can explain the difference in action of these compounds have not yet been clarified.

A study investigated the effect of administering RS 39604, a 5-HT_4_ receptor antagonist, and RS67333. The substances were administered to rats submitted to elevated zero maze (EZM), an animal model for assessing anxiety. As expected, the 5-HT_4_ agonist RS67333 attenuated the anxious behavior, as well as the 5-HT_4_ antagonist, which, paradoxically, showed anxiolytic activity. The authors suggested that the 5-HT_4_ receptor does not have a direct relationship with the pathophysiology of anxiety disorders. The performance of new comparative tests using other animal models could confirm or refute these results. However, agonists and antagonists have been tested in isolation in other studies, showing similar results; that is, both antagonism and activation of the 5-HT_4_ receptor cause anxiolytic effect [134]. An example is the result of a study that analyzed the effect of two selective 5-HT_4_ receptor antagonists, SB204070 and GR113808. Both compounds showed dose-dependent anxiolytic activity. The animals that participated in the study were evaluated in the EPM test [135].

The anxiolytic effect of RS67333 can occur through the activation of 5-HT_4_ in mPFC receptors and in the DRN. This activation is accompanied by the inhibition of cortical glutamatergic neurons in the DRN, which may have been induced through the activation of GABAergic neurons present in this region. These neurons are able to modulate the activity of other glutamatergic cells through the inhibitory effect of GABA [136]. Reference [137] suggested that the 5-HT_4_ receptor is important for the anxiolytic action of SSRIs, such as fluoxetine. To test this hypothesis, these authors observed the activity of fluoxetine on knockout mice for the 5-HT_4_ receptor. In mice that express this receptor, fluoxetine induces reduction in BDNF expression, but in knockout mice, this effect did not occur, and the anxiolytic activity was not observed in this case. This information indicates that the 5-HT_4_ receptor is essential for the anxiolytic action of fluoxetine and that the role of 5-HT_4_ on the BDNF expression is a point that should be explored to explain this relationship. Research with compounds that act on the 5-HT_4_ receptor to promote the anxiolytic effect are still inconsistent and with unknown mechanism of action and safety profile, impairing the development of new anxiolytic treatments that target this receptor.

#### 5.1.2. Treatment of Depression 

5-HT_4_ receptors are involved in the pathophysiology of depression and their participation can be modulated by casein kinase 2 (CK2), which acts by phosphorylating specific transcriptional regulators of the brain. CK2α knockout mice have an antidepressant phenotype. The upregulation of 5-HT_4_ receptors in PFC was observed in these animals. The overexpression of this receptor, and the consequent increase in signaling, may be responsible for the phenotype exhibited in these animals [138]. 5-HT_4_ receptors participate in the therapeutic response to SSRIs, as observed after fluoxetine administration to 5-HT_4_ knockout mice, in which fluoxetine did not exhibit antidepressant or anxiolytic activity. Similarly, 5-HT_4_ receptor antagonism also prevents fluoxetine action. In this sense, tests with 5-HT_4_ agonists could show positive results, since their antagonism or lack of expression causes the opposite effect [139].

The administration of the 5-HT_4_ agonist RS67333 for three days was able to attenuate the immobility behavior of mice in the forced swim test. In addition, some brain parameters that are modified after chronic treatment with other antidepressants have been observed after three days of treatment with RS67333, such as desensitization of 5-HT_1A_ autoreceptors, increased tonus of 5-HT_1A_ heteroreceptors in the hippocampus, and increased phosphorylation of CREB and hippocampal neurogenesis. To improve this comparison, SSRI citalopram was administered for three days; however, no effect was observed. 5-HT_4_ activation demonstrated not only its effectiveness on depressive symptoms, but also faster action compared to the most common antidepressants currently commercialized [140].

Another study that also analyzed the antidepressant potential of RS67333 reported the same effects described above after treatment for three days and described the results of the administration of this 5-HT_4_ agonist for seven days. In this case, AC desensitization and a more significant increase in BDNF, CREB, AKT, and hippocampal neurogenesis were observed. The complete reversal of the anhedonic state, characteristic of depression, was also observed [141]. Although there are not many studies that clarify the mechanism of action, effectiveness of 5-HT_4_ agonists as antidepressants or therapeutic coadjuvants and the investigation of the pharmacological potential of these compounds have attracted the attention of researchers in the area. The proposal for an antidepressant treatment with the association of SSRI and 5-HT_4_ agonist showed positive results, enhancing the action of the agonist. The combined treatment with citalopram and RS67333 intensified the phosphorylation capacity of CREB in the hippocampus and increased tonus of 5-HT_1A_ hippocampal heteroreceptors. Therefore, the combination of RS67333 with fluvoxamine, citalopram, or fluoxetine showed better results in the forced swim test, compared to monotherapy with these substances alone [142].

## 6. 5-HT_5_ Receptors

### 6.1. Mechanism of Pharmacological Action

The 5-HT_5A_ serotonergic receptor is a member of the 5-HT_5_ receptor family. This family of receptors is composed of 5-HT_5A_, which is expressed in humans, rats, and mice, and 5-ht_5b_, expressed in rats and mice [143]. In rodents, the 5-HT_5A_ receptor is widely distributed in regions such as the hippocampus, cortex, cerebellum, olfactory bulb, habenula, and spinal cord. Due to its location, this receptor may be involved in the processes of memory consolidation, learning, motor control and in pathophysiology of psychiatric disorders [143]. The 5-HT_5A_ receptor is the least understood receptor in the entire family of serotonergic receptors. The scarcity of studies focusing on its functional characterization is a reason for this. To elucidate the function of this receptor, the effects of the depletion of 5-HT_5A_ receptor and its antagonism on the cortex of mice were observed. The study revealed that 5-HT_5A_ receptors produce internal rectifying potassium current. This current was not observed in mice that received the 5-HT_5A_ antagonist SB-699551 and in 5-HT5_A_ knockout mice; in these animals, an increase in another inhibitory current mediated by 5-HT_1A_ receptors was observed, which suggests an interaction between them. Regarding behavior, 5-HT_5A_ knockout mice did not show anxiety symptoms, unlike the 5-HT_1A_ knockout mice. Activation of the 5-HT_1A_ receptor is a pharmacological strategy for the treatment of mood disorders. Thus, as a possible interaction between receptors has been demonstrated, studies with selective 5-HT_5A_ agonists can better clarify the function of this receptor and the level and mechanism of interaction with the 5-HT_1A_ receptor [144,145].

The 5-HT_5A_ receptor is coupled to the Gα_i/0_ protein through which it promotes the inhibition of AC activity and blocks cAMP production. These receptors have been found in the hippocampus, cerebellum, hypothalamus, thalamus, amygdala, and striatum, but can also be expressed in other regions where they have not yet been identified. LSD and 5-carboxamidotriptamine (5CT) are 5-HT_5A_ receptor agonists; the first is a partial agonist and the second is an agonist with affinity and potency superior to 5-HT itself. The coupling of this receptor to the Gα_i/0_ protein results not only in the blocking of cAMP production and consequent reduction in PKA activity, but also in the inhibition of the adenosine diphosphate enzyme ADP-ribosyl cyclase, which is responsible for the synthesis of the calcium-mobilizing messengers, the cyclase ADP-ribosyl (cADPR) and nicotinic acid adenine dinucleotide phosphate (NAADP). Thus, the activation of 5-HT_5A_ and inhibition of cyclase ADP-ribosyl inhibit the mobilization of intracellular calcium [146,147]. However, this mobilization can still occur due to the activity of IP3, which stimulates the release of calcium reserves in the ER, increasing the intracellular calcium concentration. This increase can stimulate the potassium output currents through a GIRK1 channel that is also coupled to the 5-HT_5A_ receptor. These potassium currents are inhibited by antagonists of that receptor (Figure S5 (Supplementary Material)) [12].

#### 6.1.1. Treatment of Anxiety 

There are few studies indicating that the 5-HT_5A_ receptor plays important roles in the neurobiology of anxiety disorders. Fear memory and fear conditioning are associated with the anxious phenotype. Individuals with social anxiety and specific phobias tend to have memories of fear situations and exposure to constant danger, such as images of personal and social fear and fear of physical dangers, which seem to have great potential to occur. A recent study proposed that blocking 5-HT_5A_, 5-HT_6_, and 5-HT_7_ serotonergic receptors in BLA can facilitate the extinction of fear memories, which would alleviate the anxious condition. This hypothesis was also tested with the same receptors but located in the CA1 region of the hippocampus; however, no benefits were observed. The 5-HT_5A_ antagonist SB699551, the 5-HT_6_ antagonist SB-271046A, and the 5-HT_7_ antagonist SB269970 were used. The combined administration of these substances in BLA favors the extinction of the contextual fear conditioning implicated in anxiety disorders [148,149].

SB699551-A and A-843277 are 5-HT_5A_ antagonists and were administered to rats submitted to tests in anxiety assessment models. SB699551-A showed anxiolytic properties, while A-843277 demonstrate antidepressant-like activity. In the OF test, both compounds produced a sedative effect, with depression of the motor system. Animals that received o-843277 squirmed, which suggests abdominal pain. In the forced swim test, SB699551-A produced no effect and A-843277 despite reducing mobility, caused contortions, which may be responsible for reducing mobility. Although interesting results have been observed for the exploration of the anxiolytic potential of 5-HT_5A_ antagonists, these data are still inconclusive, since the reduction in anxious behavior was not observed for both compounds in all tests [150].

#### 6.1.2. Treatment of Depression 

5-HT_5A_ receptors may be involved in signaling triggered by antidepressants such as SSRIs. In [151], the authors investigated this hypothesis by analyzing the activity of 5-HT_5A_ receptors expressed in the parvalbumin interneurons, GABAergic neurons found in the DG of the hippocampus. Chronic SSRIs administration promotes the translocation of 5-HT_5A_ receptors to the membrane, where they acquire the active form. Stimulation of these receptors reduces cAMP levels and PKA activity, which results in the inhibition of Kv3.1β potassium channels and, consequently, reduction of parvalbumin neuron firing. Through this mechanism, 5-HT_5A_ receptors can delay or reduce behavioral and physiological responses to antidepressants. Thus, 5-HT_5A_ antagonists could induce the opposite action, generating or intensifying antidepressant activity. An example of this is the action of the 5-HT_5A_ antagonist A-843277, which exhibited antidepressant-like activity in rats submitted to the forced swim test [150]. There is lack of studies that investigate the antidepressant potential of compounds that act on the 5-HT_5A_ receptor, even though there are analyses that correlate it to the neurobiology of depression, which makes this receptor a potential target, but recent and little have explored the possibilities of new antidepressant therapies [70,152].

## 7. 5-HT_6_ Receptors

### 7.1. Mechanism of Pharmacological Action

The 5-HT_6_ serotonergic receptor is expressed in greater density in regions of the CNS such as olfactory tubercle, frontal and entorhinal cortex, hippocampus, NAc and striatum, and in lower density in the hypothalamus, amygdala, substantia nigra, and diencephalic nuclei. The 5-HT_6_ receptor is coupled to the Gα_s_ protein, through which it stimulates AC activity, increasing the cAMP production and PKA activity. The carboxylic terminal of the 5-HT_6_ receptor interacts with Fyn-tyrosine kinase, a protein which is part of the Src family of non-receptor kinases. Through the interaction with Fyn-tyrosine kinase, the 5-HT_6_ receptor signals the activation of ERK 1 and 2. Fyn-tyrosine kinase is able to interact with Tau, a protein associated with the microtubule that is involved in the development of neurodegenerative diseases. When ERK1 is active, it participates in the phosphorylation of the Tau protein, enabling an association between 5-HT_6_ receptor modulation and the pathophysiology of neurodegenerative diseases. The 5-HT_6_ receptor also interacts with protein-1 linked to the Jun activation domain (Jab-1). The activation of 5-HT_6_ induces the translocation of Jab-1 to the nucleus, favoring the interaction between Jab-1 and c-Jun. The activation of the 5-HT_6_ receptor also generates inhibition of GIRK channels, reducing potassium currents (Figure S6 (Supplementary Material)) [12,153].

The 5-HT_6_ receptor is capable of interacting with mTOR, intensifying its signaling pathway. The administration of a 5-HT_6_ agonist stimulated the mTOR pathway in PFC neurons, mainly GABAergic neurons. The 5-HT_6_ receptor interacts with proteins in the mTOR pathway, including the mTOR regulatory protein (Raptor), mTOR itself, and GβL, a positive regulator of the mTOR pathway, which together form the rapamycin-sensitive mTOR complex 1 (mTORC1). This interaction stimulates the mTOR pathway, which is involved in the development of psychiatric diseases such as schizophrenia [154]. 5-HT_6_ receptors are found on the post-synaptic membrane of GABAergic neurons. Blocking the 5-HT_6_ receptor in these neurons inhibits the release of GABA, increasing cholinergic, glutamatergic, and monoaminergic neurotransmission [155].

#### 7.1.1. Treatment of Anxiety 

Dorsomedial PFC (dmPFC) is a brain region with a high expression of 5-HT_6_ receptors. This region participates in information processing and may be involved in anxiety modulation mechanisms. In an attempt to investigate the importance of this region for the development and treatment of anxiety disorders, and the 5-HT_6_ receptors expressed in it, the 5-HT_6_ agonist EMD386088 and the SB271046 antagonist were injected into the dmPFC of mice. EMD386088 caused an anxiolytic effect in the OF, EPM, and social interaction tests, with intense reduction in spontaneous excitatory post-synaptic currents (EPSCs) and, to a lesser extent, reduction in spontaneous inhibitory post-synaptic currents (IPSCs). This imbalance results in increased levels of GABAergic transmission. The regulation of anxiety through dmPFC may occur due to its glutamatergic projections for BNST and CeA, cerebral structures implicated in the neurobiology of anxiety. Thus, the increase in GABAergic transmission in dmPFC inhibits the excitatory transmission that starts from dmPFC towards BNST and CeA, reducing the activity of the amygdala. In contrast, SB271046 exhibited the opposite effect, with anxiogenic symptoms in the OF, EPM, and social interaction tests [156].

Similarly, the intra-hippocampal administration of the 5-HT_6_ agonist EMD386088 promoted anxiolytic and antidepressant effects in rats in forced swim, EPM, and Vogel conflict tests. The 5-HT_6_ antagonist SB399885 was also injected into the hippocampus and exhibited an anxiogenic effect in these tests [157,158]. Conversely, DNA1184 is an antagonist of 5-HT_6_ and 5-HT_7_ receptors and has been tested in mice submitted to four-plates, EPM, marble burying, and Vogel conflict tests. The compound exhibited anxiolytic activity in all animal models evaluated; however, a more pronounced activity was observed in the Vogel conflict test [159].

The 5-HT_6_ antagonist SB399885 has been tested to treat symptoms of PTSD, an anxiety disorder. Intraperitoneal administration of SB399885 reduced 5-HT levels in the amygdala, inhibiting local neuronal overactivity, without affecting motor activity. This anxiolytic effect was observed in the EPM test [160]. 5-HT_6_ agonists WAY-208466 and WAY-181187 were subcutaneously administered to rats, which were subsequently submitted to evaluation in the forced swimming, marble burying, and in the novelty-induced hypophagy tests. Both compounds showed anxiolytic and antidepressant activity in all tests, with advantages over drugs available so far, with rapid onset of action [161]. Intra-hippocampal administration of the selective 5-HT_6_ receptor antagonist SB-258585 induced an effect of the antidepressant and anxiolytic types in rats. These properties were observed in the Vogel conflict and forced swim tests. The mechanism of anxiolytic action of 5-HT_6_ receptor antagonists and agonists has yet to be elucidated. It is known that these receptors are involved in the modulation of GABAergic, glutamatergic, cholinergic and monoaminergic neurotransmission; however, it is not yet possible to attribute to any of these pathways, in particular, the observed effects, which may vary according to the injection site, compounds, location, and density of receptors [162].

#### 7.1.2. Treatment of Depression 

To learn about the role of 5-HT_6_ agonists and antagonists on depression, more specifically, on depression associated with Parkinson’s disease, [163] tested the 5-HT_6_ receptor agonist WAY208466 and SB258585 antagonist. The activation of the 5-HT_6_ receptor in the pre-limbic cortex through WAY208466 in rats with depressive phenotype resulted in an antidepressant effect. However, the administration of the same compound in rats that did not have a depressive phenotype had the opposite effect. In turn, the receptor blockade induced by SB258585 showed antidepressant activity in rats without a depressive phenotype and increased depressive behavior in rats with this phenotype. This study showed an increase in glutamate production induced by 5-HT_6_ agonist WAY208466, and reduction in that production after treatment with the SB258585 antagonist.

EMD386088 is a partial 5-HT_6_ receptor agonist. The acute and chronic intraperitoneal administration of this substance in rats exhibited antidepressant activity. This effect was noticed in the forced swimming and OF tests, performed 30 min and 24 h after EMD386088 administration. The administration of the selective 5-HT_6_ receptor antagonist SB271046 blocked the antidepressant activity of the compound [164]. Another study analyzed neurochemical data collected after analyses in animals that received EMD386088 and exhibited antidepressant activity. The activation of the 5-HT_6_ receptor promotes increased activation of the dopaminergic system, but not of serotonergic and adrenergic systems. In addition, EMD386088 has considerable activity on the DA transporter, which is inhibited by the compound. These data indicate an important involvement of dopaminergic signaling in the antidepressant mechanism of EMD386088, which is confirmed by the abolition of the antidepressant activity after the administration of the dopaminergic receptor antagonist D_1_ SCH23390 and D_2_ sulpiride antagonist [165].

Subchronic ketamine administration to mice induces depression-like behavior. This behavior was reversed by acute treatment with E-6837, 5-HT_6_ agonist, and SB-271046, 5-HT_6_ antagonist. Both substances significantly reduced immobility in tail suspension and forced swimming tests, indicating an antidepressant effect. The authors of this study suggest that 5-HT_6_ receptor agonists may enhance the release of GABA in the limbic system. The increased activity of GABAergic interneurons can restore the activity of the mesocortical pathway, compromised by the depressive condition. This restoration involves the connection between GABAergic and dopaminergic neurons of the VTA. The antidepressant activity of both compounds is contradictory to the theoretically paradoxical action between 5-HT_6_ agonists and antagonists. This similarity of results may occur due to the varied location of 5-HT_6_ receptors in different neurons, including cholinergic, GABAergic, and glutamatergic. New studies that investigate the signaling pathways involved in the activation and blockade of the 5-HT_6_ receptor in each type of neuron could clarify the occurrence of the same effect for agonists and antagonists of that receptor [166].

## 8. 5-HT_7_ Receptors

### 8.1. Mechanism of Pharmacological Action

The 5-HT_7_ serotonergic receptor is coupled to the Gα_s_ protein, through which it stimulates AC activity and promotes an increase of cAMP levels and PKA activation. This signaling induces AKT and ERK activation through the Ras protein. AKT and ERK participate in several intracellular processes, among them, gene transcription, cytoskeleton formation, and neuroprotection. Although AKT activation is generally mediated by an increase in cAMP and calcium levels, while an increase in calcium inhibits ERK activation, in the case of 5-HT_7_ receptor activation, no changes in calcium concentration were observed, but AKT and ERK were activated. The 5-HT_7_ receptor also interacts with the Gα_12_ protein, which stimulates the signaling pathway of Rho GTPases, important for stabilizing microtubules and actin reorganization. The stimulation of proteins in Rho pathway occurs through the activation of the guanine nucleotide exchange factor (GEF), which stimulates cell division cycle 42 (Cdc42) proteins, which activates the SRF, whose role involves the stimulation of the serum response element (SRE). In addition to these proteins, Gα_12_ also interacts with heat shock protein 90 (HSP90), A-kinase anchoring proteins (AKAPs), proteins from the ezrin–radixin–moesin family (ERM), non-receptor tyrosine kinases (nRTKs), cadherins, phosphatases, and proteins in the occlusive zone. Thus, activation of the 5-HT_7_ receptor may be necessary for neurogenesis and reorganization of the dendritic morphology (Figure S7 (Supplementary Material)) [167,168].

The 5-HT_7_ receptor is widely expressed in the nervous system in regions such as spinal cord, thalamus, hypothalamus, hippocampus, PFC, *striatum*, and amygdala. Due to its location, the 5-HT_7_ receptor is associated with the regulation of the circadian rhythm, thermoregulation, nociception, and memory processing. Its role in CNS morphogenesis was assessed after stimulation with the selective 5-HT_7_ agonist LP-211. The activation of this receptor caused a decrease in the number of dendritic spines in striatal and cortical neurons, confirming its role on the neurogenesis and density of dendritic spines. These activities were mediated by the Cdc42 protein, whose activation was increased by stimulation of the 5-HT_7_ receptor [169].

#### 8.1.1. Treatment of Anxiety 

There are few studies that have investigated the role of 5-HT_7_ receptors on anxiety. To assess the performance of these receptors in anxiety associated with Parkinson’s disease, 5-HT_7_ agonist AS19 and 5-HT_7_ antagonist SB269970, administered via pre-limbic intra-cortex, were tested. AS19 agonist showed anxiolytic activity, observed in OF and EPM tests. AS19 increased the DA, 5-HT, and NE levels in the mPFC, ventral hippocampus, and amygdala, while SB269970 decreased monoamine levels in these regions and triggered the anxiogenic effect [170]. Compounds PZ-1417 and PZ-1150 are 5-HT_7_ receptor antagonists and were intraperitoneally administered in male albino and Swiss mice and in male Wistar rats. Antidepressant and anxiolytic properties of both compounds were observed in the forced swim, tail suspension, and four-plates tests. This activity was compared to the activity of SB269970, a 5-HT_7_ receptor antagonist, used as reference. However, the effect observed for PZ-1417 and PZ-1150 was greater than that observed for SB269970 [171].

The antidepressant and anxiolytic activities of SB269970 were also compared to the effect of diazepam and imipramine antidepressants. SB269970 showed anxiolytic and antidepressant effect in the four-plates, EPM, and Vogel conflict tests, and antidepressant activity in the forced swim test. However, the effect observed was less intense than that promoted by the drugs used as reference [172]. The antagonism of the 5-HT_7_ receptor by SB269970 was also able to reduce prenatal stress, which is related to behavior like anxiety and depression. Stress induces an increase in EPSCs and decrease in IPSCs, stimulating neuronal excitability in DRN. Blocking the 5-HT_7_ receptor by SB269970 decreases cortical glutamatergic transmission and contributes to restoring balance between EPSCs and IPSCs, reducing neuronal overactivation induced by prenatal stress [173]. Directly or indirectly, 5-HT_7_ receptor antagonists appear to trigger anxiolytic effects. Although the mechanism responsible for this effect is still unknown, the influence of this receptor on monoamine levels and on the excitatory/inhibitory balance, which modulate neuronal excitability, which is critical for the development of anxious symptoms, is known.

#### 8.1.2. Treatment of Depression 

5-HT_7_ knockout mice exhibited antidepressant behavior in the forced swim test. This observation supported the hypothesis that blocking this receptor may attenuate depression-like behavior. The selective 5-HT_7_ receptor antagonist SB269970 showed antidepressant activity in the tail suspension test and the forced swim test. 5-HT_7_ knockout mice were submitted to the same tests and the results were similar, indicating that inhibition of the 5-HT_7_ receptor is a potential pharmacological strategy for the treatment of depressive disorders [174].

This antidepressant action that results from blocking the 5-HT_7_ receptor may occur due to the capacity of this receptor to modulate monoamine levels. An increase in the release of 5-HT was observed after SB269970 administration. This compound can exert antidepressant activity administered as monotherapy, but its association with other antidepressants has been investigated and shown to be even more advantageous. Combined therapy using SB269970 and citalopram proved to be effective for the treatment of depression, since the SSRI action was enhanced by the administration of the 5-HT_7_ antagonist, promoting a marked increase in the available 5-HT concentration. This effect was quantified by the decrease of the immobility duration [175].

Another association with SB269970, which exhibited intense antidepressant activity, was the combined therapy with imipramine, which had its effects enhanced by SB269970 [176]. The calcium-binding protein S100B reduces cAMP production in astrocytes. Overexpression of this protein disrupts the cAMP pathway and induces behaviors similar to depression, observed in the forced swim test. SB269970 administration and the consequent blocking of the 5-HT_7_ receptor neutralizes the effects of overexpression of this protein, leading to an antidepressant effect, which suggests that the action of S100B on cAMP levels may occur through an interaction with the receptor 5-HT_7_ [177].

Another 5-HT_7_ receptor antagonist, JNJ-18038683, exhibited antidepressant activity in the tail suspension test, with increased serotonergic transmission observed after administration of the compound. JNJ-18038683 was also able to intensify the action of citalopram, both in rats and in humans [178]. The ability of 5-HT_7_ receptor antagonists to increase the release of 5-HT is effective in reducing depressive symptoms and is even more useful when this activity is associated with the effect of SSRIs, whose antidepressant action is enhanced.

## 9. Phytochemical Compounds of Natural Origin Acting via 5-HT Receptors: Preclinical and Clinical Research and Future Perspectives in the Treatment of Anxiety and Depression

The role of herbal medicine in the treatment of anxiety and depressive disorders has been established over the years, especially the use of *Hypericum perforatum* (St. John’s wort) [179] and *Piper methysticum* (Kava) [180], among others, as phytotherapeutic preparations that present respectable clinical evidence. The aim of this section is to summarize evidence from the last 5 years of research in preclinical studies and clinical trials involving medicinal plants with potentially positive effects on anxiety and depressive disorders. Studies involving up to three herbal medicines were included and studies detailing secondary analyses of primary data were not included.

**Figure 8:**
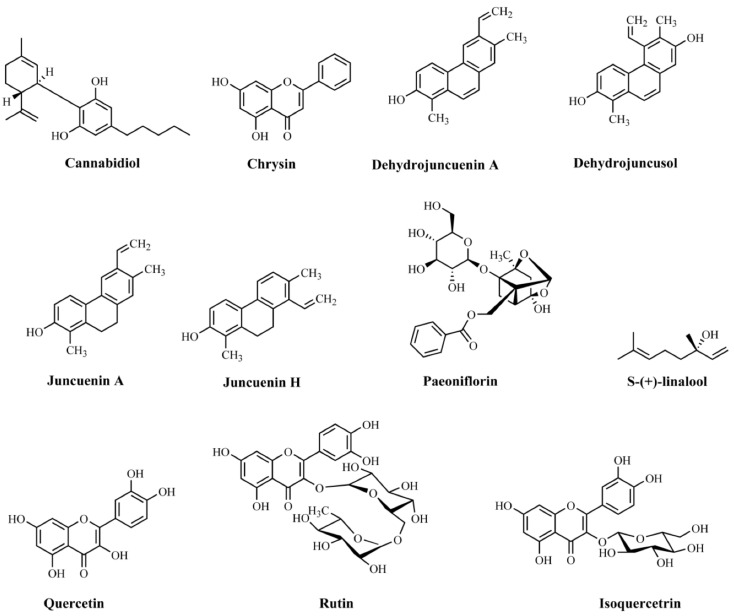
Structural formulas of phytochemicals cited in Table 1 and Table 2.

Table 1 and Table 2 present some isolated phytochemicals and plant extracts, respectively, that produce antidepressant-like or anxiolytic-like effects on pre-clinical animal models. The first table (Table 1) shows the names of phytochemicals and their plant source, animal models and specimen assayed, administration scheme, major findings, and, if possible, the mechanism of action evaluated in studies. The second table (Table 2) presents plant extracts, compounds detected, animal models and specimen assayed, administration scheme, major findings, and, if possible, the mechanism of action evaluated in studies. The structural formulas of these phytochemicals cited in Table 1 and Table 2 are presented in Figure 8.

Tables S2 and S3 (Supplementary Material) present isolated phytochemicals and herbal medicines, respectively, submitted to clinical trials to verify their antidepressant and/or anxiolytic effectiveness. The first table (Table S2) described the names of phytochemicals and their plant source, disorder or symptoms, diagnostic instruments, posology, and major findings. The second table (Table S3) presents herbal medicines and the marker compound, disorders or symptoms, diagnostic instruments, posology, and major findings. The structural formulas of phytochemicals cited in Tables S1 and S2 are presented in Figure S8 (Supplementary Material).

As can be observed, pre-clinical and clinical studies using plant extracts or herbal medicines are more frequent, exploring a variety of different specimens. Concerning clinical trials, only four phytochemicals (crocin, curcumin, l-theanine, and valeric acid) have been recently investigated. About herbal medicines, an emerging interest regarding two specimens has been noted: *Crocus sativus* in depressive disorders and *Lavandula angustifolia* in anxiety disorders.

Evidence that supports the effect of herbal medicines on anxiety and depressive disorders has grown in the past years, and many of these featured only in isolated, short-term, and small sample studies. Therefore, it is still required to conduct further robust and larger studies [179,180,210]. In addition, the co-prescription of certain herbal medicines with pharmaceuticals should provide beneficial and additional effectiveness, as seen in Table 2 and Table S2, and this approach remains an area of potential future research.

## 10. Conclusions

Over the past twenty years, there has been an important advancement regarding the understanding of the “*serotonergic receptosome*” signaling mechanisms, both receptors coupled to the G protein and receptors coupled to ion channels, and their correlations with the treatments of anxiety and depression. As for GPCRs, an advancement that is still crucial in the field of pharmacology and neurochemistry is the precise determination of where 5-HT receptors activate G proteins in cells and the activation dynamics of these proteins. In addition to G protein signaling, the identification of regions of interaction of serotonergic receptors using genetic or proteomic strategies is effective for the understanding of new signaling pathways related to these receptors. As an example, the new signaling pathways related to 5-HT_6_ and 5-HT_7_ receptors that play a crucial role in neurodevelopment can be highlighted.

New neuropharmacological studies should be carried out to improve the understanding of the function of the 5-HT system in the CNS and, mainly, in the periphery of the human body, as well as to elucidate the biological responses associated with total, partial, and inverse agonists and 5-HT receptor antagonists and their functional selectivity. Additional data are needed to determine the interest in specific diseases of the CNS and other organ systems. In the present study, we encourage researchers in the field to investigate new anxiolytic and antidepressant therapies based on the modulation of the “*Serotonergic Receptosome*”, once many 5-HT receptors are potential and little explored targets for the possibilities of new therapies for mood disorders.

## Figures and Tables

**Table 1:** Phytochemicals that produce antidepressant-like or anxiolytic-like effects on animal models.

Compound	Plant/Extract	Model/Test Used	Animal Species	Administration	Major Findings	Mechanisms of Action	Reference
Cannabidiol	*Cannabis sativa*/not informed	Elevated plus-maze and novelty suppressed feeding tests	Male C57BL/6 mice	Intraperitoneal administration (30 mg/kg) 2 h after daily stressor for 14 days	Anxiolytic effectiveness	Not assayed	[181]
Chrysin	Not informed	Splash, rota-rod, and tail suspension tests	C57B/6J mice	Oral administration (10 ml/kg) 30 min before tests	Antidepressant effectiveness	Decrease in the levels of 5-HT in the hippocampus	[182]
Dehydrojuncuenin A (4)	*Juncus setchuenensis*/ethanolic extract	Elevated plus maze	Male CD-1 mice	Oral administration (5, 10, or 20 mg/kg) 30 min before test	Anxiolytic effectiveness	Not assayed	[183]
Dehydrojuncusol (2)	*Juncus setchuenensis*/ethanolic extract	Elevated plus maze	Male CD-1 mice	Oral administration (2.5, 5, or 10 mg/kg) 30 min before the test	Anxiolytic effectiveness	Not assayed	[183]
Juncuenin A (3)	*Juncus setchuenensis*/ethanolic extract	Elevated plus maze	Male CD-1 mice	Oral administration (2.5, 5, 10, or 20 mg/kg) 30 min before test	Anxiolytic effectiveness	Not assayed	[183]
Juncuenin H (1)	*Juncus setchuenensis*/ethanolic extract	Elevated plus maze and locomotor test	Male CD-1 mice	Oral administration (5, 10, or 20 mg/kg) 30 min before tests	Anxiolytic effectiveness	Reductions in the levels of 5-HT and 5-HT/DA metabolites in the cerebral cortex and hippocampus.	[183]
Paeoniflorin	Not informed	Elevated plus maze test	Male Sprague–Dawley rats	Intraperitoneal administration (5, 10, and 20 mg/kg) 1 h before test	Anxiolytic effectiveness	Increased levels of 5-HT and 5-HIAA in the hippocampus	[184]
S-(+)-linalool	*Cinnamomum osmophloeum*/essential oil	Elevated plus maze test	Male ICR mice	Oral administration (250 and 500 mg/kg) 1 h before tests for 14 days	Anxiolytic effectiveness	Anxiolytic effect via modulation of 5-HT	[185]

Abbreviations: 5-HT = 5-hydroxytryptamine, 5-HIAA = 5-hydroxyindoleacetic acid, DA = dopamine.

**Table 2:** Plant extracts that produce antidepressant-like and/or anxiolytic-like effects in animal models.

Plant/Extract	Model/Test Used	Animal Species	Administration	Major Findings	Mechanisms of Action	Reference
*Achyranthes aspera*/methanolic extract	Hole cross, OF, forced swimming, tail suspension, elevated plus maze, and light/dark	Mice	Oral administration (50, 100, and 200 mg/kg) 30 min before tests	Anxiolytic and antidepressant effectiveness	Not assayed	[186]
*Aloysia triphylla*/methanolic, dicloromethane and hexanic extracts	Elevated plus maze test	Male ICR mice	Oral administration (125, 250, 500, and 750 mg/kg) 30 min before test	Anxiolytic effectiveness	Interaction with serotonergic transmission	[187]
*Annona vepretorum*/essential oil	Elevated plus-maze, hole-board, open-field, rota-rod, and tail suspension tests	Male albino Swiss mice	Intraperitoneal administration (25, 50, and 100 mg/kg)	Anxiolytic and antidepressant effectiveness	Not assayed	[188]
*Camellia euphlebia*/aqueous extract	Light/dark box, elevated plus maze, forced swimming, tail suspension, and open-field tests	Male Kunming mice	Intragrastrical administration (100, 200, or 400 mg/kg) 1 h before tests for 7 days	Anxiolytic and antidepressant effectiveness	Not assayed	[189]
*Camellia sinensis*/aqueous and ethanolic extracts	Elevated plus maze and OF tests	Male C57BL/6J mice	Oral administration (50 and 100 mg/kg) 1 hour before test	Anxiolytic effectiveness	Activation of serotonin 5-HT_1A_ receptors	[190]
*Cananga odorata*/essential oil	Open field, elevated plus maze, and light/dark box tests	ICR mice	10 mL inhalation 10 min before tests	Anxiolytic effectiveness	Increased 5-HT concentration in the hippocampus of male mice	[191]
*Capparis thonningii*/methanolic extract	Forced swimming, tail suspension, hole-board, light/dark, and elevated plus maze tests	Swiss albino mice	Oral administration (500–4000 mg/kg) 1 h before tests	Anxiolytic and antidepressant effectiveness	5-HT_2_ receptor inhibition	[192]
*Carthamus tinctorius*/ethanolic extract	Elevated plus maze and forced swim tests	White albino rats	Oral administration (100 and 200 mg/kg) 1 h before tests	Anxiolytic and antidepressant effectiveness	Not assayed	[193]
*Cocos nucifera*/hydroalcoholic extract	Elevated plus maze, hole-board, forced swimming, tail suspension, and open-field tests	Swiss mice	Oral administration (50, 100, or 200 mg/kg) 1 h before tests	Anxiolytic and antidepressant effectiveness	Inhibition of the 5-HT system	[194]
*Coriandrum sativum*/aqueous extract	Elevated plus-maze test and light/dark transition	Male Swiss albino mice	Oral administration (100, 200, or 400 mg/kg) 2 hours/day for 14 days	Anxiolytic effectiveness	Decrease in levels of NE, DA, and 5-HT in cortex, hippocampus, cerebellum, and brain stem	[195]
*Cuscuta reflexa*/methanolic extract	Elevated plus maze and light/dark box tests	Swiss albino mice	Oral administration (200 and 400 mg/kg) 30 min before tests for 14 days	Anxiolytic effectiveness	Not assayed	[196]
*Hoodia gordonii*/aqueous extract	Forced swim and OF tests	Male Swiss mice	Oral administration (25 and 50 mg/kg) 1 h before tests	Antidepressant effectiveness	Results showed that only 5-HT monoamine was significantly increased after acute *H. gordonii* administration	[197]
*Maerua angolensis*/crude extract	Novel tank and light/dark box tests	Zebrafish	1.0, 0.3, 0.1 mg/ml diluted in water 20 min before tests	Anxiolytic effectiveness	Direct or indirect effect on the activation of GABA_A_ and 5-HT_1–3_ receptor	[198]
*Morinda citrifolia*/methanolic extract	Elevated plus maze, light/dark transition, and tail suspension tests	Male Swiss albino mice	Oral administration (0.5, 1, 3 g/kg) 1 h before tests	Anxiolytic and antidepressant effectiveness	Not assayed	[199]
*Newbouldia laevis*/hydroethanolic extract	Hole-board, open-field, elevated plus maze, light/dark box exploration, social interaction, forced swim, and tail suspension tests	Mice	Intraperitoneal administration (50, 100, 200, 400, and 800 mg/kg)	Anxiolytic and antidepressant effectiveness	Not assayed	[200]
*Paederia foetida*/aqueous, ethanolic, and ethyl acetate extracts	Hole cross, OF, and elevated plus maze tests	Albino mice	Oral administration (400 mg/kg)	Anxiolytic effectiveness	Not assayed	[201]
*Pimpinella anisum*/aqueous and ethanolic extracts	Forced swimming, tail suspension tests	Mice	Intraperitoneal administration (50, 100, and 200 mg/kg)	Antidepressant effectiveness	Not assayed	[202]
*Salvia miltiorrhiza*/essential oil	Elevated plus maze, social interaction, and rota-rod tests	Male Sprague–Dawley rats	Oral administration (50, 100, and 200 mg/kg) 1 h before tests	Anxiolytic effectiveness	Reduction of monoamine and 5-HT system levels in the cerebral cortex	[203]
*Solanum melongena*/aqueous extract	Elevated plus maze, forced swimming, and tail suspension tests	Male albino mice	Oral administration (100 and 200 mg/kg)	Anxiolytic and antidepressant effectiveness	Increase of 5-HT levels	[204]
*Tagetes erecta*/aqueous extract	Hole-board, open-field, and exploration cylinder tests	Male Swiss Webster mice	Intraperitoneal administration (10, 30, 100 mg/kg) 1 h before tests	Anxiolytic and sedative effectiveness	Not assayed	[205]
*Tagetes lucida*/aqueous extract	Forced swimming	Male Wistar rats	Intragastric administration (50, 100, and 200 mg/kg) 72, 48, 24, 18, and 1 h before test	Antidepressant effectiveness	Modulating the release/reuptake of serotoninby interaction with 5-HT_1A_ and 5-HT_2A_ receptors	[206]
*Tanacetum parthenium*/aqueous extract	Burying behavior, elevated plus maze, forced swimming, and OF tests	Male Swiss Webster mice	Oral administration (0.5, 1.0, 5, 10, 20, and 40 mg/kg) 30 min before tests	Anxiolytic and antidepressant effectiveness	Not assayed	[207]
*Tilia americana*/hexanic, ethyl acetate, and methanolic extracts	Elevated plus maze and hole-board tests	Male CD-1 mice	Intraperitoneal administration (100 mg/kg) 50 min before test	Anxiolytic and sedative effectiveness	Production of the anxiolytic effect reversed in the presence of 5-HT_1A_ receptor antagonists	[208]
*Ziziphus mucronata*/hydromethanolic extract	Elevated plus maze, light/dark, and forced swim tests	Adult male Wistar rats	Oral administration (300 mg/kg) 30 min before tests	Anxiolytic and antidepressant effectiveness	Modulation of serotonergic and noradrenergic systems	[209]

Abbreviations: 5-HT = 5-hydroxytryptamine, DA = dopamine, GABA = gamma-aminobutyricacid, GABA_A_ = GABA type A receptor, NE = noradrenaline, OF = open-field.

## Data Availability

No new data were created or analyzed in this study. Data sharing is not applicable to this article.

## Supplementary Materials

The following are available online at https://www.mdpi.com/1424-8247/14/2/148/s1, Figure S1: Mechanism of action of mirtazapine, Figure S2: The pentameric structure of the 5-HT_3_ receptor, Figure S3: Mechanism of anti-inflammatory action of tropisetron, Figure S4: 5-HT_4_ receptor signaling pathways, Figure S5: 5-HT_5A_ receptor signaling pathways, Figure S6: 5-HT_6_ receptor signaling pathways, Figure S7: 5-HT_7_ receptor signaling pathways, Figure S8: Structural formulas of phytochemicals cited in tables 4 and 5, Table S1: 5-HT receptors and their subtypes: central signaling and signal transduction systems, Table S2: Phytochemicals submitted to clinical trials to verify their antidepressant and/or anxiolytic effectiveness, Table S3: Herbal medicines submitted to clinical trials to verify their antidepressant and/or anxiolytic effectiveness.
