# Supplementary material for: Modulation of the Serotonergic Receptosome in the Treatment of Anxiety and Depression: A Narrative Review of the Experimental Evidence
Source: Pharmaceuticals (Basel). 2021 Feb 12;14(2):148. doi: 10.3390/ph14020148 (PMC7918669; doi:10.3390/ph14020148)
Supplement: Supplementary file 1 [file pharmaceuticals-14-00148-s001.pdf]

pharmaceuticals

MDPI

Supplementary Files

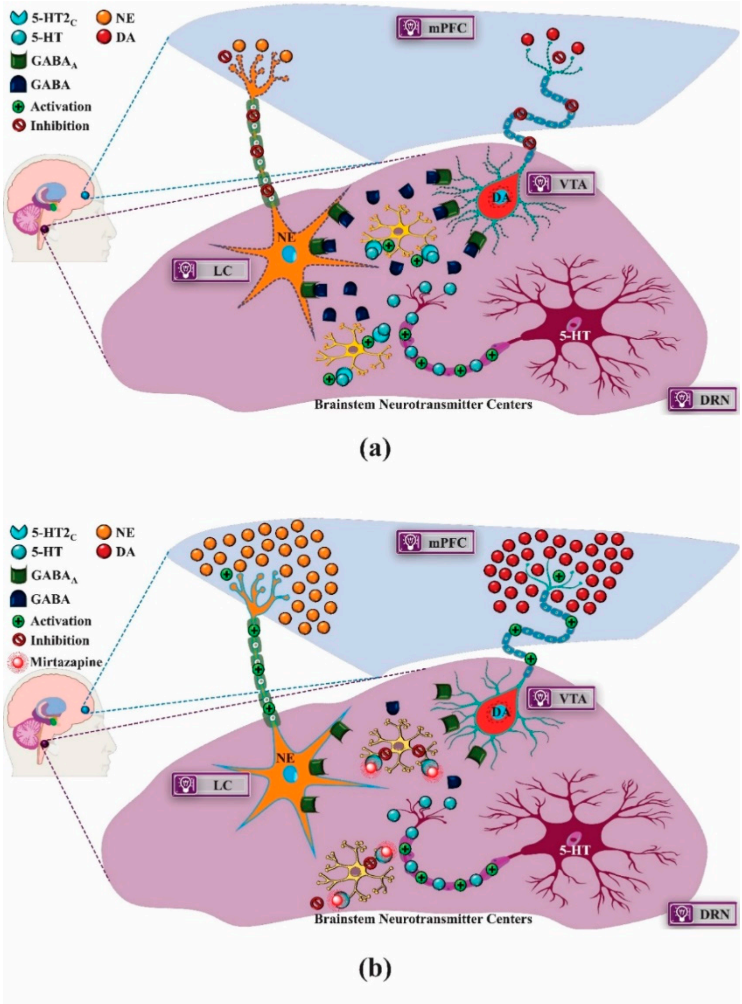

Figure S1. Mechanism of action of mirtazapine: (**a**) Noradrenergic and dopaminergic transmission are inhibited in the medial prefrontal cortex (mPFC) due to the activation of 5-HT_{2C} receptors in gabaergic interneurons. After activation, these interneurons release gamma-aminobutyric acid (GABA), which act on $GABA_A$ receptors present in noradrenergic and dopaminergic neurons, inhibiting them; (**b**) With mirtazapine administration, 5-HT_{2C} receptors located in the gabaergic interneurons are blocked, inhibiting GABA release and disinhibiting underlying noradrenergic and dopaminergic neurons [1], promoting a pronounced antidepressant effect.

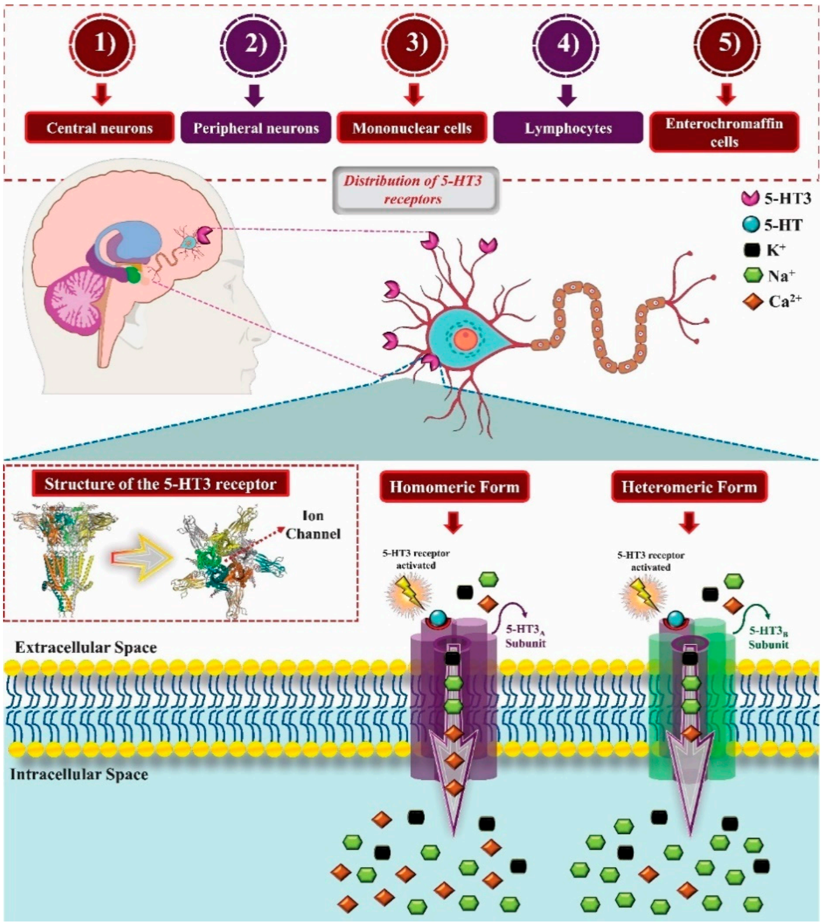

Figure S2. The pentameric structure of the 5-HT3 receptor. The 5-HT3 receptor, as a member of the ligand-gated cation channel family, mainly controls sodium (Na^+), potassium (K^+), and calcium (Ca^{2+}) movements through its channel. These receptors are located both pre-synaptically in nerve terminals, modulating the release of neurotransmitters, and post-synaptically (as shown in the figure) in neuronal and dendrite cell bodies, where they trigger rapid excitatory responses. After stimulation of the pre-synaptic 5-HT3 receptor, neuronal depolarization occurs, promoting calcium influx and the mobilization of intracellular calcium reserves, causing the neurotransmitter's exocytosis. The activation of the post-synaptic receptor (shown in the figure) promotes the influx of sodium, inducing depolarization. Activation of the post-synaptic receptor promotes sodium influx, inducing neuron depolarization.

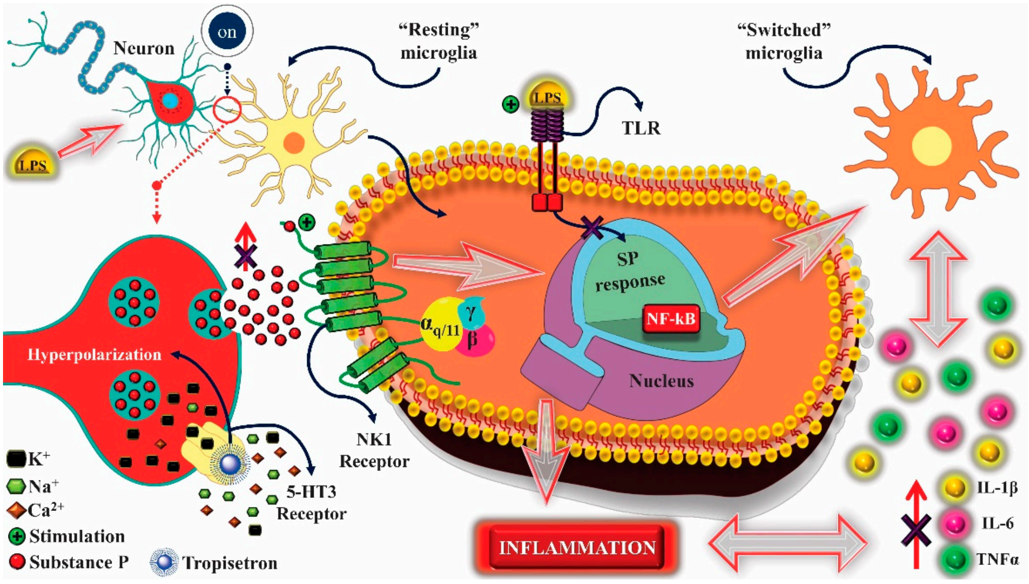

Figure S3. Mechanism of anti-inflammatory action of tropisetron: a mechanistic proposal for the reduction of neuroinflammation via 5-HT3 antagonism. Lipopolysaccharide (LPS) is believed to induce neuroinflammation and microglia activation, causing increased proliferation and morphological changes. The interaction of substance P (SP) with the neurokinin 1 receptor (NK1R) promotes inflammatory immune responses through the glia in a manner similar to its effects on peripheral leukocytes in inflammatory bacterial infections of the CNS. This neuropeptide induces the synthesis of the RelA subunit of the NF-κB in the primary microgliamurine, while LPS stimulates Toll-like receptors (TLR) inducing the synthesis of NF-κB. Subsequently, the activated microglia changes to a microbicidal state, synthesizing important pro-inflammatory cytokines, such as interleukin 1-beta (IL-1β), interleukin 6 (IL-6) and Tumor Necrosis Factor alpha (TNF-α). 5-HT3 receptor antagonists decrease serotonin-induced SP release. Thus, when tropisetrone blocks the 5-HT3 receptor, there is a reduction in the sodium and calcium influx into the neuron and, consequently, a decrease in the release of SP (shown by the arrow with an "X" to the left of the figure). Finally, tropisetron promotes anti-inflammatory response by blocking SP-mediated responses and suppressing LPS-induced neuroinflammation. This anti-inflammatory response occurs indirectly from the inhibition of the SP, NK1R and NF-κB signaling pathway. It is well established in literature that NF-κB is a crucial transcription factor in the synthesis of inflammatory mediators such as IL-1β, IL-6 and TNF-α. As a result of the reduction in their expression, there is a subsequent decrease in the synthesis of these pro-inflammatory mediators (demonstrated by the arrow with an "X" in the lower right part of the figure) [2].

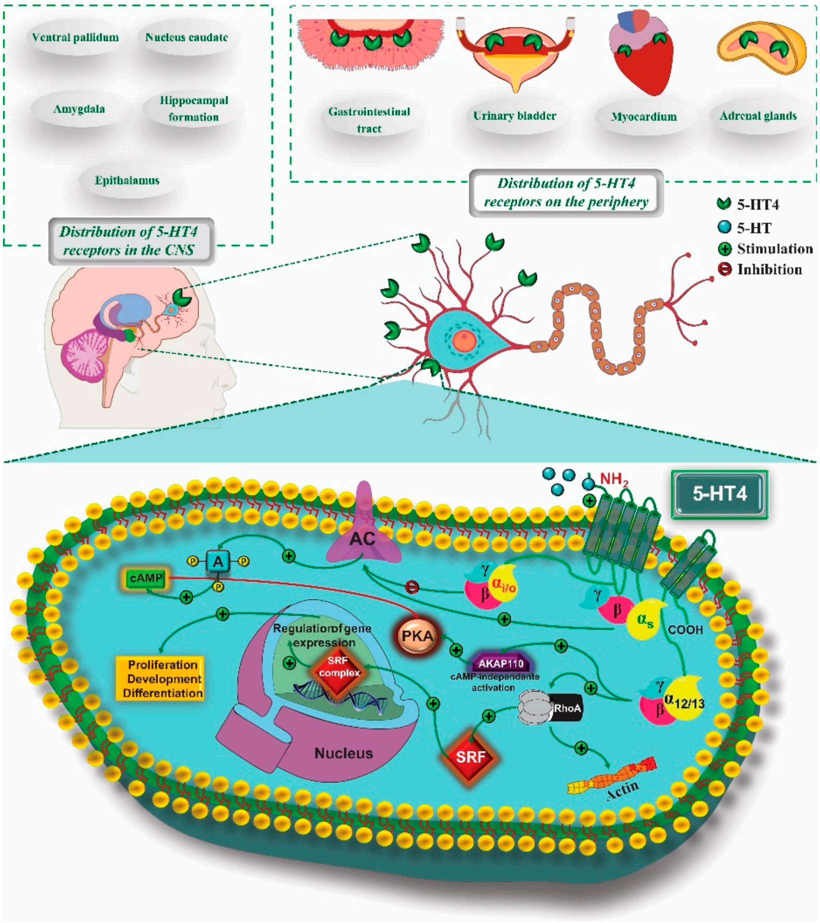

Figure S4. 5-HT4 receptor signaling pathways. The 5-HT4 receptor isoforms activate adenylate cyclase (AC) through coupling with the Gα_s protein. Consequently, there is a conversion of adenosine triphosphate (ATP) into cyclic adenosine monophosphate (cAMP), which is responsible for the activation of protein kinase A (PKA). The 5-HT$_{4B}$ receptor isoform can inhibit AC through the G$\alpha_{i/o}$ protein. The 5-HT$_{4A}$ isoform interacts with the G$\alpha_{12/13}$ protein. The activation of the 5 HT$_4$/G$\alpha_{12/13}$ receptor pathway results in the activation of the gene transcription and actin rearrangement through the GTPaseRasHomolog Family Member A (RhoA) and the serum response factor (SRF). In addition, the activation of the 5 HT$_4$/G$\alpha_{12/13}$ pathway can result in the activation of PKA independent of cAMP by means of the A-kinase anchoring protein (AKAP110) [3,4].

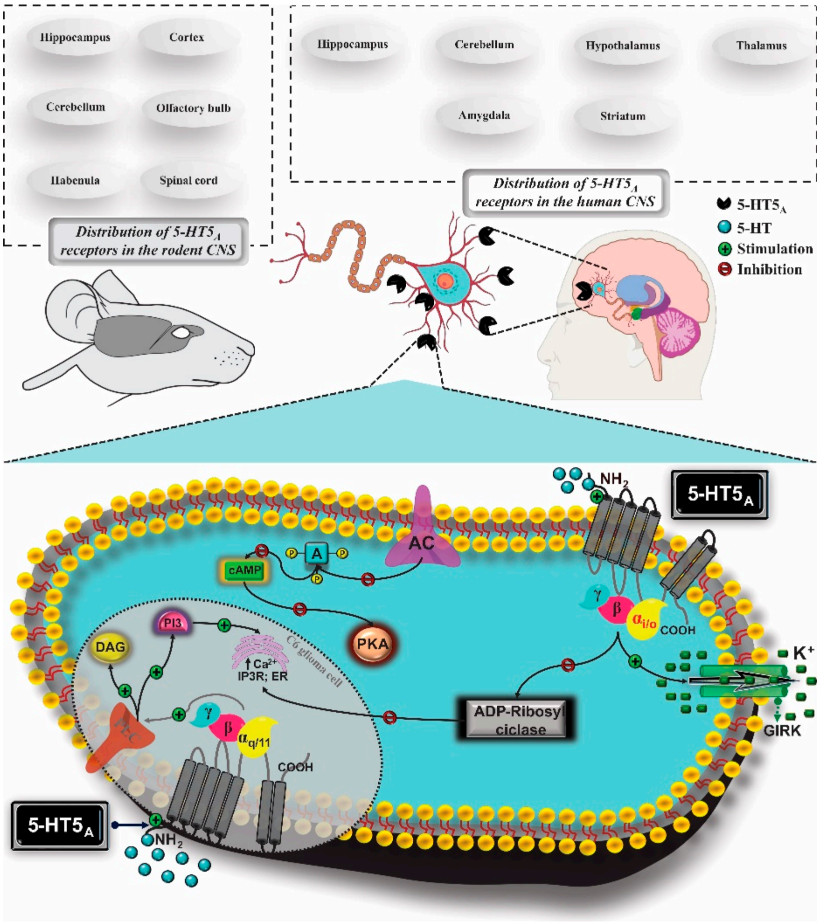

Figure S5. 5-HT_{5A} receptor signaling pathways. The 5-HT_{5A} receptor is coupled to the $G\alpha_{i/0}$ protein. Its activation inhibits the activity of adenylate cyclase (AC) reducing the conversion of adenosine triphosphate (ATP) into cyclic adenosine monophosphate (cAMP), which is responsible for the activation of protein kinase (PKA). The 5-HT_5 receptor also inhibits the activity of ADP-ribosyl cyclase in C6 glioma cells and subsequently down-regulates the calcium levels (Ca^{2+}) inside the cell [5].

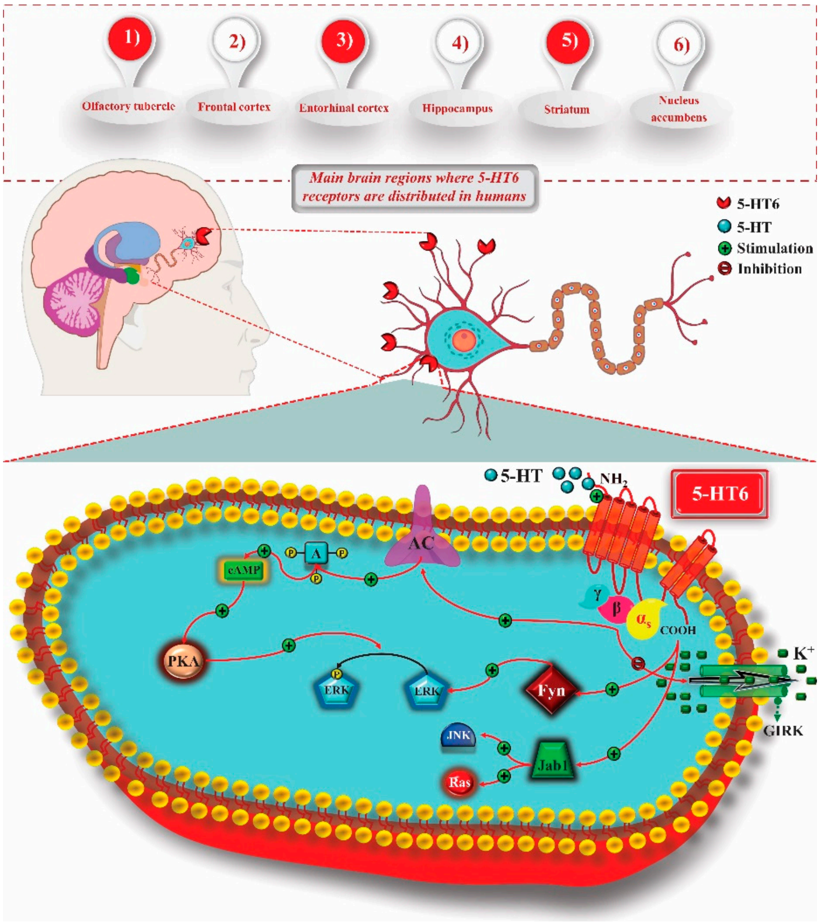

Figure S6. 5-HT$_6$ receptor signaling pathways. The 5-HT$_6$ receptor is coupled to the Gα_s protein. Its activation stimulates the activity of adenylyl cyclase (AC), which converts adenosine triphosphate (ATP) into cyclic adenosine monophosphate (cAMP), which is responsible for the activation of protein kinase A (PKA). In addition, activation of the 5-HT6 receptor inhibits the conductance of the internal rectifying potassium channels (GIRK) in neurons and activates kinases through direct interaction of the receptor C-terminal with interacting proteins, which produces kinase phosphorylation of extracellular signal-regulated kinase (ERK) or translocation of the c-Jun N-terminal kinase protein (JNK) to the nucleus [5].

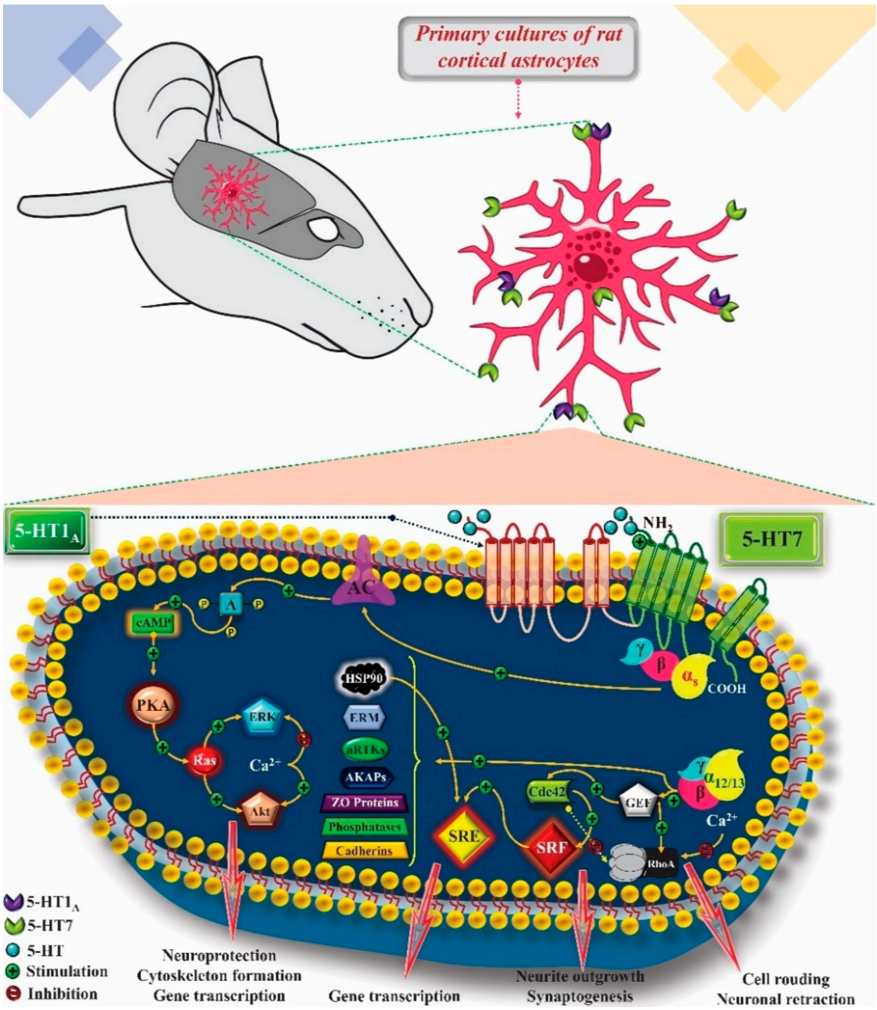

Figure S7. 5-HT_7 receptor signaling pathways. G protein-coupled receptors (GPCRs) were initially assumed to exist and function as monomeric units that interact with the corresponding G proteins. Recent studies have revealed the ability of GPCRs to form oligomers and it is now widely accepted that homo and heterodimerization may represent an additional mechanism for regulating GPCR-mediated signaling. Through the combined application of biochemical and biophysical approaches, it was recently demonstrated that 5-HT_7 receptors can form heterodimers with 5-HT_{1A} receptors both *in vitro* and *in vivo* [6]. The effects mediated by $G\alpha_s$ proteins are in the section above. The summary of the signaling processes mediated by $G\alpha_{12/13}$ is shown in the section below.

Table S1. 5-HT receptors and their subtypes: central signaling and signal transduction systems.

Receptor	Structure	Main signaling system	Main transduction system
5-HT_{1A}	GPCR	$G\alpha_{i/o}$	Blockade of AC activity with consequent reduction in cAMP synthesis
5-HT_{1B}	GPCR	$G\alpha_{i/o}$	Blockade of AC activity with consequent reduction in cAMP synthesis
5-HT_{1D}	GPCR	$G\alpha_{i/o}$	Blockade of AC activity with a consequent reduction in cAMP synthesis
5-ht_{1e}	GPCR	$G\alpha_{i/o}$	Blockade of AC activity with consequent reduction in cAMP synthesis
5-HT_{1F}	GPCR	$G\alpha_{i/o}$	Blockade of AC activity with consequent reduction in cAMP synthesis
5-HT_{2A}	GPCR	$G\alpha_{q/11}$	PLC activation with consequent increase in the synthesis of IP3 and DAG
5-HT_{2B}	GPCR	$G\alpha_{q/11}$	PLC activation with a consequent increase in the synthesis of IP3 and DAG
5-HT_{2C}	GPCR	$G\alpha_{q/11}$	PLC activation with consequent increase in the synthesis of IP3 and DAG
5-HT_{3A}	Ion channel		Ionic conductance (K^+, Na^+ and Ca^{2+})
5-HT_{3B}	Ion channel		Ionic conductance (K^+, Na^+ and Ca^{2+})
5-HT_{3C}	Ion channel		Ionic conductance (K^+, Na^+ and Ca^{2+})
5-HT_{3D}	Ion channel		Ionic conductance (K^+, Na^+ and Ca^{2+})
5-HT_{3E}	Ion channel		Ionic conductance (K^+, Na^+ and Ca^{2+})
5-HT_4	GPCR	$G\alpha_s$	Stimulation of AC activity with consequent increase in cAMP synthesis
5-HT_{5A}	GPCR	$G\alpha_{i/o}$	Blockade of AC activity with consequent reduction in cAMP synthesis.
5-ht_{5b}	GPCR	$G\alpha_{i/o}$	Blockade of AC activity with consequent reduction in cAMP synthesis.
5-HT_6	GPCR	$G\alpha_s$	Stimulation of AC activity with consequent increase in cAMP synthesis
5-HT_7	GPCR	$G\alpha_s$	Stimulation of AC activity with consequent increase in cAMP synthesis.

Abbreviations: AC = Adenylate cyclase, cAMP = Adenosine 3′,5′-cyclic monophosphate, Ca^{2+} = Calcium, DAG = Diacylglycerol, GPCR = G protein-coupled receptors, K^+ = Potassium, Na^+ = Sodium, IP3 = Inositol 1,4,5-triphosphate, PLC = Phospholipase C.

Table S2. Phytochemicals submitted to clinical trials to verify their antidepressant and/or anxiolytic effectiveness.

Compound	Informed Source	Disorder/Symptom	Diagnostic Instruments	Administration	Major findings	References
Crocin	*Crocus sativus* stigmas	Major depressive disorder	Beck depression inventory and beck anxiety inventory	4 weeks with Crocin (30 mg/day; 15 mg twice a day) + one SSRI (fluoxetine 20 mg/day or sertraline 50 mg/day or citalopram 20 mg/day)	Crocin as an adjunct to SSRIs leads to significantly greater decrease in depressive symptoms in comparison with SSRI alone treatment	[7]
Crocin	*Crocus sativus* stigmas	Depression in subjects with metabolic syndrome	Beck depression inventory	8 weeks with Crocin (30 mg/day; 15 mg twice a day)	Reduction in depression symptoms	[8]
Crocin	*Crocus sativus* stigmas	Depression and anxiety in patients under methadone maintenance treatment	Beck Depression Inventory and Beck Anxiety Inventory	8 weeks with Crocin (30 mg/day; 15 mg twice a day)	Reduction in depression and anxiety symptoms	[9,10]
Crocin	*Crocus sativus* stigmas	Depression in Coronary Artery Disease Patients	Beck depression inventory-II	8 weeks with Crocin (30 mg/day)	Reduction in depression symptoms	[11]
Curcumin	Not informed	Major depressive disorder	Hamilton Depression Rating Scale and Montgomery-Asberg Depression Rating Scale	6 weeks with escitalopram plus curcumin (1000 mg/day)	Administration of curcumin achieved superior response outcomes as compared to single antidepressant treatment	[12]
Curcumin	Not informed	Major depressive disorder	Montgomery-Asberg Depression Rating Scale and	12 weeks with antidepressive drug therapy plus curcumin (500–1500 mg/day; At baseline: 250 mg/ twice a day for 1 week. After this,	Adjunctive curcumin has significant antidepressant effects. On Hamilton Anxiety	[13]

			Hamilton Anxiety Rating Scale	the dose was increased every week by 250 mg/day. Thus, after 4 weeks, patients were taking 1500 mg curcumin/day.	Rating Scale, curcumin decreased scores more than placebo in males, whereas in females, curcumin increased this rating scale scores more than placebo.	
Curcumin	Not informed	Depression and anxiety in diabetic patients with peripheral neuropathy:	Depression, Anxiety, Stress Scale	8 weeks with nano-curcumin (80 mg/daily)	Nano-curcumin was effective in reducing depression and anxiety scores in patients with diabetic polyneuropathy	[14]
Curcumin	Not informed	Hot flashes and anxiety in postmenopausal women	Spielberger State-Trait Anxiety Inventory	8 weeks with curcumin (1000 mg/day; 500mg twice a day)	Curcumin reduced hot flashes without significant effect on anxiety symptoms	[15]
L-theanine	Not informed	Major depressive disorder	Hamilton Depression Rating Scale and State-Trait Anxiety Inventory	8 weeks with L-theanine (250 mg/day) + current medication of each participant	Effective in reducing depression scores, a tendency of reduction in anxiety-state and a significant reduction in anxiety-trait scores	[16]
L-theanine	Enzymatic synthetic product	Stress-Related Symptoms (Depression and State-Trait Anxiety) in Healthy Adults	Self-rating Depression Scale and State-Trait Anxiety Inventory	4 weeks with L-theanine (200 mg/day)	Self-rating Depression Scale and State-Trait Anxiety Inventory-trait scores were significantly reduced	[17]
L-theanine	Not informed	Generalized Anxiety Disorder	Hamilton Anxiety Rating Scale	8 weeks with L-theanine (450-900 mg/day) + antidepressant treatment	No change in anxiety was found	[18]

Valeric acid	Not informed	Anxiety in Women Undergoing Hysterosalpingography	Visual Analog Anxiety Scale	1500 mg valeric tablet 90 minutes before intervention	Anxiolytic effectiveness	[19]

Abbreviations: SSRI = Selective serotonin reuptake inhibitors.

Table S3. Herbal medicines submitted to clinical trials to verify their antidepressant and/or anxiolytic effectiveness.

Plant	Marker Compounds	Disorder/Symptom	Diagnostic Instruments	Administration	Major findings	References
Aloysia polystachya	Acteoside	Mild-to-moderate anxiety	Hamilton Anxiety Ranking Scale	8 weeks with *A. polystachya* leaves dried hydroethanolic extract (600 mg/day; 300 mg twice a day; acteoside 115.7 ± 1.9 ìg/mg of dried extract)	Anxiolytic effectiveness	[20]
Camellia sinensis	Theanine	Stress response in students	State-Trait Anxiety Inventory	17 days with intake of low-caffeine tea (theanine 15 mg/day)	The subjective stress of students was significantly lower	[21]
Caralluma fimbriata	Not informed	Anxiety and stress in healthy adults	Generalized anxiety disorder 7-item and Perceived stress scale	8 weeks with C. fimbriata (1000 mg/day; 500 mg twice a day)	Anxiolytic effectiveness and lower stress score	[22]
Citrus aurantium	Limonene	Anxiety in the moment that precedes the collection of medullary material in patients with chronic myeloid leukemia	State-Trait Anxiety Inventory	10 ml of C. aurantium essential oil (98% limonene) diffused in the room through electric dispenser	Anxiolytic effectiveness	[23]
Citrus aurantium	Not informed	Anxiety during colonoscopy	Numeric Rating Scale	Aromatherapy using 0.30 mL of C. aurantium (grapefruit) essential oil during colonoscopy	Anxiety was significantly attenuated	[24]
Citrus aurantium	Limonene, linalool, flavonoids	Anxiety in postmenopausal women	Spielberger's State-Trait Anxiety Inventory	8 weeks with C. aurantium flower powder 1000 mg/day; 500 mg twice a day;	Anxiolytic effectiveness in postmenopausal women	[25]

				Limonene (20%), linalool (32%), flavonoid (5%)		
Citrus bergamia	Linalool and limonene	Medical Office–Induced Anxiety Among Children with an Autism Spectrum Disorder	State-Trait Anxiety Inventory for Children	15-minute inhalation of C. bergamia essential oil	There was no significant difference	[26]
Coptis chinensis + *Cinnamomum cassia*	Not informed	Depression	Hamilton Depression Rating Scale	8 weeks with 15 g *C. chinensis* Franch and 2 g *C. cassia* Presl (of crude herbs) plus fluoxetine 20 mg once daily	Results were not reported	
Crocus sativus	Not informed	Generalized anxiety disorder	Hamilton anxiety rating scale	6 weeks with saffron (450 mg) + sertraline (50 mg)/daily	Anxiolytic effectiveness	[27]
Crocus sativus	Not informed	Mild-to-moderate mixed anxiety and depression	Beck depression inventory and Beck anxiety inventory	12 weeks with dried saffron stigma (100 mg/day; 50 mg twice a day)	Effectiveness for mild-to-moderate mixed anxiety and depression	[28]
Crocus (SaffroMood®)	Crocin	Major Depressive Disorder with Anxious Distress	Hamilton Depression Rating Scale and Hamilton Anxiety Rating Scale	6 weeks with saffron (30 mg/day; 15 mg twice a day -each capsule had 1.65–1.75 mg crocin)	Antidepressant and anxiolytic effectiveness	[29]
Crocus sativus (affron®)	Lepticrosalides®	Anxiety and depressive symptoms in adolescents	Revised Child Anxiety and Depression Scale	8 weeks with standardized (to contain > 3.5% Lepticrosalides®) saffron stigmas extract (affron®) (28 mg/day; 14 mg twice a day)	Based on youth self-reports, affron® was associated with greater improvements in separation anxiety, social phobia and	[30]

					depression symptoms	
Crocus sativus	Crocin	Mild-to-moderate postpartum depression	Beck depression inventory-II	8 weeks with saffron stigma (30 mg/day; 15 mg twice a day - 5 ± 0.25 mg of crocin)	Antidepressant effectiveness	[31]
Crocus sativus (SaffroMood®)	Crocin	Mild-to-moderate postpartum depression	Hamilton Depression Rating Scale	6 weeks with saffron (30 mg/day; 15 mg twice a day -each capsule had 1.65–1.75 mg crocin)	40.60% patients in the saffron group experienced complete response	[32]
Crocus sativus stigmas (affron®)	Lepticrosalides®	Persistent depression	Montgomery-Åsberg Depression Rating Scale	8 weeks with standardized (to contain > 3.5% Lepticrosalides®) saffron extract (affron®) (28 mg/day; 14 mg twice a day) + one antidepressant	Greater improvement in depressive symptoms as measured by the clinician	[33]
Crocus sativus	Not informed	Major depressive disorder	Beck depression inventory	4 weeks with the saffron powder (30 mg/day) + fluoxetine (20 mg/day)	Saffron as an adjunct to fluoxetine did not lead to significantly greater decrease in depressive symptoms in comparison with fluoxetine alone	[34]
Crocus sativus	Not informed	Major depressive disorder	Beck depression inventory	4 weeks with the saffron stigma powder (30 mg/day) + fluoxetine (20 mg/day)	Saffron as an adjunct to fluoxetine did not lead to significantly greater decrease in	[35]

					depressive symptoms in comparison with fluoxetine alone	
Crocus sativus	Not informed	Major depressive disorder in older people	Hamilton Depression Rating Scale	6 weeks with saffron (60 mg/day)	Reduction in depression symptoms	[36]
Crocus sativus (SaffroMood®)	Crocin	Major depressive disorder associated with post-menopausal hot flashes	Hamilton Depression Rating Scale	6 weeks with saffron stigma (30 mg/day; 15 mg twice a day - each capsule had 1.65–1.75 mg crocin)	Saffron is effective in improving depressive symptoms in post-menopausal healthy women	[37]
Crocus sativus	Not informed	Depression among recovered consumers of methamphetamine living with HIV/AIDS	Beck depression inventory-II	8 weeks with saffron ethanolic extract (30 ml/day)	Reduction in depression symptoms	[38]
Crocus sativus	Not informed	Depression in Coronary Artery Disease Patients	Beck depression inventory-II	8 weeks with saffron stigma aqueous extract (30 mg/day)	Reduction in depression symptoms	[11]
Crocus sativus	Not informed	Depression, and anxiety in patients undergoing coronary artery bypass grafting	Mini Mental Status Examination and subscales of Hospital Anxiety and Depression Scale	12 weeks with saffron (30 mg/day; 15 mg twice a day)	No significant difference was detected between saffron and placebo groups	[39]
Crocus sativus	Not informed	Mild to moderate comorbid depression-anxiety in patients with type 2 diabetes mellitus	Hamilton Depression Rating Scale and Hamilton Anxiety Rating Scale	8 weeks with saffron hydro-alcoholic extract (30 mg/day; 15 mg twice a day)	Reduction in anxiety and depression + anxiety scores	[40]

Curcuma longa (rhizomes) (BCM-95®) + *Crocussativus* (stigmas) (affron®)	BCM-95® containing total curcuminoids 88% (curcumin, bisdemethoxycurcumin, demethoxycurcumin) and volatile oils 7% + saffron standardised to contain > 3.5% Lepticrosalides®	Major depressive disorder	Inventory of Depressive Symptomatology self-rated version Inventory and Spielberger State-Trait Anxiety Inventory	12 weeks with BCM-95® (250 mg or 500 mg) or BCM-95® (250 mg) + saffron (15 mg)/twice a day	Antidepressant and anxiolytic effects. Saffron did not improve effectiveness.	[41]
Curcuma longa (BCM-95®)	curcumin, bisdemethoxycurcumi, demethoxycurcumin and volatile oils	Major depressive disorder	Inventory of Depressive Symptomatology self-rated version	8 weeks with C. longa (rhizomes) BCM-95® (1000 mg/day; 500 mg twice a day)	Reduction in depression symptoms	[42]
Curcuma longa (C3 Complex®) + piperine (Bioperine®)	Standardized for minimum 95% Curcuminoids	Major depressive disorder	Hospital Anxiety and Depression Scale and Beck Depression Inventory-II	8 weeks with antidepressive drug therapy plus C. longa (rhizomes) (C3 Complex® - 1000 mg) + piperine (Bioperine® - 10 mg)/day	Curcuminoids–piperine significantly improves the efficacy of standard therapy	[43]
Curcuma longa (C3 Complex®) + piperine (Bioperine®)	Standardized for minimum 95% Curcuminoids	Anxiety and Depression in Obese Individuals	Beck depression inventory and Beck anxiety inventory	30 days with C. longa (rhizomes) (C3 Complex® - 1000 mg) + piperine (Bioperine® - 10 mg)/day	Potential anti-anxiety effect without significant impact on Beck depression inventory scores	[44]
Curcuma xanthorrhiza	Not informed	Depression in systemic lupus erythematosus patient	Beck depression inventory	4 weeks with *C. xanthorrhiza* extract containing 50mg of curcuminoids (150 mg/day; 50 mg 3 times each day)	Beck depression inventory score were not significantly lower	[45]

					than before treatment (p=0.059)	
Cuscutaplaniflora	Not informed	Major depressive disorder	Beck Depression Inventory and Hamilton Depression Rating Scale	8 weeks with *C. planiflora* (2000 mg/day; 500 mg 4 times each day) + conventional drugs	C. planiflora aqueous extract as an adjunct to conventional drugs led to significantly greater decrease in depressive symptoms in comparison with conventional drugs	[46]
Echinacea angustifolia (AnxioCalm® in the US)	*Echinacea alkamides*	High anxiety	State-Trait Anxiety Inventory and Beck Depression Inventory	7 days with *Echinacea* (80 mg/day; 40 mg twice a day - 1-1.5% *Echinacea alkamides*)	Decreased state anxiety in subjects. The extract did not affect Beck Depression Inventory score	[47]
Echiumamoenum + *Melissa officinalis* + *Crocussativus*	Not informed	Major depressive disorder	Hamilton Depression Rating Scale	8 weeks with 3 g of *E. amoenum* flowers + 1.5 g *M. officinalis* leaves + 150 mg *C. sativus* stigma	Hamilton Depression Rating score were significantly lower	[48]
Elaeagnus angustifolia	Not informed	Anxiety resulting from sexual dysfunction in women	Spielberger's State-Trait Anxiety Inventory	35-days with E. angustifolia flowers (4.5 g/day; 2.25 g/twice a day)	No significant differences were observed	[49]
Foeniculum vulgare	Anethole	Depression and anxiety in postmenopausal women	Anxiety and Depression Scale and Zung's Self Rating Depression Scale	90 days with *F. vulgare* (300 mg/day; 100 mg 3 times each day - each capsule was standardized to 21–27 mg anethole)	No significant differences were observed	[50]

Galphimia glauca	Galphimine B (G-B)	Generalized anxiety disorder	Hamilton anxiety rating scale	10 weeks with the G-B standardized extract (0.374 mg/once a day)	Anxiolytic effectiveness	[51]
Galphimia glauca	Galphimine B (G-B)	Social anxiety disorder	Brief social phobia scale	10 weeks with the G-B standardized extract (0.374 mg/once a day)	Anxiolytic effectiveness	[52]
Ginkgo biloba	Not informed	Depression in elderly patients	Hamilton Depression Rating Scale	12 weeks with *G. biloba* extract (57.6 mg/day; 19.2 mg three times a day) + Citalopram (20 mg/day, once a day)	As an adjunctive treatment, effectively improve depressive symptoms	[53]
Geranium spp	Not informed	Anxiety in patients with acute myocardial infarction	State-Trait Anxiety Inventory	Aromatherapy (three drops of Geranium essential oil) was performed for 20 minutes twice daily (10-11 a.m. and 6-7 p.m.) on two consecutive days	Geranium aroma caused significantly greater reductions in anxiety scores	[54]
Gynostemma pentaphyllum	Ombuoside	Anxiety in healthy subjects under chronic stressful conditions	State-Trait Anxiety Inventory, Hamilton Anxiety Inventory and Beck Anxiety Inventory	8 weeks with *G. pentaphyllum* (leaves) (400 mg/day; 200 mg/twice a day - ombuoside 1.4 to 2.1 mg/g of extract)	Lowered the Trait Anxiety Scale score. No significant differences the other scales	[55]
Humulus lupulus (Melcalin® HOPs)	Not informed	Self-reported depression, anxiety and stress in healthy young adults	Depression Anxiety Stress Scale-21	4 weeks with H. lupulus (inflorescences) (Melcalin® HOPs) (400 mg/day; once a day)	Greater decrease in depressive, anxiety, and stress symptoms in comparison with placebo	[56]
Hypericum perforatum extract WS® 5570	Not informed	Moderate depression	Hamilton Depression Rating Scale	6 weeks with Hypericum extract WS® 5570 (900 mg/day; 300 mg 3 times each day)	Patients treated with WS® 5570 responded to	[57]

					treatment and more patients showed remission compared with the reference group (paroxetine)	
*Hypericum perforatum*Nervaxon® or IperiPlex®	Hypericin	Moderate depression	Zung Self-Rating Depression Scale	3 months of treatment + 3 months of wash-out + 3 months of treatment + 3 months of wash-out with Nervaxon® (monofractionated extract) or IperiPlex® (multi-fractionated extract) (both 600 mg/day; 300 mg twice a day - both containing 0.3% hypericin)	Nervaxon® demonstrated no efficacy after 6 months and partial efficacy after 12 months. Treatment with IperiPlex® demonstrated highly significant results at both 6 and 12 months	[58]
Hypericum perforatum	Not informed	Postmenopausal symptoms and depression	Hamilton Depression Rating Scale	8 weeks with *Hypericum* extract (0.990 mg/day; 0.330 mg 3 times each day)	Reduced hot flashes, menopausal symptoms, and depression in postmenopausal women	[59]
Lavandula spp.	Not informed	Anxiety of patients having bone marrow biopsy	Visual Anxiety Scale	3 drops of 10% lavender essential oil for 15 min before the biopsy	Anxiolytic effectiveness	[60]
Lavandula spp	Not informed	Anxiety of patients in coronary intensive care units	Beck Anxiety Inventory	2% lavender essential oil via inhalation during 20 min for 15 days	Anxiolytic effectiveness	[61]

Lavandula spp	Not informed	Anxiety during colonoscopy	Numeric Rating Scale	Aromatherapy using 0.05 mL of lavender essential oil during colonoscopy	No significant change was observed	[24]
Lavandula spp	Not informed	Anxiety during gynecological examination	Spielberger's State Anxiety Inventory	10 to 15 minutes for inhalation of lavender essential oil	Lavender scent reduced anxiety during gynecological examination	[62]
Lavandula angustifolia	Not informed	Anxiety associated with the peripheral venous cannulation in patients undergoing surgery	Anxiety visual analog scale	Two drops of 1% lavender essential oil inhalation for 5 min	Anxiety scores in the lavender group were significantly lower	[63]
Lavandula angustifolia	Linalool, linalyl acetate, caryophyllene	Anxiety in postmenopausal women	Spielberger's State-Trait Anxiety Inventory	*L. angustifolia* flower powder 1000 mg/day; 500 mg twice a day - Linalool (36.12%) linalyl acetate (26.32%), caryophyllene (7.55%)	Anxiolytic effectiveness in postmenopausal women	[25]
Lavandula angustifolia (Silexan®)	Linalool and linalyl acetate	Anxiety-related restlessness and disturbed sleep	Hamilton Anxiety Rating Scale and Zung Self-rating Anxiety Scale	10 weeks with 80 mg standardized *L. angustifolia* flowers (36.8% of linalool and 34.2% of linalyl acetate) essential oil (Silexan®) once daily	Calming and anxiolytic efficacy of Silexan	[64]
Lavandula angustifolia (Silexan®)	Linalool and linalyl acetate	Mixed anxiety-depression	Hamilton Anxiety Rating Scale and Montgomery Åsberg Depression Rating Scale	70 days with 80 mg standardized *L. angustifolia* flowers (36.8% of linalool and 34.2% of linalyl acetate) essential oil (Silexan®) once daily	Anxiolytic and antidepressant effectiveness	[65]

Lavandula angustifolia (Silexan®)	Linalool and linalyl acetate	Generalized anxiety disorder	Hamilton Anxiety Rating Scale and Covi Anxiety Scale	10 weeks with daily doses of 10, 40, 80, and 160 mg *L. angustifolia* flowers standardized (36.8% of linalool and 34.2% of linalyl acetate) essential oil (Silexan®)	Silexan 160 mg/day is efficacious whereas 80 mg/day may represent the lower end of the therapeutic range	[66]
Lavandula angustifolia	Not informed	Anxiety of patients undergoing oral surgery	Dental Anxiety Questionnaire, Modified Dental Anxiety Scale and State-Trait Anxiety Inventory–State Scale	Inhalation of 100% pure, high-strength lavender oil in a separate room for 3 min prior to surgery	Inhalation of lavender oil reduces perioperative anxiety	[67]
Lavandula angustifolia	Not informed	Anxiety in women before undergoing breast surgery	The State-Trait Anxiety Inventory	Two drops of 2% *L. angustifolia* essential oil inhalation for 10 minutes	Slight but statistically significant increase in positive feelings	[68]
Lavandulaangustifolia + Citrus sinensis + Citrus bergamia	Not informed	Geriatric depression	Geriatric Depression Scale Short Form	8 weeks with aromatherapy massage with L. angustifolia, *C. sinensis* and *C. bergamia* (in a 2:1:1 ratio) essential oils for 30 min, twice weekly	Antidepressant effectiveness in older adults	[69]
Lavandula hybrida	Not informed	Anxiety in patients treated with chemotherapy	The State-Trait Anxiety Inventory	Three drops of lavender essential oil during chemotherapy and at home, every night, for five minutes for one month	Trait anxiety was reduced	[70]
Lavandula hybrida	Not informed	Anxiety in patients undergoing colorectal surgery	State Anxiety Inventory	5% lavender essential oil for ten minutes before surgery	Reduced the level of anxiety in patients with colorectal surgery	[71]

Lepidium meyenii	Not informed	Depression in postmenopausal women	Greene Climacteric Scale and Women's Health Questionnaire	6 weeks with *L. meyenii* 3.3 g/day: four capsules (462 mg/ capsule) following breakfast and three following dinner	Anxiolytic and antidepressant effectiveness in postmenopausal women	[72]
Matricaria chamomilla	Apigenin	Moderate to severe generalized anxiety disorder	GAD-7 item, Beck Anxiety Inventory and Hamilton Anxiety Rating Scale	8 weeks with fixed-flexible dosing strategy with *M. chamomilla* flowers 500 up to 1,500 mg/day (Chamomile 500 mg capsules containing 1.2% apigenin)	Anxiolytic effectiveness	[73]
Matricaria chamomilla	Apigenin-7-glycosides	Generalized anxiety disorder	Clinical Global Impression-Severity, GAD-7 item, Beck Anxiety Inventory and Hamilton Anxiety Rating Scale	38 weeks: after 12 weeks of open-label therapy with chamomile flowers, treatment responders were randomized to receive 26 weeks of chamomile continuation. Participants were treated with *M. chamomilla* flowers 1500 mg/day (500 mg three times a day - 6 mg of apigenin-7-glycosides)	Long-term chamomile was safe and significantly reduced moderate-to-severe GAD symptoms, but did not significantly reduce relapse rate	[74]
Matricaria spp. + *Lavandulaspp*	Not informed	Anxiety of Clinical Nurses	Beck Anxiety Inventory	1.5% Matricaria spp. + Lavandula spp (chamomile-lavender) essential oil for 20 min per shift, during three consecutive shifts and at employees' rest time	Anxiolytic effectiveness	[75]
Melissa officinalis	Not informed	Anxiety	General Health Questionnaire-28	14 day with lyophilized aqueous extract of M. officinalis leaves (1000 mg/day; 500 mg twice a day)	Number of the patients with anxiety and insomnia disorder	[76]

					decreased significantly	
Melissa officinalis	Not informed	Anxiety in patients with chronic stable angina	Depression, anxiety, and stress scale e shortened 21- item version	8 weeks with *M. officinalis* shoot powder (3 g/day; 1 g three times per day)	Anxiolytic effectiveness in chronic stable angina patients	[77]
Mentha piperita	Not informed	Anxiety in healthy adults	State-Trait Anxiety Inventory	Participants received two capsules containing 100 or 50 ìL of M. piperita essential oil	No significant differences on mood measures	[78]
Nepeta menthoides	Not informed	Major depressive disorder	Beck Depression Inventory-II	5 days with 400 mg/day *N. menthoides* aqueous extract + up to 4 weeks with 800 mg/day N. menthoides aqueous extract	Reduction in depression symptoms	[79]
Nepeta menthoides	Not informed	Major depressive disorder	Beck Depression Inventory and Hamilton Depression Rating Scale	8 weeks with *N. menthoides* aqueous extract (800 mg/day; 400 mg twice a day) + conventional drugs	*N. menthoides* aqueous extract as an adjunct to conventional drugs lead to significantly greater decrease in depressive symptoms in comparison with conventional drugs	[46]
Osmanthus fragrans	Not informed	anxiety during colonoscopy	Numeric Rating Scale	Aromatherapy using 0.05 mL of *O. fragrans* essential oil during colonoscopy	Anxiety was significantly attenuated	[24]
Panax ginseng	Ginsenosides, such as Rg1, Rb1, Rg3s, Re, Rc, Rb2, Rd, Rf, Rh1, Rg2s	Major depressive disorder	Montgomery-Åsberg Depression Rating Scale, Depression	4 weeks with *P. ginseng* (2000 mg/day + usual antidepressant treatment and	Decrease in depressive symptoms in the	[80]

			Residual Symptom Scale and Depression and Somatic Symptom Scale	4 weeks with *P. ginseng* (3000 mg/day + usual antidepressant treatment	three diagnostic instruments	
Passiflora incarnata	Not informed	Anxiety in patients undergoing tooth extraction	Corah Dental Anxiety Scale	*P. incarnata* (260 mg) orally administered 30 min before surgery	*P. incarnata* has an anxiolytic effect	[81]
Piper methysticum	Kavalactones	Generalized anxiety disorder	Hamilton Anxiety Rating Scale	16 weeks with *P. methysticum* aqueous extract (240 mg of Kavalactones per day; 120 mg twice a day).	Results were not reported	[82]
Rosa damascena	linalool, nerol, geranio, 1-nonadecene, n-tricosane, hexatriacontane and n-pentacosane	Anxiety and pain of labor during first stage of labor	Spielberger anxiety questionnaire and numerical pain rating scale	0.08 mL of R. damascena essential oil every 30 min.	Aromatherapy results in lower anxiety and pain levels	[83]
Rosa spp.	Not informed	Anxiety of patients undergoing coronary artery bypass graft surgery	Spielberger's Anxiety Inventory	Three drops of 4% rose essential oil for 10 minutes one night and one hour before surgery	No significant differences in state anxiety, trait anxiety, and total anxiety	[84]
Rosa spp.	Not informed	Pain anxiety in burn patients	Burn specific pain anxiety scale	Five drops of 40% rose essential oil for 20 min for three consecutive days	Inhalation of rose aroma reduced pain anxiety	[85]
Rhodiola rosea	Rosavin, rhodioloside	Mild to moderate major depressive disorder	Hamilton Depression Rating and Beck Depression Inventory	*R. rosea* SHR-5 (rosavin 3.07% rhodioloside 1.95%) powdered extract (340) mg was administered in a dose-escalation way (one up to four capsules daily) for 12 weeks	*R. rosea* may present modest antidepressant effects	[86]

Rhodiola rosea (Vitano®)	Rosalin®	Self-reported anxiety, stress, and other mood symptoms	Spielberger State–Trait Anxiety Inventory, Perceived Stress Scale and Profile of Mood States Inventory	14-days with *R. rosea* (400 mg/day; 200 mg of Vitano® twice a day)	Reduction in self-reported, anxiety, stress, and depression symptoms	[87]
Withaniasomnifera	Withanolide glycosides	Anxiety in stressed, healthy adults	Hamilton Anxiety Rating Scale and Depression, Anxiety and Stress Scale -21	60-days with *W. somnifera* extract (240 mg/day; 240 mg/once a day – 84 mg with anolide glycosides/capsule)	Greater reduction in the Hamilton Anxiety Rating Scale, although no statistical significance changes in the DASS-21	[88]
Withania somnifera	Not informed	Generalized anxiety disorder	Hamilton Anxiety Rating Scale	6 weeks with *W. somnifera* root extract (1 g/day; 1 g/once a day)	Effective adjunctive therapy to SSRIs	[89]

Abbreviations: SSRIs = Selective Serotonin Reuptake Inhibitors.

1.

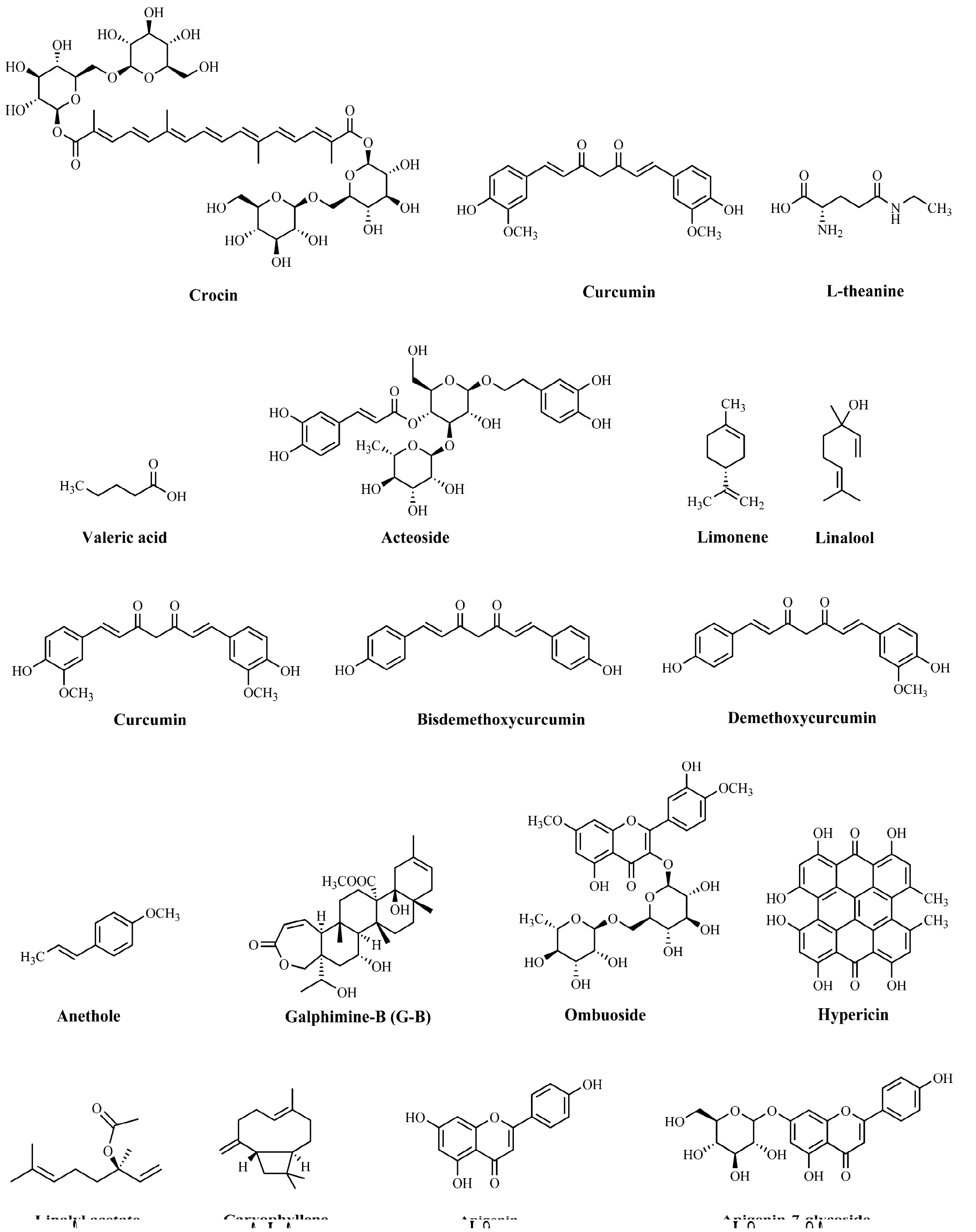

Figure S8.*Cont.*

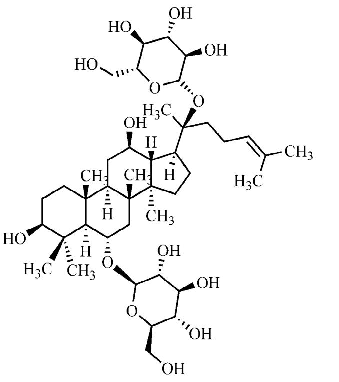

Ginsenoside Rg1

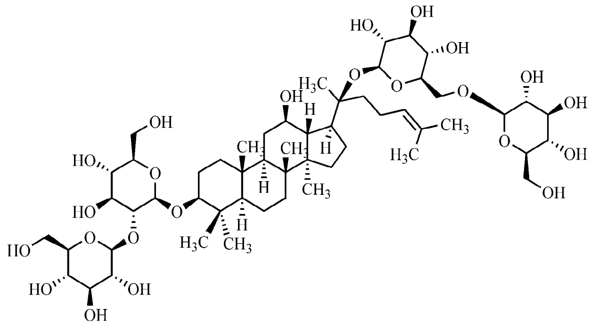

Ginsenoside Rb1

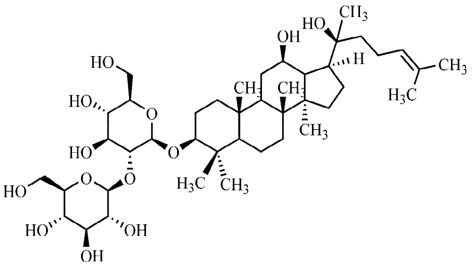

Ginsenoside Rg3s

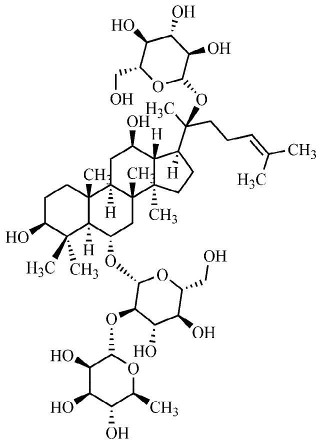

Ginsenoside Re

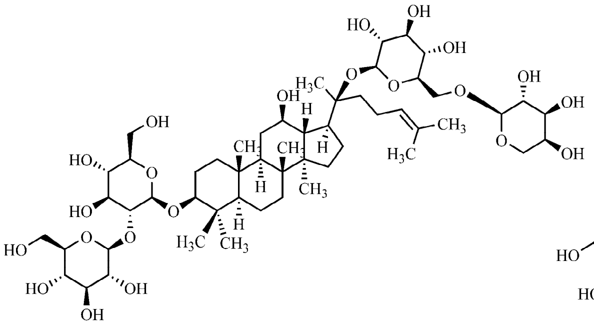

[illegible]

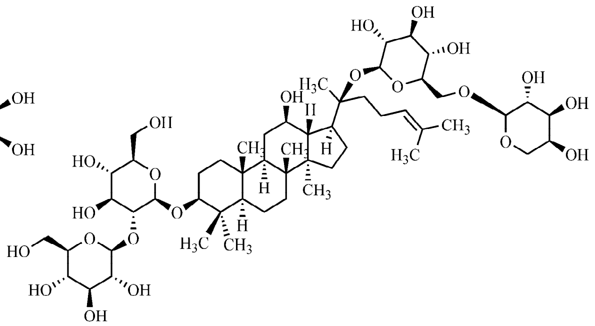

[illegible]

Figure S8. *Cont.*

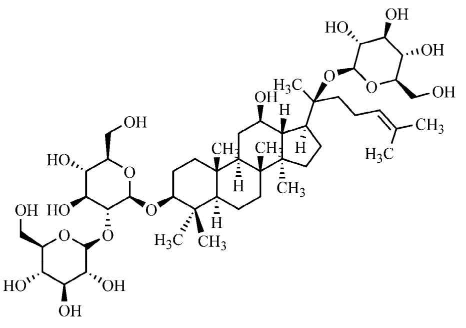

Ginsenoside Rd

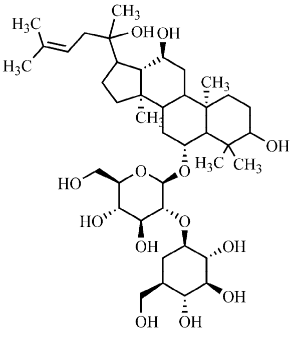

Ginsenoside Rf

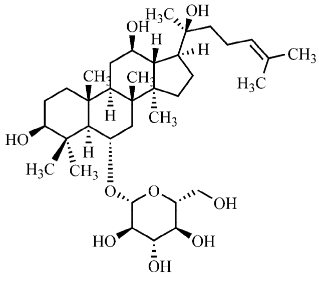

Ginsenoside Rh1

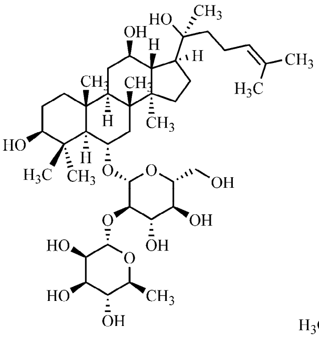

Ginsenoside Rg2s

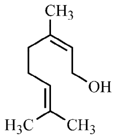

Nerol

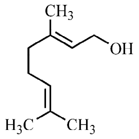

Geraniol

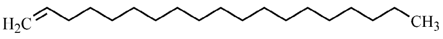

1-nonadecene

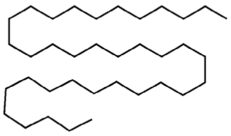

Hexatriacontane

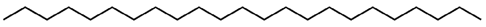

n-tricosane

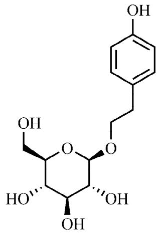

Rhodioloside

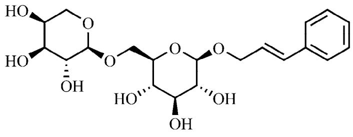

Rosavin

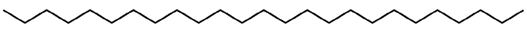

n-pentacosane

Figure S8. Structural formulas of phytochemicals cited in tables 4 and 5.

References

1. Stahl, S.M. Enhancing Outcomes from Major Depression: Using Antidepressant Combination Therapies with Multifunctional Pharmacologic Mechanisms from the Initiation of Treatment. *CNS Spectr.* **2010**, *15*, 79–94, doi:10.1017/S1092852900027334.
2. Yu, Y.; Zhu, W.; Liang, Q.; LIU, J.; Yang, X.; Sun, G. Tropisetron attenuates lipopolysaccharide induced neuroinflammation by inhibiting NF-κB and SP/NK1R signaling pathway. *J. Neuroimmunol.* **2018**, *320*, 80–86, doi:10.1016/j.jneuroim.2018.05.001.
3. Richter, D.W.; Manzke, T.; Wilken, B.; Ponimaskin, E. Serotonin receptors: guardians of stable breathing. *Trends Mol. Med.* **2003**, *9*, 542–548, doi:10.1016/j.molmed.2003.10.010.
4. Hilenski, L.L.; Griendling, K.K. Vascular Smooth Muscle. In *Vascular Medicine: A Companion to Braunwald's Heart Disease*; Elsevier, 2013; pp. 25–42 ISBN 9781437729306.
5. Masson, J.; Emerit, M.B.; Hamon, M.; Darmon, M. Serotonergic signaling: multiple effectors and pleiotropic effects. *Wiley Interdiscip. Rev. Membr. Transp. Signal.* **2012**, *1*, 685–713, doi:10.1002/wmts.50.
6. Renner, U.; Zeug, A.; Woehler, A.; Niebert, M.; Dityatev, A.; Dityatev, G.; Gorinski, N.; Guseva, D.; Abdel-Galil, D.; Fröhlich, M.; et al. Heterodimerization of serotonin receptors 5-HT1A and 5-HT7 differentially regulates receptor signalling and trafficking. *J. Cell Sci.* **2012**, *125*, 2486–99, doi:10.1242/jcs.101337.
7. Talaei, A.; Hassanpour Moghadam, M.; Sajadi Tabassi, S.A.; Mohajeri, S.A. Crocin, the main active saffron constituent, as an adjunctive treatment in major depressive disorder: A randomized, double-blind, placebo-controlled, pilot clinical trial. *J. Affect. Disord.* **2015**, *174*, 51–56, doi:10.1016/j.jad.2014.11.035.
8. Nikbakht Jam, I.; Sahebkar, A.H.; Eslami, S.; Mokhber, N.; Nosrati, M.; Khademi, M.; Foroutan-Tanha, M.; Ghayour-Mobarhan, M.; Hadizadeh, F.; Ferns, G.; et al. The effects of crocin on the symptoms of depression in subjects with metabolic syndrome. *Adv. Clin. Exp. Med.* **2017**, *26*, 925–930, doi:10.17219/acem/62891.
9. Ghaderi, A.; Rasouli-Azad, M.; Vahed, N.; Banafshe, H.R.; Soleimani, A.; Omidi, A.; Ghoreishi, F.S.; Asemi, Z. Clinical and metabolic responses to crocin in patients under methadone maintenance treatment: A randomized clinical trial. *Phyther. Res.* **2019**, *33*, 2714–2725, doi:10.1002/ptr.6445.
10. Khalatbari-mohseni, A.; Banafshe, H.R.; Mirhosseini, N.; Asemi, Z.; Ghaderi, A.; Omidi, A. The effects of crocin on psychological parameters in patients under methadone maintenance treatment: a randomized clinical trial. *Subst. Abuse Treat. Prev. Policy* **2019**, *14*, 9, doi:10.1186/s13011-019-0198-1.
11. Abedimanesh, N.; Ostadrahimi, A.; Bathaie, S.Z.; Abedimanesh, S.; Motlagh, B.; Asghari Jafarabadi, M.; Taban Sadeghi, M. Effects of Saffron Aqueous Extract and Its Main Constituent, Crocin, on Health-Related Quality of Life, Depression, and Sexual Desire in Coronary Artery Disease Patients: A Double-Blind, Placebo-Controlled, Randomized Clinical Trial. *Iran. Red Crescent Med. J.* **2017**, *19*, e13676, doi:10.5812/ircmj.13676.
12. Yu, J.-J.; Pei, L.-B.; Zhang, Y.; Wen, Z.-Y.; Yang, J.-L. Chronic Supplementation of Curcumin Enhances the Efficacy of Antidepressants in Major Depressive Disorder. *J. Clin. Psychopharmacol.* **2015**, *35*, 406–410, doi:10.1097/JCP.0000000000000352.
13. Kanchanatawan, B.; Tangwongchai, S.; Sughondhabhirom, A.; Suppapitiporn, S.; Hemrunrojn, S.; Carvalho, A.F.; Maes, M. Add-on Treatment with Curcumin Has Antidepressive Effects in Thai Patients with Major Depression: Results of a Randomized Double-Blind Placebo-Controlled Study. *Neurotox. Res.* **2018**, *33*, 621–633, doi:10.1007/s12640-017-9860-4.
14. Asadi, S.; Gholami, M.S.; Siassi, F.; Qorbani, M.; Sotoudeh, G. Beneficial effects of nano-curcumin supplement on depression and anxiety in diabetic patients with peripheral neuropathy: A randomized, double-blind, placebo-controlled clinical trial. *Phyther. Res.* **2020**, *34*, 896–903, doi:10.1002/ptr.6571.
15. Ataei-Almanghadim, K.; Farshbaf-Khalili, A.; Ostadrahimi, A.R.; Shaseb, E.; Mirghafourvand, M. The effect of oral capsule of curcumin and vitamin E on the hot flashes and anxiety in postmenopausal women: A triple blind randomised controlled trial. *Complement. Ther. Med.* **2020**, *48*, 102267, doi:10.1016/j.ctim.2019.102267.
16. Hidese, S.; Ota, M.; Wakabayashi, C.; Noda, T.; Ozawa, H.; Okubo, T.; Kunugi, H. Effects of chronic <scp>l</scp> -theanine administration in patients with major depressive disorder: an open-label study. *Acta Neuropsychiatr.* **2017**, *29*, 72–79, doi:10.1017/neu.2016.33.

17. Hidese; Ogawa; Ota; Ishida; Yasukawa; Ozeki; Kunugi Effects of L-Theanine Administration on Stress-Related Symptoms and Cognitive Functions in Healthy Adults: A Randomized Controlled Trial. *Nutrients* **2019**, *11*, 2362, doi:10.3390/nu11102362.
18. Sarris, J.; Byrne, G.J.; Cribb, L.; Oliver, G.; Murphy, J.; Macdonald, P.; Nazareth, S.; Karamacoska, D.; Galea, S.; Short, A.; et al. L-theanine in the adjunctive treatment of generalized anxiety disorder: A double-blind, randomised, placebo-controlled trial. *J. Psychiatr. Res.* **2019**, *110*, 31–37, doi:10.1016/j.jpsychires.2018.12.014.
19. Gharib, M.; Samani, L.N.; Panah, Z.E.; Naseri, M.; Bahrani, N.; Kiani, K. The Effect of Valeric on Anxiety Severity in Women Undergoing Hysterosalpingography. *Glob. J. Health Sci.* **2015**, *7*, 358–363, doi:10.5539/gjhs.v7n3p358.
20. Carmona, F.; Coneglian, F.S.; Batista, P.A.; Aragon, D.C.; Angelucci, M.A.; Martinez, E.Z.; Pereira, A.M.S. Aloysia polystachya (Griseb.) Moldenke (Verbenaceae) powdered leaves are effective in treating anxiety symptoms: A phase-2, randomized, placebo-controlled clinical trial. *J. Ethnopharmacol.* **2019**, *242*, 112060, doi:10.1016/j.jep.2019.112060.
21. Unno, K.; Yamada, H.; Iguchi, K.; Ishida, H.; Iwao, Y.; Morita, A.; Nakamura, Y. Anti-stress Effect of Green Tea with Lowered Caffeine on Humans: A Pilot Study. *Biol. Pharm. Bull.* **2017**, *40*, 902–909, doi:10.1248/bpb.b17-00141.
22. Kell, G.; Rao, A.; Katsikitis, M. A randomised placebo controlled clinical trial on the efficacy of Caralluma fimbriata supplement for reducing anxiety and stress in healthy adults over eight weeks. *J. Affect. Disord.* **2019**, *246*, 619–626, doi:10.1016/j.jad.2018.12.062.
23. Pimenta, F.C.F.; Alves, M.F.; Pimenta, M.B.F.; Melo, S.A.L.; Almeida, A.A.F. de; Leite, J.R.; Pordeus, L.C. de M.; Diniz, M. de F.F.M.; Almeida, R.N. de Anxiolytic Effect of *Citrus aurantium* L. on Patients with Chronic Myeloid Leukemia. *Phyther. Res.* **2016**, *30*, 613–617, doi:10.1002/ptr.5566.
24. Hozumi, H.; Hasegawa, S.; Tsunenari, T.; Sanpei, N.; Arashina, Y.; Takahashi, K.; Konnno, A.; Chida, E.; Tomimatsu, S. Aromatherapies using Osmanthus fragrans oil and grapefruit oil are effective complementary treatments for anxious patients undergoing colonoscopy: A randomized controlled study. *Complement. Ther. Med.* **2017**, *34*, 165–169, doi:10.1016/j.ctim.2017.08.012.
25. Farshbaf-Khalili, A.; Kamalifard, M.; Namadian, M. Comparison of the effect of lavender and bitter orange on anxiety in postmenopausal women: A triple-blind, randomized, controlled clinical trial. *Complement. Ther. Clin. Pract.* **2018**, *31*, 132–138, doi:10.1016/j.ctcp.2018.02.004.
26. Hawkins, J.R.; Weatherby, N.; Wrye, B.; Ujcich Ward, K. Bergamot Aromatherapy for Medical Office–Induced Anxiety Among Children With an Autism Spectrum Disorder. *Holist. Nurs. Pract.* **2019**, *33*, 285–294, doi:10.1097/HNP.0000000000000341.
27. Jafarnia, N.; Ghorbani, Z.; Nokhostin, M.; Manayi, A.; Nourimajd, S.; Razeghi Jahromi, S. Effect of Saffron (Crocus sativus L.) as an Add-On Therapy to Sertraline in Mild to Moderate Generalized Anxiety Disorder: A Double Blind Randomized Controlled Trial. *Arch. Neurosci.* **2017**, *4*, e14332-, doi:10.5812/archneurosci.14332.
28. Mazidi, M.; Shemshian, M.; Mousavi, S.H.; Norouzy, A.; Kermani, T.; Moghiman, T.; Sadeghi, A.; Mokhber, N.; Ghayour-Mobarhan, M.; Ferns, G.A.A. A double-blind, randomized and placebo-controlled trial of Saffron (Crocus sativus L.) in the treatment of anxiety and depression. *J. Complement. Integr. Med.* **2016**, *13*, doi:10.1515/jcim-2015-0043.
29. Ghajar, A.; Neishabouri, S.; Velayati, N.; Jahangard, L.; Matinnia, N.; Haghighi, M.; Ghaleiha, A.; Afarideh, M.; Salimi, S.; Meysamie, A.; et al. Crocus sativus L. versus Citalopram in the Treatment of Major Depressive Disorder with Anxious Distress: A Double-Blind, Controlled Clinical Trial. *Pharmacopsychiatry* **2017**, *50*, 152–160, doi:10.1055/s-0042-116159.
30. Lopresti, A.L.; Drummond, P.D.; Inarejos-García, A.M.; Prodanov, M. affron®, a standardised extract from saffron (Crocus sativus L.) for the treatment of youth anxiety and depressive symptoms: A randomised, double-blind, placebo-controlled study. *J. Affect. Disord.* **2018**, *232*, 349–357, doi:10.1016/j.jad.2018.02.070.
31. Tabeshpour, J.; Sobhani, F.; Sadjadi, S.A.; Hosseinzadeh, H.; Mohajeri, S.A.; Rajabi, O.; Taherzadeh, Z.; Eslami, S. A double-blind, randomized, placebo-controlled trial of saffron stigma (Crocus sativus L .) in mothers suffering from mild-to-moderate postpartum depression. *Phytomedicine* **2017**, *36*, 145–152, doi:10.1016/j.phymed.2017.10.005.
32. Kashani, L.; Eslatmanesh, S.; Saedi, N.; Niroomand, N.; Ebrahimi, M.; Hosseinian, M.; Foroughifar, T.; Salimi, S.; Akhondzadeh, S. Comparison of Saffron versus Fluoxetine in Treatment of Mild to Moderate

Postpartum Depression: A Double-Blind, Randomized Clinical Trial. *Pharmacopsychiatry* **2017**, *50*, 64–68, doi:10.1055/s-0042-115306.

33. Lopresti, A.L.; Smith, S.J.; Hood, S.D.; Drummond, P.D. Efficacy of a standardised saffron extract (affron®) as an add-on to antidepressant medication for the treatment of persistent depressive symptoms in adults: A randomised, double-blind, placebo-controlled study. *J. Psychopharmacol.* **2019**, *33*, 1415–1427, doi:10.1177/0269881119867703.
34. Sahraian, A.; Jelodar, S.; Javid, Z.; Mowla, A.; Ahmadzadeh, L. Study the effects of saffron on depression and lipid profiles: A double blind comparative study. *Asian J. Psychiatr.* **2016**, *22*, 174–176, doi:10.1016/j.ajp.2015.10.012.
35. Jelodar, G.; Javid, Z.; Sahraian, A.; Jelodar, S. Saffron improved depression and reduced homocysteine level in patients with major depression: A Randomized, double-blind study. *Avicenna J. Phytomedicine* **2018**, *8*, 43–50.
36. Ahmadpanah, M.; Ramezanshams, F.; Ghaleiha, A.; Akhondzadeh, S.; Sadeghi Bahmani, D.; Brand, S. Crocus Sativus L. (saffron) versus sertraline on symptoms of depression among older people with major depressive disorders–a double-blind, randomized intervention study. *Psychiatry Res.* **2019**, *282*, 112613, doi:10.1016/j.psychres.2019.112613.
37. Kashani, L.; Esalatmanesh, S.; Eftekhari, F.; Salimi, S.; Foroughifar, T.; Etesam, F.; Safiaghdam, H.; Moazen-Zadeh, E.; Akhondzadeh, S. Efficacy of Crocus sativus (saffron) in treatment of major depressive disorder associated with post-menopausal hot flashes: a double-blind, randomized, placebo-controlled trial. *Arch. Gynecol. Obstet.* **2018**, *297*, 717–724, doi:10.1007/s00404-018-4655-2.
38. Jalali, F.; Hashemi, S.F. The Effect of Saffron on Depression among Recovered Consumers of Methamphetamine Living with HIV/AIDS. *Subst. Use Misuse* **2018**, *53*, 1951–1957, doi:10.1080/10826084.2018.1447583.
39. Moazen-Zadeh, E.; Abbasi, S.H.; Safi-Aghdam, H.; Shahmansouri, N.; Arjmandi-Beglar, A.; Hajhosseinn Talasaz, A.; Salehiomran, A.; Forghani, S.; Akhondzadeh, S. Effects of Saffron on Cognition, Anxiety, and Depression in Patients Undergoing Coronary Artery Bypass Grafting: A Randomized Double-Blind Placebo-Controlled Trial. *J. Altern. Complement. Med.* **2018**, *24*, 361–368, doi:10.1089/acm.2017.0173.
40. Milajerdi, A.; Jazayeri, S.; Shirzadi, E.; Hashemzadeh, N.; Azizgol, A.; Djazayery, A.; Esmaillzadeh, A.; Akhondzadeh, S. The effects of alcoholic extract of saffron (Crocus satious L.) on mild to moderate comorbid depression-anxiety, sleep quality, and life satisfaction in type 2 diabetes mellitus: A double-blind, randomized and placebo-controlled clinical trial. *Complement. Ther. Med.* **2018**, *41*, 196–202, doi:10.1016/j.ctim.2018.09.023.
41. Lopresti, A.L.; Drummond, P.D. Efficacy of curcumin, and a saffron/curcumin combination for the treatment of major depression: A randomised, double-blind, placebo-controlled study. *J. Affect. Disord.* **2017**, *207*, 188–196, doi:10.1016/j.jad.2016.09.047.
42. Lopresti, A.L.; Maes, M.; Meddens, M.J.M.; Maker, G.L.; Arnoldussen, E.; Drummond, P.D. Curcumin and major depression: A randomised, double-blind, placebo-controlled trial investigating the potential of peripheral biomarkers to predict treatment response and antidepressant mechanisms of change. *Eur. Neuropsychopharmacol.* **2015**, *25*, 38–50, doi:10.1016/j.euroneuro.2014.11.015.
43. Panahi, Y.; Badeli, R.; Karami, G.-R.; Sahebkar, A. Investigation of the Efficacy of Adjunctive Therapy with Bioavailability-Boosted Curcuminoids in Major Depressive Disorder. *Phyther. Res.* **2015**, *29*, 17–21, doi:10.1002/ptr.5211.
44. Esmaily, H.; Sahebkar, A.; Iranshahi, M.; Ganjali, S.; Mohammadi, A.; Ferns, G.; Ghayour-Mobarhan, M. An investigation of the effects of curcumin on anxiety and depression in obese individuals: A randomized controlled trial. *Chin. J. Integr. Med.* **2015**, *21*, 332–338, doi:10.1007/s11655-015-2160-z.
45. Setiawati, M.C.; Ikawati, Z.; Kertia, I.N. Antiinflamatory and antidepressive activities of Extract Curcuma xanthorrhiza Roxb in Systemic Lupus Erythematosus. *Indones. J. Pharm.* **2017**, *28*, 185, doi:10.14499/indonesianjpharm28iss3pp185.
46. Firoozabadi, A.; Zarshenas, M.M.; Salehi, A.; Jahanbin, S.; Mohagheghzadeh, A. Effectiveness of *Cuscuta planiflora* Ten. and *Nepeta menthoides* Boiss. & Buhse in Major Depression. *J. Evid. Based. Complementary Altern. Med.* **2015**, *20*, 94–97, doi:10.1177/2156587214557359.
47. Haller, J.; Krecsak, L.; Zámbori, J. Double-blind placebo controlled trial of the anxiolytic effects of a standardized Echinacea extract. *Phyther. Res.* **2020**, *34*, 660–668, doi:10.1002/ptr.6558.

48. Anushiravani, M.; Manteghi, A.A.; Taghipur, A.; Eslami, M. Comparing Effectiveness of a Combined Herbal Drug Based on Echium Amoenum with Citalopram in the Treatment of Major Depressive Disorder. *Curr. Drug Discov. Technol.* **2019**, *16*, 232–238, doi:10.2174/1570163815666180219115844.
49. Zeinalzadeh, S.; Akbarzadeh, M.; Mohagheghzadeh, A.; Faridi, P.; Sayadi, M. Comparison of the Effects of *Elaeagnus angustifolia* Flower Capsule and Sildenafil Citrate Tablet on Anxiety Resulting From Sexual Dysfunction in Women Referring to the Selected Clinics of Shiraz University of Medical Sciences. *J. Evid. Based. Complementary Altern. Med.* **2016**, *21*, 186–193, doi:10.1177/2156587215595777.
50. Ghazanfarpour, M.; Mohammadzadeh, F.; shokrollahi, P.; Khadivzadeh, T.; Najaf Najafi, M.; Hajirezaee, H.; Afiat, M. Effect of Foeniculum vulgare (fennel) on symptoms of depression and anxiety in postmenopausal women: a double-blind randomised controlled trial. *J. Obstet. Gynaecol. (Lahore).* **2018**, *38*, 121–126, doi:10.1080/01443615.2017.1342229.
51. Romero-Cerecero, O.; Islas-Garduño, A.L.; Zamilpa, A.; Herrera-Arellano, A.; Jiménez-Ferrer, E.; Tortoriello, J. Galphimine-B Standardized Extract versus Alprazolam in Patients with Generalized Anxiety Disorder: A Ten-Week, Double-Blind, Randomized Clinical Trial. *Biomed Res. Int.* **2019**, *2019*, 1037036, doi:10.1155/2019/1037036.
52. Romero-Cerecero, O.; Islas-Garduño, A.L.; Zamilpa, A.; Pérez-García, M.D.; Tortoriello, J. Therapeutic Effectiveness of *Galphimia glauca* in Young People with Social Anxiety Disorder: A Pilot Study. *Evidence-Based Complement. Altern. Med.* **2018**, *2018*, 1716939, doi:10.1155/2018/1716939.
53. Dai, C.-X.; Hu, C.-C.; Shang, Y.-S.; Xie, J. Role of Ginkgo biloba extract as an adjunctive treatment of elderly patients with depression and on the expression of serum S100B. *Medicine (Baltimore).* **2018**, *97*, e12421, doi:10.1097/MD.0000000000012421.
54. Shirzadegan, R.; Gholami, M.; Hasanvand, S.; Birjandi, M.; Beiranvand, A. Effects of geranium aroma on anxiety among patients with acute myocardial infarction: A triple-blind randomized clinical trial. *Complement. Ther. Clin. Pract.* **2017**, *29*, 201–206, doi:10.1016/j.ctcp.2017.10.005.
55. Choi, E.-K.; Won, Y.H.; Kim, S.-Y.; Noh, S.-O.; Park, S.-H.; Jung, S.-J.; Lee, C.K.; Hwang, B.Y.; Lee, M.K.; Ha, K.-C.; et al. Supplementation with extract of Gynostemma pentaphyllum leaves reduces anxiety in healthy subjects with chronic psychological stress: A randomized, double-blind, placebo-controlled clinical trial. *Phytomedicine* **2019**, *52*, 198–205, doi:10.1016/j.phymed.2018.05.002.
56. Kyrou, I.; Christou, A.; Panagiotakos, D.; Stefanaki, C.; Skenderi, K.; Katsana, K.; Tsigos, C. Effects of a hops (Humulus lupulus L.) dry extract supplement on self-reported depression, anxiety and stress levels in apparently healthy young adults: a randomized, placebo-controlled, double-blind, crossover pilot study. *Hormones* **2017**, *16*, 171–180, doi:10.14310/horm.2002.1738.
57. Seifritz, E.; Hatzinger, M.; Holsboer-Trachsler, E. Efficacy of *Hypericum* extract WS ® 5570 compared with paroxetine in patients with a moderate major depressive episode – a subgroup analysis. *Int. J. Psychiatry Clin. Pract.* **2016**, *20*, 126–132, doi:10.1080/13651501.2016.1179765.
58. Di Pierro, F.; Risso, P.; Settembre, R. Role in depression of a multi-fractionated versus a conventional Hypericum perforatum extract. *Panminerva Med.* **2018**, *60*, 156–160, doi:10.23736/S0031-0808.18.03518-8.
59. Eatemadnia, A.; Ansari, S.; Abedi, P.; Najar, S. The effect of Hypericum perforatum on postmenopausal symptoms and depression: A randomized controlled trial. *Complement. Ther. Med.* **2019**, *45*, 109–113, doi:10.1016/j.ctim.2019.05.028.
60. Abbaszadeh, R.; Tabari, F.; Asadpour, A. The Effect of Lavender Aroma on Anxiety of Patients Having Bone Marrow Biopsy. *Asian Pacific J. Cancer Prev.* **2020**, *21*, 771–775, doi:10.31557/APJCP.2020.21.3.771.
61. Karadag, E.; Samancioglu, S.; Ozden, D.; Bakir, E. Effects of aromatherapy on sleep quality and anxiety of patients. *Nurs. Crit. Care* **2017**, *22*, 105–112, doi:10.1111/nicc.12198.
62. Tugut, N.; Demirel, G.; Baser, M.; Ata, E.E.; Karakus, S. Effects of lavender scent on patients' anxiety and pain levels during gynecological examination. *Complement. Ther. Clin. Pract.* **2017**, *28*, 65–69, doi:10.1016/j.ctcp.2017.05.006.
63. Karaman, T.; Karaman, S.; Dogru, S.; Tapar, H.; Sahin, A.; Suren, M.; Arici, S.; Kaya, Z. Evaluating the efficacy of lavender aromatherapy on peripheral venous cannulation pain and anxiety: A prospective, randomized study. *Complement. Ther. Clin. Pract.* **2016**, *23*, 64–68, doi:10.1016/j.ctcp.2016.03.008.
64. Kasper, S.; Anghelescu, I.; Dienel, A. Efficacy of orally administered Silexan in patients with anxiety-related restlessness and disturbed sleep – A randomized, placebo-controlled trial. *Eur. Neuropsychopharmacol.* **2015**, *25*, 1960–1967, doi:10.1016/j.euroneuro.2015.07.024.

65. Kasper, S.; Volz, H.-P.; Dienel, A.; Schläfke, S. Efficacy of Silexan in mixed anxiety–depression – A randomized, placebo-controlled trial. *Eur. Neuropsychopharmacol.* **2016**, *26*, 331–340, doi:10.1016/j.euroneuro.2015.12.002.
66. Kasper, S.; Möller, H.-J.; Volz, H.-P.; Schläfke, S.; Dienel, A. Silexan in generalized anxiety disorder. *Int. Clin. Psychopharmacol.* **2017**, *32*, 195–204, doi:10.1097/YIC.0000000000000176.
67. Karan, N.B. Influence of lavender oil inhalation on vital signs and anxiety: A randomized clinical trial. *Physiol. Behav.* **2019**, *211*, 112676, doi:10.1016/j.physbeh.2019.112676.
68. Franco, L.; Blanck, T.J.J.; Dugan, K.; Kline, R.; Shanmugam, G.; Galotti, A.; von Bergen Granell, A.; Wajda, M. Both lavender fleur oil and unscented oil aromatherapy reduce preoperative anxiety in breast surgery patients: a randomized trial. *J. Clin. Anesth.* **2016**, *33*, 243–249, doi:10.1016/j.jclinane.2016.02.032.
69. Xiong, M.; Li, Y.; Tang, P.; Zhang, Y.; Cao, M.; Ni, J.; Xing, M. Effectiveness of Aromatherapy Massage and Inhalation on Symptoms of Depression in Chinese Community-Dwelling Older Adults. *J. Altern. Complement. Med.* **2018**, *24*, 717–724, doi:10.1089/acm.2017.0320.
70. Ozkaraman, A.; Dügüm, Ö.; Özen Yılmaz, H.; Usta Yesilbalkan, Ö. Aromatherapy: The Effect of Lavender on Anxiety and Sleep Quality in Patients Treated With Chemotherapy. *Clin. J. Oncol. Nurs.* **2018**, *22*, 203–210, doi:10.1188/18.CJON.203-210.
71. Ayik, C.; Özden, D. The effects of preoperative aromatherapy massage on anxiety and sleep quality of colorectal surgery patients: A randomized controlled study. *Complement. Ther. Med.* **2018**, *36*, 93–99, doi:10.1016/j.ctim.2017.12.002.
72. Stojanovska, L.; Law, C.; Lai, B.; Chung, T.; Nelson, K.; Day, S.; Apostolopoulos, V.; Haines, C. Maca reduces blood pressure and depression, in a pilot study in postmenopausal women. *Climacteric* **2015**, *18*, 69–78, doi:10.3109/13697137.2014.929649.
73. Keefe, J.R.; Mao, J.J.; Soeller, I.; Li, Q.S.; Amsterdam, J.D. Short-term open-label chamomile (Matricaria chamomilla L.) therapy of moderate to severe generalized anxiety disorder. *Phytomedicine* **2016**, *23*, 1699–1705, doi:10.1016/j.phymed.2016.10.013.
74. Mao, J.J.; Xie, S.X.; Keefe, J.R.; Soeller, I.; Li, Q.S.; Amsterdam, J.D. Long-term chamomile (Matricaria chamomilla L.) treatment for generalized anxiety disorder: A randomized clinical trial. *Phytomedicine* **2016**, *23*, 1735–1742, doi:10.1016/j.phymed.2016.10.012.
75. Zamanifar, S.; Bagheri-Saveh, M.I.; Nezakati, A.; Mohammadi, R.; Seidi, J. The Effect of Music Therapy and Aromatherapy with Chamomile-Lavender Essential Oil on the Anxiety of Clinical Nurses: A Randomized and Double-Blind Clinical Trial. *J. Med. Life* **2020**, *13*, 87–93.
76. Alijaniha, F.; Naseri, M.; Afsharypuor, S.; Fallahi, F.; Noorbala, A.; Mosaddegh, M.; Faghihzadeh, S.; Sadrai, S. Heart palpitation relief with Melissa officinalis leaf extract: Double blind, randomized, placebo controlled trial of efficacy and safety. *J. Ethnopharmacol.* **2015**, *164*, 378–384, doi:10.1016/j.jep.2015.02.007.
77. Haybar, H.; Javid, A.Z.; Haghighizadeh, M.H.; Valizadeh, E.; Mohaghegh, S.M.; Mohammadzadeh, A. The effects of Melissa officinalis supplementation on depression, anxiety, stress, and sleep disorder in patients with chronic stable angina. *Clin. Nutr. ESPEN* **2018**, *26*, 47–52, doi:10.1016/j.clnesp.2018.04.015.
78. Kennedy, D.; Okello, E.; Chazot, P.; Howes, M.-J.; Ohiomokhare, S.; Jackson, P.; Haskell-Ramsay, C.; Khan, J.; Forster, J.; Wightman, E. Volatile Terpenes and Brain Function: Investigation of the Cognitive and Mood Effects of Mentha × Piperita L. Essential Oil with In Vitro Properties Relevant to Central Nervous System Function. *Nutrients* **2018**, *10*, 1029, doi:10.3390/nu10081029.
79. Kolouri, S.; Firoozabadi, A.; Salehi, A.; Zarshenas, M.M.; Dastgheib, S.A.; Heydari, M.; Rezaeizadeh, H. Nepeta menthoides Boiss. & Buhse freeze-dried aqueous extract versus sertraline in the treatment of major depression: A double blind randomized controlled trial. *Complement. Ther. Med.* **2016**, *26*, 164–170, doi:10.1016/j.ctim.2016.03.016.
80. Jeong, H.-G.; Ko, Y.-H.; Oh, S.-Y.; Han, C.; Kim, T.; Joe, S.-H. Effect of Korean Red Ginseng as an adjuvant treatment for women with residual symptoms of major depression. *Asia-Pacific Psychiatry* **2015**, *7*, 330–336, doi:10.1111/appy.12169.
81. Dantas, L.; de Oliveira-Ribeiro, A.; de Almeida-Souza, L.; Groppo, F. Effects of passiflora incarnata and midazolam for control of anxiety in patients undergoing dental extraction. *Med. Oral Patol. Oral y Cir. Bucal* **2017**, *22*, e95–e101, doi:10.4317/medoral.21140.

82. Savage, K.M.; Stough, C.K.; Byrne, G.J.; Scholey, A.; Bousman, C.; Murphy, J.; Macdonald, P.; Suo, C.; Hughes, M.; Thomas, S.; et al. Kava for the treatment of generalised anxiety disorder (K-GAD): study protocol for a randomised controlled trial. *Trials* **2015**, *16*, 493, doi:10.1186/s13063-015-0986-5.
83. Hamdamian, S.; Nazarpour, S.; Simbar, M.; Hajian, S.; Mojab, F.; Talebi, A. Effects of aromatherapy with Rosa damascena on nulliparous women's pain and anxiety of labor during first stage of labor. *J. Integr. Med.* **2018**, *16*, 120–125, doi:10.1016/j.joim.2018.02.005.
84. Fazlollahpour-Rokni, F.; Shorofi, S.A.; Mousavinasab, N.; Ghafari, R.; Esmaeili, R. The effect of inhalation aromatherapy with rose essential oil on the anxiety of patients undergoing coronary artery bypass graft surgery. *Complement. Ther. Clin. Pract.* **2019**, *34*, 201–207.
85. Daneshpajooh, L.; Najafi Ghezeljeh, T.; Haghani, H. Comparison of the effects of inhalation aromatherapy using Damask Rose aroma and the Benson relaxation technique in burn patients: A randomized clinical trial. *Burns* **2019**, *45*, 1205–1214, doi:10.1016/j.burns.2019.03.001.
86. Mao, J.J.; Xie, S.X.; Zee, J.; Soeller, I.; Li, Q.S.; Rockwell, K.; Amsterdam, J.D. Rhodiola rosea versus sertraline for major depressive disorder: A randomized placebo-controlled trial. *Phytomedicine* **2015**, *22*, 394–399, doi:10.1016/j.phymed.2015.01.010.
87. Cropley, M.; Banks, A.P.; Boyle, J. The Effects of *Rhodiola rosea* L. Extract on Anxiety, Stress, Cognition and Other Mood Symptoms. *Phyther. Res.* **2015**, *29*, 1934–1939, doi:10.1002/ptr.5486.
88. Lopresti, A.L.; Smith, S.J.; Malvi, H.; Kodgule, R. An investigation into the stress-relieving and pharmacological actions of an ashwagandha (Withania somnifera) extract. *Medicine (Baltimore).* **2019**, *98*, e17186, doi:10.1097/MD.0000000000017186.
89. Fuladi, S.; Emami, S.A.; Mohammadpour, A.H.; Karimani, A.; Manteghi, A.A.; Sahebkar, A. Assessment of Withania somnifera root extract efficacy in patients with generalized anxiety disorder: A randomized double-blind placebo-controlled trial. *Curr. Clin. Pharmacol.* **2020**, *15*, doi:10.2174/1574884715666200413120413.

© 2021 by the author. Licensee MDPI, Basel, Switzerland. This article is an open access article distributed under the terms and conditions of the Creative Commons Attribution (CC BY) license (http://creativecommons.org/licenses/by/4.0/).
